# Photocatalytic Iminyl Radical Cyclization for the Synthesis of Quinazolinones

**DOI:** 10.1021/acs.joc.6c00247

**Published:** 2026-05-30

**Authors:** Neissa Usanase, Jensen L. Rocha, Joyce J. Yoo, Hanbit J. Lee, Hannah A. Spencer, Mohammed D. Albotabeekh, Janeth A. Sandoval, Erin E. Gray

**Affiliations:** Department of Chemistry and Biochemistry, 4531 Washington and Lee University, Lexington, Virginia 24450, United States

## Abstract

An operationally simple protocol for the synthesis of medicinally relevant quinazolin-4­(3*H*)-ones is described. This method employs an organic dye, ambient atmosphere, and visible light to activate α-azido benzamides through a photoinduced hydrogen atom transfer, generating iminyl radicals upon extrusion of dinitrogen. Cyclization to close the pyrimidinone ring then affords a variety of quinazolinone derivatives. This photocatalytic strategy establishes a foundation for iminyl radical-mediated heterocycle synthesis from alkyl azides under mild conditions.

## Graphical abstract

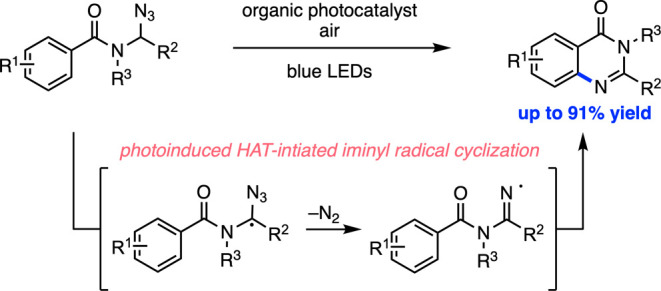

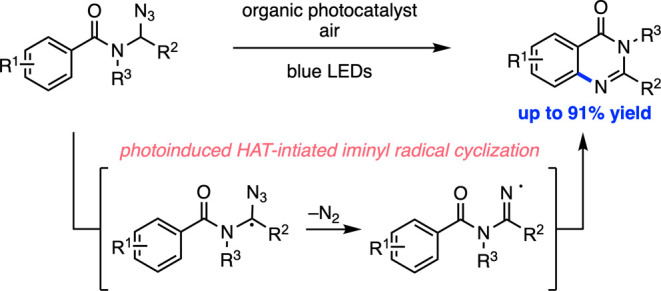

The quinazolinone scaffold, which consists of fused benzene and pyrimidinone rings, is found in compounds with wide-ranging biological properties, including anticancer, anti-inflammatory, and antimicrobial activities (Figure 1).^1^ Among the structurally diverse quinazolinone derivatives, quinazolin-4­(3*H*)-ones have emerged as privileged structures in drug discovery, prompting the development of innovative synthetic strategies for their preparation and evaluation of their medicinal potential.^2^ Despite significant progress, most available methods to construct quinazolin-4­(3*H*)-ones still rely on *ortho*-functionalized aniline or benzoic acid derivatives as starting materials. Complementary approaches employing simple, readily available monosubstituted benzene precursors offer a more efficient and atom-economical strategy for accessing this important heterocyclic motif. To this end, transition metal-catalyzed C–H functionalization and annulation protocols, as well as electrophilic aromatic substitution routes, have been reported; however, these methods require elevated or cryogenic temperatures, costly catalysts, and/or stoichiometric reagents.^3,4^

**Figure 1:**
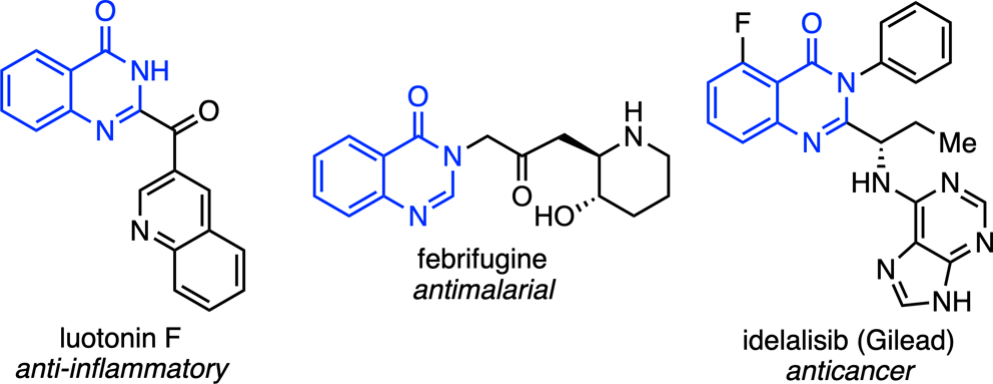
Quinazolin-4­(3*H*)-one scaffold in biologically active natural products and pharmaceuticals.

Iminyl radical cyclizations provide a valuable and mechanistically distinct pathway to close the pyrimidinone ring. Malacria and co-workers first demonstrated this type of intramolecular carbon–nitrogen bond formation in the assembly of pyrroloquinazolinone structures, relying on toxic organotin reagents to mediate the radical cascade of *N*-cyanamides (Figure 2A).^5^ Although related methods with more sustainable conditions have since been developed, existing cascade strategies using *N*-cyanamide precursors typically require the addition of radical intermediates to a tethered functional group, limiting their application to the synthesis of 2,3-fused quinazolinones.^6^ Similarly, oxidative radical skeletal rearrangements of 1,2,4-oxadiazolines proceed via an iminyl radical intermediate and afford only 2-aryl and 2-alkyl quinazolinones under either thermal or electrochemical conditions (Figure 2B).^7,8^ An alternative approach was previously disclosed by Yu and co-workers, involving the radical cyclization of α-azido benzamides to furnish a range of quinazolin-4­(3*H*)-one derivatives (Figure 2C).^9^ In this transformation, α-azido radicals formed through hydrogen atom transfer (HAT) undergo dinitrogen extrusion to generate the requisite iminyl radical. The use of superstoichiometric quantities of *N*-bromosuccinimide (NBS), however, compromises the atom economy of this reaction.

**Figure 2:**
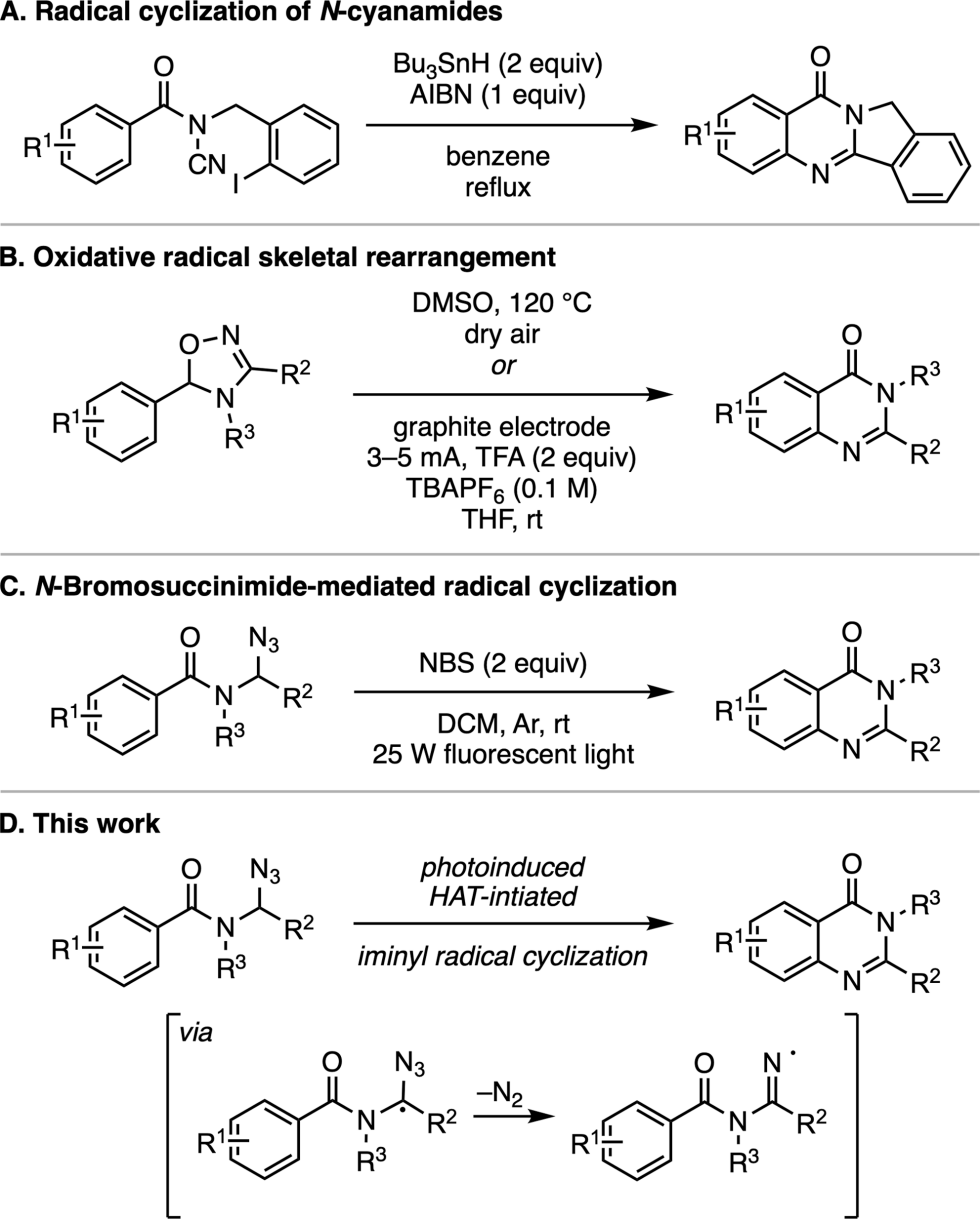
Iminyl radical cyclization strategies for the synthesis of quinazolinones.

Inspired by this work, we designed a strategy to generate iminyl radical intermediates via a photocatalytic HAT protocol (Figure 2D). Carbon-centered radical generation via HAT is an enabling and prevalent feature of photocatalytic technologies,^10^ yet initiating a radical cascade to access iminyl radicals through this mechanistic regime remains comparatively underexplored.^11–13^ Herein, we report the application of this approach to the synthesis of quinazolin-4­(3*H*)-ones from simple starting materials under mild reaction conditions, thereby expanding the scope of radical-mediated heterocycle construction.

We began our studies by evaluating the intramolecular cyclization of α-azido fluorobenzamide **1a** in the presence of various photocatalysts capable of direct HAT. Although trace product formation was observed with several catalysts,^14^ neutral eosin Y (2′,4′,5′,7′-tetrabromofluorescein), an organic photosensitizer and dye, exhibited the most promising results under ambient aerobic conditions and blue light irradiation (Table 1, entries 1 and 2). Moreover, analysis of the crude reaction mixture by ^19^F NMR spectroscopy revealed that the remaining mass balance consisted primarily of unreacted substrate and a single oxidized side product, whereas other photocatalysts resulted in a complex mixture of products. Notably, switching the reaction solvent to 1,2-dichloroethane (DCE) significantly improved the yield, affording the desired quinazolinone **2a** in 63% yield (entries 3 and 4). Given the pH dependence of the photophysical properties of fluorescein-derived dyes,^15^ we also surveyed a series of acid and base additives. Bro̷nsted acids have previously been shown to enhance the direct HAT reactivity of eosin Y;^16^ however, we observed no consistent trend across the additives tested (entries 5–8). Instead, the disodium salt of eosin Y was chosen as the optimal and more cost-effective catalyst (entry 9).

**Table 1:**
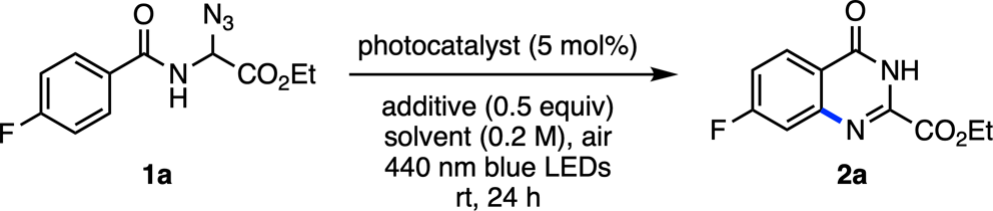
Reaction Optimization

entry	photocatalyst	additive	solvent	yield (%)^a^
1^b^	eosin Y	–	MeCN	3
2	eosin Y	–	MeCN	26
3	eosin Y	–	DCM	45
4	eosin Y	–	DCE	63
5	eosin Y	TFA	DCE	64
6	eosin Y	*p*-TsOH·H_2_O	DCE	44
7	eosin Y	NaH_2_PO_4_·H_2_O	DCE	71
8	eosin Y	NaOH	DCE	39
9^c^	Na_2_·eosin Y	–	DCE	74

deviation from optimal conditions (entry 9)		
10	no photocatalyst	0
11	no light	0
12	525 nm green LEDs	3
13^b^	sealed under N_2_ atmosphere	3
14^b^	1 equiv *t*-BuOOH	8
15^b^	1 equiv PhI(OAc)_2_	8

a Reactions were conducted on a 0.04 mmol scale, and yields were determined by ^19^F NMR spectroscopy using 1,4-dibromo-2,5-difluorobenzene as an external standard.

b Reaction vial was evacuated and backfilled with N_2_, and sealed with Parafilm after reaction setup.

c Reaction was conducted using 2.5 mol % Na_2_·eosin Y.

Control experiments confirmed that both the photocatalyst and light are necessary to generate the quinazolinone product (entries 10 and 11). Performing the reaction under green light irradiation (525 nm), which corresponds to the absorption maximum of Na_2_·eosin Y, led to a significantly lower yield than with blue light (440 nm) (entry 12). Furthermore, poor conversion was observed when the reaction was conducted under an inert atmosphere or with alternative stoichiometric oxidants such as *t*-BuOOH or PhI­(OAc)_2_, suggesting that molecular oxygen plays a critical role in this process (entries 13–15).

Having established the optimal reaction conditions, we next evaluated the scope of quinazolinone derivatives that are accessible using this protocol (Table 2). On a preparative scale, extended reaction times were required for complete consumption of model substrate **1a**, though the rate of the reaction can be accelerated by increasing the light intensity.^17^ Gratifyingly, substrate **1a** furnished product **2a** in 80% isolated yield, and simple, unsubstituted α-azido benzamide **1b** also performed well in this transformation. When toluamide derivative **1c** was subjected to the photocatalytic conditions, however, oxidation of the benzylic C–H bonds predominated, and the desired 7-methyl quinazolinone **2c** was not formed. In contrast, *tert*-butyl analog **1d** was amenable to the cyclization and avoids undesired benzylic oxidation.

**Table 2:**
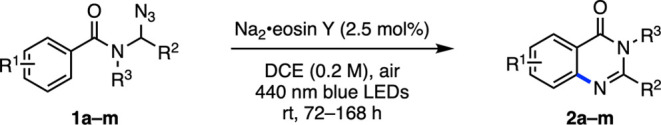

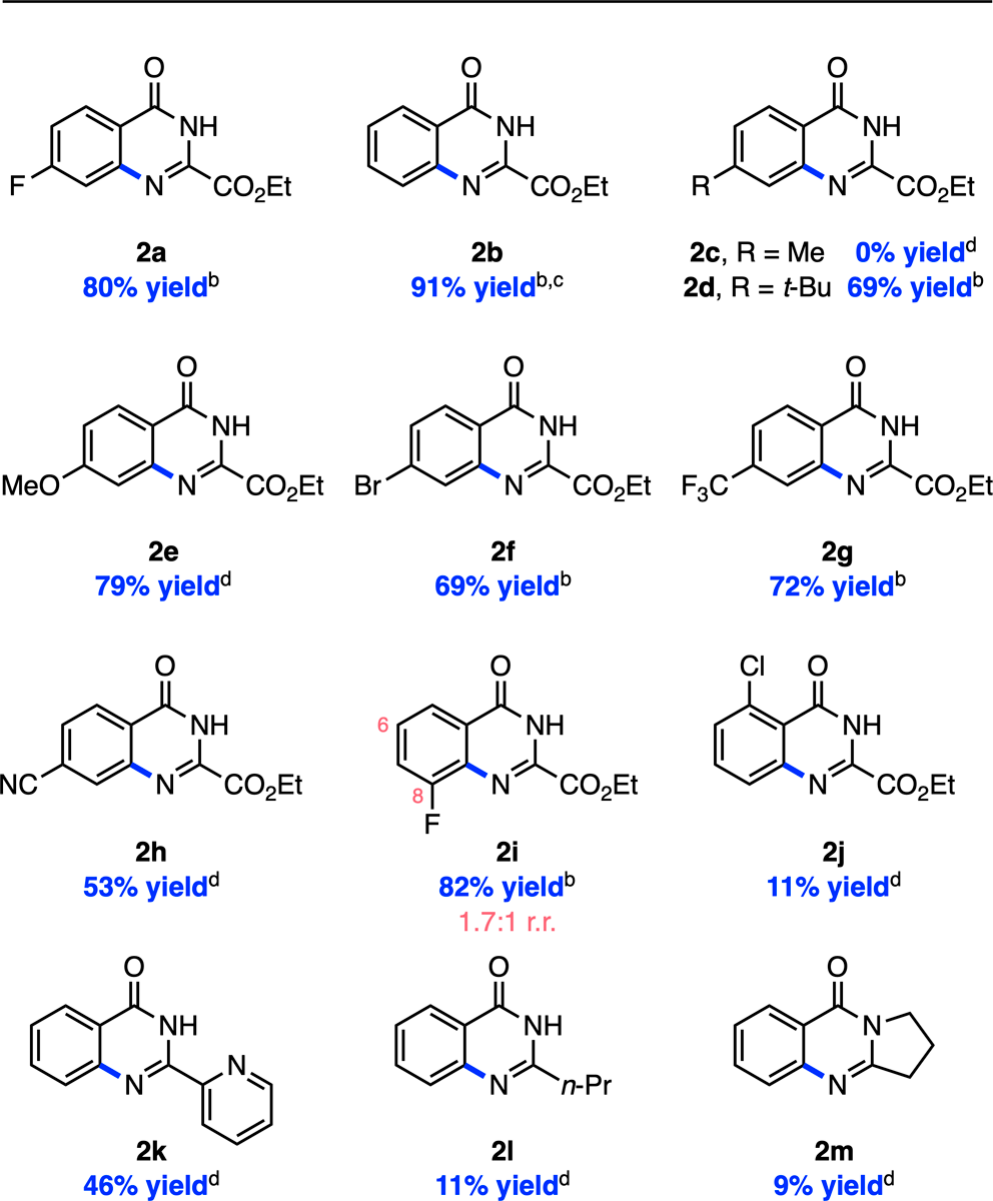
Scope of Iminyl Radical Cyclization^a^

a Reactions were conducted on a 0.5 mmol scale, and isolated yields are reported as an average of two trials.

b Reaction was irradiated for 72 h.

c Isolated 79% yield on a single 1.0 mmol scale experiment.

d Reaction was irradiated for 168 h.

Varying the electronics of the *para* substituent on the aryl ring demonstrated that both electron-rich (**1d** and **1e**) and electron-deficient (**1f**–**1h**) α-azido benzamides perform well in this transformation. Of note, the cyclization proceeded most efficiently with electronically neutral iminyl radical acceptors, while those bearing the strongest electron-donating and -withdrawing groups resulted in incomplete conversion of starting material. A *meta*-substituted benzamide derivative produced a 1.7:1 mixture of quinazolinone regioisomers, favoring the 8-substituted product **2i**, in a combined 82% yield. Importantly, *ortho*-substituted substrates are ineffective in the NBS-mediated conditions reported by Yu.^9^ Therefore, we were pleased to find that substitution at the *ortho* position was tolerated, providing product **2j**, albeit in 11% yield.

Although the ester provides a useful functional group handle for further derivatization at the 2-position, we next assessed the applicability of this strategy to the synthesis of other 2-substituted quinazolinones. Encouragingly, cyclization of pyridine substrate **1k** afforded the corresponding 2-pyridyl-substituted quinazolin-4­(3*H*)-one in moderate yield. For azidoalkyl benzamide **1l**, the photocatalytic annulation proceeded to furnish product **2l** in low yield, whereas only decomposition was reported under the NBS conditions.^9^ Access to 2,3-fused quinazolinone derivatives remains limited with the current method, as pyrroloquinazolinone **2m** was isolated in only 9% yield. In general, the reaction delivers product along with recovered starting material, though products resulting from oxidation of the carbon bearing the azido group are observed with certain substrates. Moreover, this transformation is readily conducted on the benchtop under ambient atmosphere at room temperature and without the need for stoichiometric reagents, highlighting the mild and practical conditions of this protocol compared to existing methods.

Several key observations during reaction development prompted us to examine the roles of eosin Y and oxygen in this process. First, we noted a visible color change over the course of the reaction; in the cyclization of model substrate **1a**, analysis of the reaction mixture by UV–vis spectroscopy indicated that eosin Y undergoes photodegradation under our standard conditions.^14,18^ Furthermore, monitoring the reaction progress as a function of time revealed an induction period (Figure 3A), which may correspond to the generation of an active catalyst or reactive intermediate. Consistent with the intermediacy of a radical species, the reaction was inhibited upon addition of radical scavengers TEMPO and BHT (Figure 3B). Photoexcited xanthene dyes are known to generate singlet oxygen and superoxide radical anion,^15^ and given that aerobic conditions are necessary for efficient cyclization, we hypothesized that reactive oxygen species play an important role in the catalytic cycle. Quenching experiments with 1,4-diazabicyclo[2.2.2]­octane (DABCO) and benzoquinone, targeting singlet oxygen and superoxide respectively, resulted in only trace product formation (Figure 3C). While these results implicate reactive oxygen species in this transformation, their precise structure and mechanistic role—whether radical cascade initiation, catalyst activation, eosin Y regeneration, rearomatization, or some combination thereof—remain to be determined.

**Figure 3:**
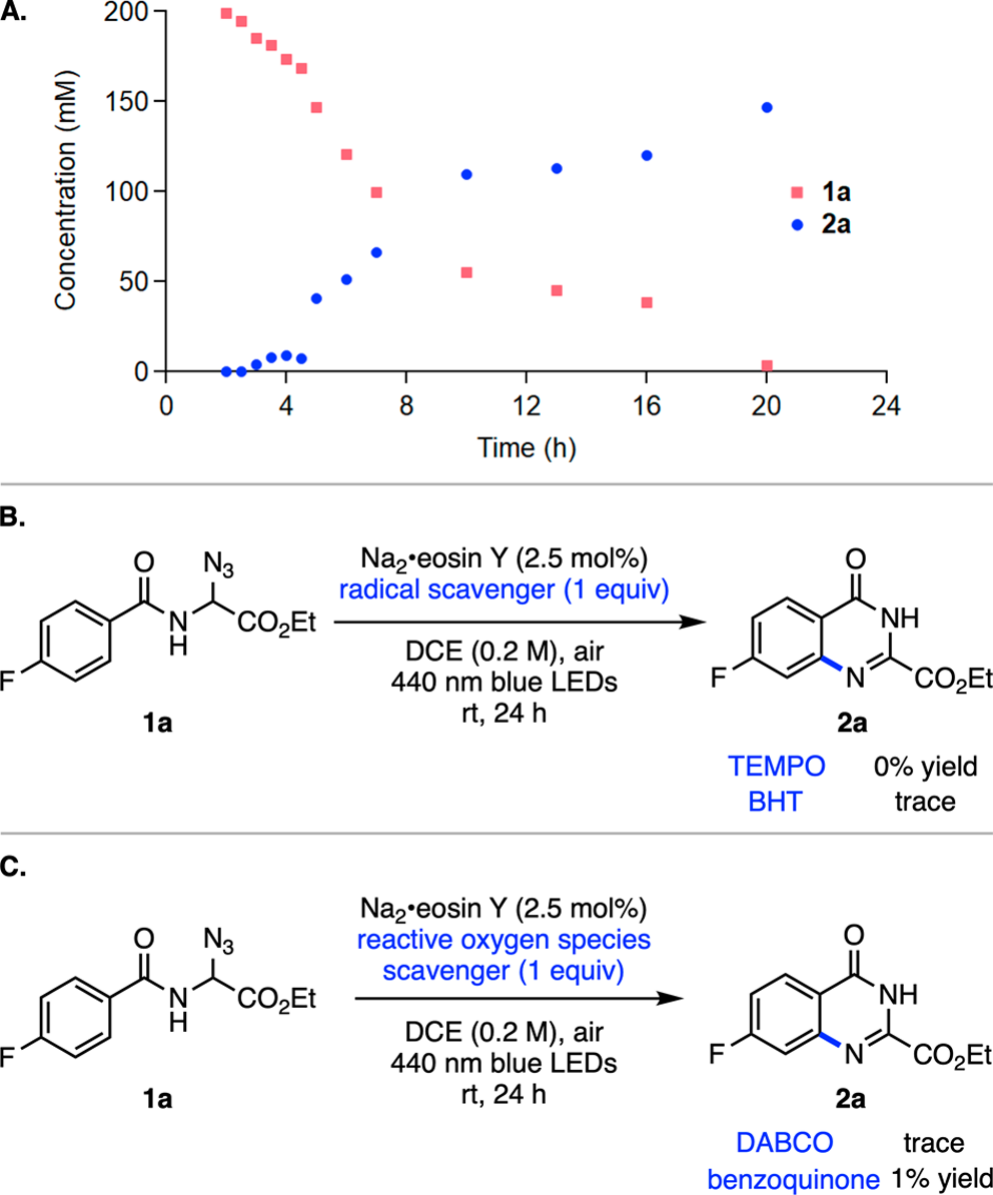
(A) Reaction profile of the iminyl radical cyclization. Each data point represents the average of discrete experiments terminated at the indicated time. Experiments conducted in the presence of radical scavengers (B) and scavengers of reactive oxygen species (C). Reactions were conducted on a 0.04 mmol scale, and yields were determined by ^19^F NMR spectroscopy using 1,4-dibromo-2,5-difluorobenzene as an external standard.

In summary, we have developed a photocatalytic iminyl radical cyclization for the synthesis of medicinally relevant quinazolin-4­(3*H*)-ones. This strategy employs an organic dye, ambient atmosphere, and visible light to generate iminyl radicals from simple, aliphatic azides under exceptionally mild conditions. The operational simplicity and distinctive mode of iminyl radical initiation in this transformation highlight its potential as a platform for heterocycle construction. The generality and mechanism of this photoinduced HAT strategy for accessing iminyl radicals will be the focus of future investigations.

## Safety Statement

Organic azides are potentially explosive and should be handled with caution. Safety precautions were employed throughout substrate selection, preparation, isolation, and handling.

## Supplementary Material



## Data Availability

The data underlying this study are available in the published article and its Supporting Information.

## Notes

Experimental procedures, characterization data, and NMR spectra for new compounds (PDF)
