# Supplementary material for: Photocatalytic Iminyl Radical Cyclization for the Synthesis of Quinazolinones
Source: J Org Chem. 2026 May 30;91(23):8052–6. doi: 10.1021/acs.joc.6c00247 (PMC13270525; doi:10.1021/acs.joc.6c00247)
Supplement: Supplementary file 1 [file jo6c00247_si_001.pdf]

Supporting Information

Photocatalytic Iminyl Radical Cyclization for the Synthesis of Quinazolinones

Neissa Usanase, Jensen L. Rocha, Joyce J. Yoo, Hanbit J. Lee, Hannah A. Spencer, Mohammed D. Albotabeekh, Janeth A. Sandoval, and Erin E. Gray*

Department of Chemistry and Biochemistry, Washington and Lee University, Lexington, VA 24450, United States

*Email: egray@wlu.edu

Table of Contents

I.	General Information	S2
II.	Synthesis and Characterization of Starting Materials	S3
III.	General Procedure for Iminyl Radical Cyclization	S12
	Synthesis and Characterization of Products	S13
IV.	Additional Experiments	S20
	Optimization Studies	S20
	Reaction Profile and Photodegradation Experiments	S24
	Preliminary Mechanistic Investigations	S25
V.	NMR Spectra	S26
VI.	References	S61

I. General Information

General Procedures. Unless otherwise noted, all reactions were carried out in oven-dried glassware under a nitrogen atmosphere using anhydrous solvents. Light-promoted reactions were performed in clear borosilicate glass reaction vessels. Air- and moisture-sensitive reagents were transferred via nitrogen-flushed stainless steel needles and plastic syringes. Reactions were monitored by thin-layer chromatography (TLC) on MilliporeSigma silica gel 60 F_{254} plates. Visualization of the developed chromatogram was performed by irradiation with UV light (254 nm). If compounds were unstable on TLC, then reaction progress was monitored by proton nuclear magnetic resonance (^{1}H NMR) spectroscopy. Organic solutions were concentrated under reduced pressure using a rotary evaporator (25–30 °C, <50 torr). Flash column chromatography was performed using SiliCycle SiliaFlash P60 silica gel (40–63 µm, 60 Å).

Safety Precautions. It should be noted that small organic azides are potentially explosive compounds, and while no explosions were encountered with the α-azido benzamides used in this study, proper precautions should be taken in substrate selection, preparation, isolation, storage, and handling.[1,2]

Instrumentation. Proton nuclear magnetic resonance (^{1}H NMR), carbon nuclear magnetic resonance (^{13}C NMR), and fluorine nuclear magnetic resonance (^{19}F NMR) spectra were recorded on a JEOL ECZS spectrometer (400, 101, and 376 MHz, respectively). Chemical shifts for proton are reported in parts per million (ppm) downfield from tetramethylsilane and are referenced to residual protium in the NMR solvent ($CHCl_3$ = δ 7.26 ppm, DMSO = δ 2.50 ppm). Chemical shifts for carbon are reported in parts per million downfield from tetramethylsilane and are referenced to the carbon resonances of the solvent residual peak ($CDCl_3$ = δ 77.16 ppm, DMSO-d_6 = δ 39.52 ppm). Chemical shifts for fluorine are reported in parts per million. NMR data are represented as follows: chemical shift (δ ppm), multiplicity (s = singlet, d = doublet, t = triplet, q = quartet, p = pentet, m = multiplet), coupling constant in Hertz (Hz), integration. Fourier transform infrared (FT-IR) spectra were recorded on a Perkin-Elmer Spectrum 100 and are reported in terms of wavenumber (cm^{-1}). High-resolution mass spectra (HRMS) were obtained at the University of Virginia using an Orbitrap Exploris 240 mass spectrometer or at Davidson College using an Agilent 6520B Q-TOF mass spectrometer, both with an electrospray ionization (ESI) source in positive-ion mode. Melting points were measured on an Electrothermal Mel-Temp apparatus in open capillary tubes and are uncorrected. Ultraviolet–visible (UV–vis) spectra were recorded on a Hewlett-Packard 8453 UV–visible spectrophotometer.

Materials. Commercial reagents were purchased from Thermo Fisher Scientific, Sigma-Aldrich, TCI, Acros, or Alfa Aesar and used as received. Eosin Y disodium salt (Na_2·eosin Y, acid red 87, 90.0+%) was purchased from TCI and stored in a desiccator containing $CaSO_4$. Anhydrous 1,2-dichloroethane (DCE) was purchased from Sigma-Aldrich and used as received.

Light Sources. Unless otherwise noted, all photoreactions were carried out using Kessil PR160L-440 LED lamps (100% intensity; Kessil Lighting, DiCon Fiberoptics, Inc.) without additional optical filters. LED lamps were placed 2 cm away from reaction vessels with a cooling fan.

II. Synthesis and Characterization of Starting Materials

α-Azido benzamide substrates were prepared following modified literature procedures.[3]

Route A:

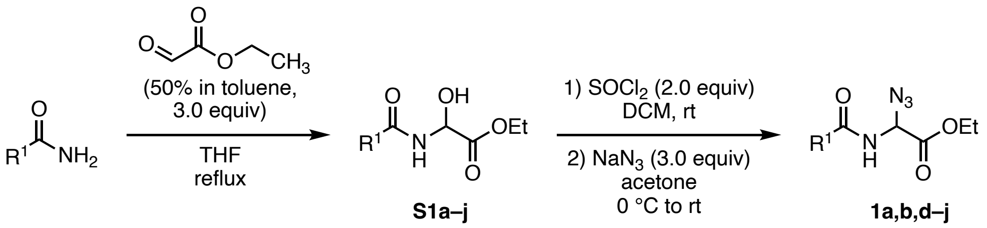

General Procedure A1: Preparation of *N*,*O*-aminals[4]

To a round bottom flask fitted with a reflux condenser was added benzamide (1.0 equiv), ethyl glyoxylate (50% solution in toluene, 3.0 equiv), and tetrahydrofuran (0.23 M). The solution was stirred at 80 °C for 16 h and then cooled to room temperature before concentrating under reduced pressure. The material was then purified by trituration or column chromatography on silica gel.

General Procedure A2: Nucleophilic substitution with azide

To a solution of *N*,*O*-aminal (1.0 equiv) in dichloromethane (0.5 M) was added thionyl chloride (2.0 equiv). The resulting mixture was stirred at room temperature for 4 h, or until starting material was consumed, and then the reaction was concentrated under reduced pressure.[5] The resulting residue was cooled to 0 ºC before NaN_3 (3.0 equiv) was added in one portion and the mixture was diluted with acetone (0.1 M). The reaction mixture was allowed to stir overnight, gradually warming to room temperature. Upon completion, the reaction mixture was concentrated under reduced pressure, and the residue was purified by column chromatography on silica gel to afford the desired azide product.

Route B:

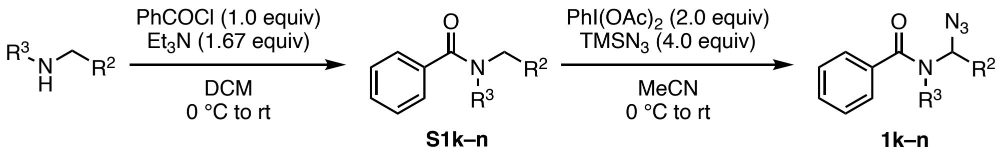

General Procedure B1: Preparation of amides

To a flame-dried round bottom flask was added amine (1.0 equiv) and triethylamine (1.67 equiv) in dichloromethane (0.5 M). The resulting solution was cooled to 0 ºC, and a solution of benzoyl chloride (1.0 equiv in 1.5 M dichloromethane) was added dropwise. The reaction mixture was allowed to stir overnight, gradually warming to room temperature. Upon completion, the reaction was quenched with sat. $NaHCO_{3(aq)}$ solution and then extracted with dichloromethane (3×). The combined organic layers were washed with brine, dried over $MgSO_4$, filtered, and concentrated under reduced pressure. The crude reside was used without further purification.

General Procedure B2: Radical azidation[6]

A flame-dried round bottom flask equipped with a stir bar, nitrogen inlet, and septum was charged with amide (1.0 equiv) and (diacetoxyiodo)benzene (2.0 equiv). The flask was then evacuated and backfilled with nitrogen (3×) and cooled with an ice bath before acetonitrile (0.1 M) was added. $TMSN_3$ (4.0 equiv) was then added to the reaction mixture at 0 ºC dropwise via syringe, and the reaction was allowed to stir overnight under a N_2 atmosphere, gradually warming to room temperature. The reaction mixture was then concentrated under reduced pressure, and the residue was purified by column chromatography on silica gel to afford the desired azide product.

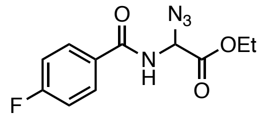

1a

ethyl 2-azido-2-(4-fluorobenzamido)acetate (1a): Synthesized following **Route A**.

Ethyl 2-(4-fluorobenzamido)-2-hydroxyacetate (**S1a**) was prepared according to general procedure A1 using 4-fluorobenzamide (5.57 g, 40.0 mmol, Matrix Scientific). Trituration with 30% EtOAc/hexanes afforded the *N,O*-aminal as a white solid (5.64 g, 58% yield). Characterization data are congruent with literature reports.[7]

^{1}H NMR (400 MHz, DMSO-*d*$_6$) δ 9.39 (d, *J* = 7.8 Hz, 1H), 8.02–7.93 (m, 2H), 7.36–7.26 (m, 2H), 6.61 (d, *J* = 6.5 Hz, 1H), 5.63 (dd, *J* = 7.8, 6.5 Hz, 1H), 4.14 (q, *J* = 7.1 Hz, 2H), 1.20 (t, *J* = 7.1 Hz, 3H).
^{13}C{^{1}H} NMR (101 MHz, DMSO-*d*$_6$) δ 169.9, 164.9, 164.2 (d, *J* = 249.0 Hz), 130.3 (d, *J* = 9.1 Hz), 130.0 (d, *J* = 3.0 Hz), 115.4 (d, *J* = 21.7 Hz), 72.0, 60.8, 14.1.
^{19}F NMR (376 MHz, DMSO-*d*$_6$) δ –108.57 (tt, *J* = 8.9, 5.5 Hz, 1F).
IR (ATR, cm^{-1}) 3385, 3320, 3075, 2987, 2944, 2908, 1746, 1642, 1603, 1542, 1504

The title compound **1a** was prepared according to general procedure A2 using ethyl 2-(4-fluorobenzamido)-2-hydroxyacetate (**S1a**, 1.45 g, 6.0 mmol). Purification by column chromatography on silica gel (gradient elution 5 to 30% EtOAc/hexanes) afforded the product as a white solid (1.28 g, 80% yield). Characterization data are congruent with literature reports.[6]

^{1}H NMR (400 MHz, CDCl$_3$) δ 7.90–7.83 (m, 2H), 7.24–7.12 (m, 3H), 5.97 (d, *J* = 7.9 Hz, 1H), 4.36 (q, *J* = 7.2 Hz, 2H), 1.38 (t, *J* = 7.2 Hz, 3H).
^{13}C{^{1}H} NMR (101 MHz, CDCl$_3$) δ 167.0, 166.3, 165.5 (d, *J* = 253.8 Hz), 130.0 (d, *J* = 9.1 Hz), 128.8 (d, *J* = 3.4 Hz), 116.1 (d, *J* = 22.1 Hz), 65.3, 63.5, 14.2.
^{19}F NMR (376 MHz, CDCl$_3$) δ –105.89 (tt, *J* = 8.3, 5.2 Hz, 1F).
IR (ATR, cm^{-1}) 3306, 2996, 2964, 2103, 1744, 1653, 1602, 1526, 1499

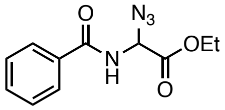

1b

ethyl 2-azido-2-benzamidoacetate (1b): Synthesized following **Route A**.

Ethyl 2-benzamido-2-hydroxyacetate (**S1b**) was prepared according to general procedure A1 using benzamide (1.45 g, 12.0 mmol, TCI). Trituration with 30% EtOAc/hexanes afforded the *N*,*O*-aminal as a white solid (1.67 g, 62% yield). Characterization data are congruent with literature reports.[7]

^{1}H NMR (400 MHz, DMSO-*d*$_6$) δ 9.36 (d, *J* = 7.8 Hz, 1H), 7.95–7.84 (m, 2H), 7.60–7.52 (m, 1H), 7.52–7.43 (m, 2H), 6.58 (d, *J* = 6.5 Hz, 1H), 5.63 (dd, *J* = 7.9, 6.5 Hz, 1H), 4.14 (q, *J* = 7.1 Hz, 2H), 1.20 (t, *J* = 7.1 Hz, 3H)
^{13}C{^{1}H} NMR (101 MHz, DMSO-*d*$_6$) δ 170.0, 166.0, 133.5, 131.8, 128.4, 127.6, 71.9, 60.8, 14.1.
IR (ATR, cm^{-1}) 3375, 3308, 2940, 1752, 1721, 1646, 1637, 1602, 1582, 1537

The title compound **1b** was prepared according to general procedure A2 using ethyl 2-benzamido-2-hydroxyacetate (**S1b**, 447 mg, 2.0 mmol). Purification by column chromatography on silica gel (gradient elution 0 to 20% EtOAc/hexanes) afforded the product as a white solid (271 mg, 55% yield). Characterization data are congruent with literature reports.[3]

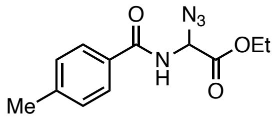

1c

Ethyl 2-azido-2-(4-methylbenzamido)acetate (**1c**) was prepared according to previously reported procedures, and spectroscopic data are in agreement with those previously reported.[8]

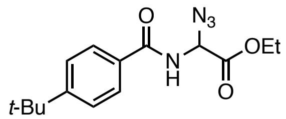

1d

ethyl 2-azido-2-(4-(*tert*-butyl)benzamido)acetate (1d): Synthesized following **Route A**.

Ethyl 2-(4-(*tert*-butyl)benzamido)-2-hydroxyacetate (**S1d**) was prepared according to general procedure A1 from 4-*tert*-butylbenzamide (1.06 g, 6.0 mmol, Thermo Scientific). Purification by column chromatography on silica gel (isocratic elution with 20% EtOAc/hexanes) afforded the *N*,*O*-aminal as a clear, viscous oil (1.14 g, 68% yield).

^{1}H NMR (400 MHz, DMSO-*d*$_6$) δ 9.26 (d, *J* = 7.9 Hz, 1H), 7.86–7.80 (m, 2H), 7.51–7.45 (m, 2H), 6.54 (d, *J* = 6.6 Hz, 1H), 5.62 (dd, *J* = 7.9, 6.5 Hz, 1H), 4.13 (q, *J* = 7.1 Hz, 2H), 1.29 (s, 9H), 1.19 (t, *J* = 7.1 Hz, 3H).

$^{13}C\{^1H\}$ **NMR (101 MHz, DMSO-*d*$_6$)** δ 170.1, 165.9, 154.6, 130.8, 127.4, 125.2, 71.9, 60.8, 34.7, 30.9, 14.1.
IR (thin film, cm^{-1}) 3481, 3336, 2964, 2907, 1734, 1648, 1611, 1533, 1499
HRMS (ESI) calculated for $C_{15}H_{22}NO_4^+$ $[M+H]^+$ 280.1543; found: 280.1556
$\mathbf{R}_f$ = 0.20 in 40% EtOAc/hexanes

The title compound **1d** was prepared according to general procedure A2 using ethyl 2-(4-(*tert*-butyl)benzamido)-2-hydroxyacetate (**S1d**, 1.12 g, 4 mmol). Purification by column chromatography on silica gel (gradient elution 5 to 15% EtOAc/hexanes) afforded the product as a white solid (401 mg, 33% yield). Characterization data are congruent with literature reports.[8]

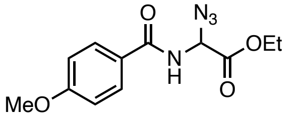

1e

ethyl 2-azido-2-(4-methoxybenzamido)acetate (1e): Synthesized following **Route A**.

Ethyl 2-hydroxy-2-(4-methoxybenzamido)acetate (**S1e**) was prepared according to general procedure A1 from 4-methoxybenzamide (756 mg, 5.0 mmol, TCI). Purification by column chromatography on silica gel (gradient elution 70 to 90% EtOAc/hexanes) followed by trituration with 30% EtOAc/hexanes afforded the *N*,*O*-aminal as a white solid (490 mg, 41% yield).[7]

^1H **NMR (400 MHz, DMSO-*d*$_6$)** δ 9.18 (d, *J* = 7.8 Hz, 1H), 7.97–7.81 (m, 2H), 7.07–6.94 (m, 2H), 6.50 (d, *J* = 6.4 Hz, 1H), 5.61 (dd, *J* = 7.7, 6.2 Hz, 1H), 4.13 (qd, *J* = 7.2, 0.8 Hz, 2H), 3.81 (s, 3H), 1.20 (t, *J* = 7.1 Hz, 3H).
$^{13}C\{^1H\}$ **NMR (101 MHz, DMSO-*d*$_6$)** δ 170.1, 165.4, 161.9, 129.5, 125.7, 113.6, 71.9, 60.7, 55.4, 14.1.
IR (ATR, cm^{-1}) 3378, 3308, 2984, 2936, 1747, 1640, 1610, 1578, 1541, 1509
HRMS (ESI) calculated for $C_{12}H_{15}NNaO_5^+$ $[M+Na]^+$ 276.0842; found: 276.0834
$\mathbf{R}_f$ = 0.22 in 60% EtOAc/hexanes
mp = 117–119 °C

The title compound **1e** was prepared according to general procedure A2 using ethyl 2-(4-methoxybenzamido)-2-hydroxyacetate (**S1e**, 380 mg, 1.5 mmol). Purification by column chromatography on silica gel (gradient elution 5 to 30% EtOAc/hexanes) afforded the product as a white solid (296 mg, 71% yield). Characterization data are congruent with literature reports.[8]

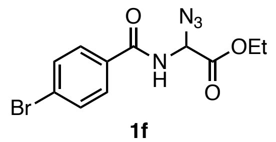

1f

ethyl 2-2-azido-2-(4-bromobenzamido)acetate (1f): Synthesized following **Route A**.

Ethyl 2-(4-bromobenzamido)-2-hydroxyacetate (**S1f**) was prepared according to general procedure A1 from 4-bromobenzamide (1.20 g, 6.0 mmol, Thermo Scientific). Trituration with 30% EtOAc/hexanes and then petroleum ether afforded the *N*,*O*-aminal as a white solid.

^{1}H NMR (400 MHz, DMSO-*d*$_6$) δ 9.45 (d, *J* = 7.8 Hz, 1H), 7.87–7.81 (m, 2H), 7.73–7.67 (m, 2H), 6.62 (d, *J* = 6.5 Hz, 1H), 5.62 (dd, *J* = 7.8, 6.5 Hz, 1H), 4.14 (q, *J* = 7.1 Hz, 2H), 1.20 (t, *J* = 7.1 Hz, 3H).
^{13}C{^{1}H} NMR (101 MHz, DMSO-*d*$_6$) δ 169.8, 165.1, 132.6, 131.4, 129.7, 125.6, 72.0, 60.8, 14.0.
IR (ATR, cm^{-1}) 3373, 3323, 3071, 2982, 2941, 2908, 1746, 1646, 1592, 1569, 1540
HRMS (ESI) calculated for $C_{11}H_{12}BrNNaO_4^+$ $[M+Na]^+$ 323.9842; found: 323.9842
R*f* = 0.17 in 40% EtOAc/hexanes
mp = 115–116°C

The title compound **1f** was prepared according to general procedure A2 using ethyl 2-(4-bromobenzamido)-2-hydroxyacetate (**S1f**, 1.21 g, 4 mmol). Purification by column chromatography on silica gel (isocratic elution with 30% EtOAc/hexanes) afforded the product as a white solid (355 mg, 27% yield). Characterization data are congruent with literature reports.[8]

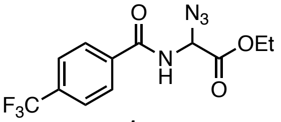

1g

ethyl 2-azido-2-(4-(trifluoromethyl)benzamido)acetate (1g): Synthesized following **Route A**.

Ethyl 2-hydroxy-2-(4-(trifluoromethyl)benzamido)acetate (**S1g**) was prepared according to general procedure A1 using 4-(trifluoromethyl)benzamide (1.32 g, 7.0 mmol, TCI). Trituration with 30% EtOAc/hexanes afforded the *N*,*O*-aminal as a white solid (835 mg, 41% yield).[7]

^{1}H NMR (400 MHz, DMSO-*d*$_6$) δ 9.62 (d, *J* = 7.8 Hz, 1H), 8.08 (d, *J* = 8.0 Hz, 2H), 7.87 (d, *J* = 8.2 Hz, 2H), 6.70 (d, *J* = 6.5 Hz, 1H), 5.65 (dd, *J* = 7.8, 6.4 Hz, 1H), 4.15 (q, *J* = 7.1 Hz, 2H), 1.20 (t, *J* = 7.1 Hz, 3H).
^{13}C{^{1}H} NMR (101 MHz, DMSO-*d*$_6$) δ 169.8, 165.0, 137.3 (q, *J* = 1.2 Hz), 131.6 (q, *J* = 31.8 Hz), 128.5, 125.4 (q, *J* = 3.9 Hz), 123.9 (q, *J* = 272.6 Hz), 72.1, 60.9, 14.0.
^{19}F NMR (376 MHz, DMSO-*d*$_6$) δ –61.27 (s, 3F).
IR (ATR, cm^{-1}) 3382, 3334, 3001, 2983, 2953, 1748, 1651, 1580, 1544, 1512
HRMS (ESI) calculated for $C_{12}H_{12}F_3NNaO_4^+$ $[M+Na]^+$ 314.0611; found: 314.0605
R*f* = 0.17 in 40% EtOAc/hexanes
mp = 124–129 °C

The title compound **1g** was prepared according to general procedure A2 using ethyl 2-hydroxy-2-(4-(trifluoromethyl)benzamido)acetate (**S1g**, 582 mg, 2.0 mmol). Purification by column chromatography on silica gel (gradient elution 5 to 20% EtOAc/hexanes) afforded the product as a white solid (488 mg, 77% yield). Characterization data are congruent with literature reports.[3]

^{1}H NMR (400 MHz, CDCl$_3$) δ 7.96 (d, *J* = 8.1 Hz, 2H), 7.76 (d, *J* = 8.2 Hz, 2H), 7.29 (br d, *J* = 7.7 Hz, 1H), 5.98 (d, *J* = 7.8 Hz, 1H), 4.38 (q, *J* = 7.2 Hz, 2H), 1.39 (t, *J* = 7.1 Hz, 3H).
^{13}C{^{1}H} NMR (101 MHz, CDCl$_3$) δ 166.9, 166.2, 135.9 (q, *J* = 1.4 Hz), 134.3 (q, *J* = 32.8 Hz), 128.0, 126.0 (q, *J* = 3.7 Hz), 123.6 (q, *J* = 272.6 Hz), 65.3, 63.6, 14.2.
^{19}F NMR (376 MHz, CDCl$_3$) δ –62.98 (s, 3F).
IR (ATR, cm^{-1}) 3324, 2988, 2956, 2105, 1743, 1648, 1583, 1523, 1499

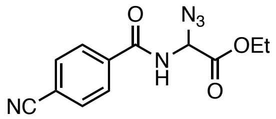

1h

ethyl 2-azido-2-(4-cyanobenzamido)acetate (1h): Synthesized following **Route A**.

Ethyl 2-(4-cyanobenzamido)-2-hydroxyacetate (**S1h**) was prepared according to general procedure A1 from 4-cyanobenzamide (730 mg, 5.0 mmol, TCI). Purification by column chromatography on silica gel (gradient elution 50 to 70% EtOAc/hexanes) followed by trituration with 30% EtOAc/hexanes afforded the *N*,*O*-aminal as a white solid (458 mg, 37% yield).

^{1}H NMR (400 MHz, DMSO-*d*$_6$) δ 9.64 (d, *J* = 7.7 Hz, 1H), 8.07–8.01 (m, 2H), 8.00–7.94 (m, 2H), 6.72 (d, *J* = 6.4 Hz, 1H), 5.64 (dd, *J* = 7.7, 6.2 Hz, 1H), 4.14 (q, *J* = 7.1 Hz, 2H), 1.20 (t, *J* = 7.1 Hz, 3H).
^{13}C{^{1}H} NMR (101 MHz, DMSO-*d*$_6$) δ 169.7, 164.7, 137.46, 132.5, 128.4, 118.3, 114.1, 72.1, 60.9, 14.0.
IR (ATR, cm^{-1}) 3357, 3302, 2986, 2233, 1744, 1682, 1647, 1609, 1541, 1500
HRMS (ESI) calculated for $C_{12}H_{12}N_2NaO_4^+$ $[M+Na]^+$ 271.0689; found: 271.0683
R$_f$ = 0.27 in 60% EtOAc/hexanes
mp = 81–83 °C

The title compound **1h** was prepared according to general procedure A2 using ethyl 2-(4-cyanobenzamido)-2-hydroxyacetate (**S1h**, 248 mg, 1.0 mmol). Purification by column chromatography on silica gel (gradient elution 10 to 40% EtOAc/hexanes) afforded the product as a white solid (200 mg, 73% yield). Characterization data are congruent with literature reports.[3]

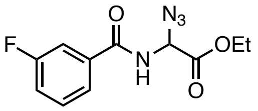

1i

ethyl 2-azido-2-(3-fluorobenzamido)acetate (1i): Synthesized following **Route A**.

Ethyl 2-(3-fluorobenzamido)-2-hydroxyacetate (**S1i**) was prepared according to general procedure A1 from 3-fluorobenzamide (696 mg, 5.0 mmol, TCI). Purification by column chromatography on silica gel (gradient elution 30 to 60% EtOAc/hexanes) afforded the *N*,*O*-aminal as a white solid (927 mg, 77% yield).

^{1}H NMR (400 MHz, DMSO-*d*$_6$) δ 9.47 (d, *J* = 7.8 Hz, 1H), 7.76 (dddd, *J* = 7.8, 1.7, 1.5, 1.0 Hz, 1H), 7.70 (ddd, *J* = 10.1, 2.6, 1.5 Hz, 1H), 7.54 (ddd, *J* = 8.0, 7.8, 5.8 Hz, 1H), 7.42 (dddd, *J* = 8.5, 8.0, 2.7, 1.0 Hz, 1H), 6.66 (d, *J* = 6.5 Hz, 1H), 5.64 (dd, *J* = 7.8, 6.4 Hz, 1H), 4.14 (q, *J* = 7.1 Hz, 2H), 1.20 (t, *J* = 7.1 Hz, 3H).
^{13}C{^{1}H} NMR (101 MHz, DMSO-*d*$_6$) δ 169.8, 164.6 (d, *J* = 2.5 Hz), 162.0 (d, *J* = 244.2 Hz), 135.8 (d, *J* = 7.2 Hz), 130.6 (d, *J* = 8.2 Hz), 123.7 (d, *J* = 2.8 Hz), 118.7 (d, *J* = 20.9 Hz), 114.3 (d, *J* = 23.1 Hz), 72.0, 60.8, 14.0.
^{19}F NMR (376 MHz, DMSO-*d*$_6$) δ –112.56 (ddd, *J* = 10.0, 8.7, 5.8 Hz, 1F).
IR (ATR, cm^{-1}) 3404, 3355, 3312, 2982, 1750, 1648, 1592, 1542, 1523
HRMS (ESI) calculated for $C_{11}H_{12}FNNaO_4^+$ $[M+Na]^+$ 264.0643; found: 264.0635
R$_f$ = 0.25 in 50% EtOAc/hexanes
mp = 94–95 °C

The title compound **1i** was prepared according to general procedure A2 using ethyl 2-(3-fluorobenzamido)-2-hydroxyacetate (**S1i**, 663 mg, 2.75 mmol). Purification by column chromatography on silica gel (gradient elution 5 to 25% EtOAc/hexanes) afforded the product as a white solid (351 mg, 48% yield). Characterization data are congruent with literature reports.[3]

^{1}H NMR (400 MHz, CDCl$_3$) δ 7.64–7.54 (m, 2H), 7.50–7.43 (m, 1H), 7.30–7.25 (m, 1H), 7.22 (d, *J* = 7.7 Hz, 1H), 5.97 (d, *J* = 7.9 Hz, 1H), 4.37 (q, *J* = 7.2 Hz, 2H), 1.39 (t, *J* = 7.2 Hz, 3H).
^{13}C{^{1}H} NMR (101 MHz, CDCl$_3$) δ 166.92, 166.12 (d, *J* = 2.5 Hz), 162.93 (d, *J* = 248.5 Hz), 134.84 (d, *J* = 6.8 Hz), 130.68 (d, *J* = 7.7 Hz), 122.89 (d, *J* = 3.3 Hz), 119.82 (d, *J* = 21.2 Hz), 114.97 (d, *J* = 23.1 Hz), 65.28, 63.53, 14.19.
^{19}F NMR (376 MHz, CDCl$_3$) δ –110.90 – –111.01 (m, 1F).
IR (ATR, cm^{-1}) 3333, 3287, 2985, 2937, 2109, 1754, 1638, 1587, 1526

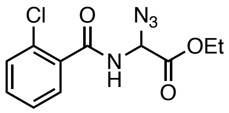

1j

ethyl 2-azido-2-(2-chlorobenzamido)acetate (1j): Synthesized following **Route A**.

Ethyl 2-(2-chlorobenzamido)-2-hydroxyacetate (**S1j**) was prepared according to general procedure A1 from 2-chlorobenzamide (778 mg, 5.0 mmol, Thermo Scientific). Purification by column chromatography on silica gel (gradient elution 30 to 100% EtOAc/hexanes) followed by trituration with 30% EtOAc/hexanes afforded the *N*,*O*-aminal as a white solid (743 mg, 58% yield).

^{1}H NMR (400 MHz, DMSO-*d*$_6$) δ 9.37 (d, *J* = 8.1 Hz, 1H), 7.52 – 7.42 (m, 2H), 7.42 – 7.37 (m, 2H), 6.70 (d, *J* = 6.7 Hz, 1H), 5.58 (dd, *J* = 8.0, 6.4 Hz, 1H), 4.14 (qd, *J* = 7.1, 1.4 Hz, 2H), 1.22 (t, *J* = 7.1 Hz, 3H).
^{13}C{^{1}H} NMR (101 MHz, DMSO-*d*$_6$) δ 169.6, 166.1, 135.9, 131.1, 130.1, 129.8, 129.0, 127.1, 71.5, 60.8, 14.1.
IR (ATR, cm^{-1}) 3368, 3273, 3062, 2998, 2944, 2902, 1746, 1650, 1592, 1571, 1537
HRMS (ESI) calculated for $C_{11}H_{12}ClNNaO_4^+$ $[M+Na]^+$ 280.0347; found: 280.0341
R*f* = 0.22 in 50% EtOAc/hexanes
mp = 98–100 °C

The title compound **1j** was prepared according to general procedure A2 using ethyl 2-(2-chlorobenzamido)-2-hydroxyacetate (**S1j**, 515 mg, 2.0 mmol). Purification by column chromatography on silica gel (gradient elution 5 to 35% EtOAc/hexanes) afforded the product as a white solid (344 mg, 61% yield).[3]

^{1}H NMR (400 MHz, CDCl$_3$) δ 7.80–7.74 (m, 1H), 7.54 (br d, *J* = 8.0 Hz, 1H), 7.47–7.39 (m, 2H), 7.37 (m, 1H), 5.97 (d, *J* = 7.9 Hz, 1H), 4.35 (q, *J* = 7.1 Hz, 2H), 1.37 (t, *J* = 7.1 Hz, 3H).
^{13}C{^{1}H} NMR (101 MHz, CDCl$_3$) δ 166.6, 166.4, 132.9, 132.5, 131.2, 131.0, 130.7, 127.4, 65.2, 63.4, 14.2.
IR (ATR, cm^{-1}) 3278, 2977, 2938, 2117, 2096, 1746, 1650, 1594, 1572, 1525
HRMS (ESI) calculated for $C_{11}H_{11}ClN_4NaO_3^+$ $[M+Na]^+$ 305.0412; found: 305.0403
R*f* = 0.22 in 20% EtOAc/hexanes
mp = 67–69 °C

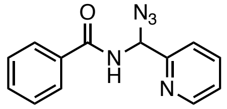

1k

***N*-(azido(pyridin-2-yl)methyl)benzamide (1k):** Synthesized following **Route B**.

N-(pyridin-2-ylmethyl)benzamide (**S1k**) was prepared according to general procedure B1 using 2-picolylamine (0.52 mL, 5.0 mmol, Sigma-Aldrich). The crude amide was obtained as a light yellow solid (1.06 g, quantitative) and used in the subsequent step without further purification. Characterization data are congruent with literature reports.[9]

The title compound **1k** was prepared according to general procedure B2 using *N*-(pyridin-2-ylmethyl)benzamide (**S1k**, 849 mg, 4.0 mmol). Purification by column chromatography on silica gel (gradient elution 10 to 40% EtOAc/hexanes) afforded the product as a light tan solid (451 mg, 45% yield).

^{1}H NMR (400 MHz, CDCl$_3$) δ 8.63 (dt, *J* = 4.9, 1.4 Hz, 1H), 8.47 (d, *J* = 7.4 Hz, 1H), 7.99–7.90 (m, 2H), 7.82 (td, *J* = 7.7, 1.8 Hz, 1H), 7.61–7.53 (m, 1H), 7.53–7.46 (m, 3H), 7.37 (ddd, *J* = 7.6, 4.9, 1.2 Hz, 1H), 6.55 (d, *J* = 7.4 Hz, 1H).
^{13}C{^{1}H} NMR (101 MHz, CDCl$_3$) δ 167.5, 153.8, 149.2, 137.9, 133.3, 132.4, 128.9, 127.5, 124.5, 122.8, 67.7.
IR (ATR, cm^{-1}) 3325, 3046, 2948, 2458, 2187, 2121, 2109, 1638, 1601, 1587, 1580, 1510
HRMS (ESI) calculated for $C_{13}H_{11}N_5NaO^+$ [M+Na]$^+$ 276.0856; found: 276.0848
R$_f$ = 0.20 in 30% EtOAc/hexanes
mp = 55–56 °C

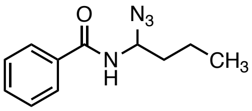

1l

***N*-(1-azidobutyl)benzamide** (**1l**) was synthesized following **Route B** in accordance with a reported literature procedure. Spectroscopic data are in agreement with those previously reported.[3]

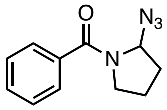

1m

(2-azidopyrrolidin-1-yl)(phenyl)methanone (**1m**) was synthesized following **Route B** in accordance with a reported literature procedure. Spectroscopic data are in agreement with those previously reported.[3]

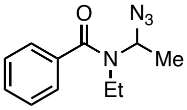

1n

***N*-(1-azidoethyl)-*N*-ethylbenzamide** (**1n**) was synthesized following **Route B** in accordance with a reported literature procedure. Spectroscopic data are in agreement with those previously reported.[3]

III. General Procedure for Iminyl Radical Cyclization

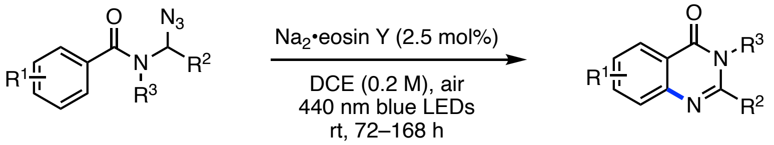

An oven-dried 25 mL clear borosilicate glass round-bottom flask equipped with a PTFE-coated stir bar was charged with the azide substrate (0.50 mmol, 1.0 equiv), eosin Y disodium salt (8.6 mg, 0.0125 mmol, 0.025 equiv), and anhydrous 1,2-dichloroethane (2.5 mL). The round-bottom flask was then fitted with a vacuum/gas adapter (14/20 joint, bent 90°), which was left open to the ambient atmosphere, and irradiated with a 440 nm Kessil LED lamp (100% intensity, 2 cm horizontal distance with cooling fan as depicted in Figure S1a). Upon completion, the reaction mixture was concentrated under reduced pressure, and the residue was purified by column chromatography on silica gel to afford the desired product.

Note: Unless otherwise noted, all photoreactions were carried out using a single 440 nm Kessil LED lamp and stirred for the indicated time, furnishing the yields reported in Table 2 and detailed below. However, shorter reaction times may be achieved by using two Kessil lamps. For example, complete consumption of substrate **1a** was observed after irradiating 18 h with two lamps (Figure S1b), compared to 72 h with a single lamp (Figure S1a).

(a) (b)

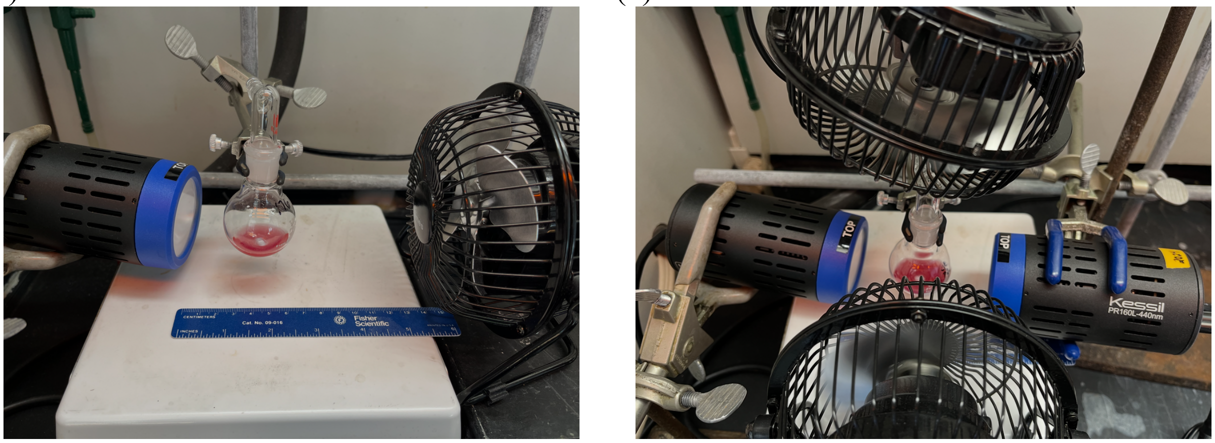

Figure S1. (a) Single light and cooling fan arrangement. (b) Dual light and cooling fan arrangement for shorter reaction time and 1 mmol scale up.

Synthesis and Characterization of Products

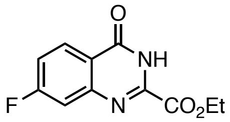

2a

ethyl 7-fluoro-4-oxo-3,4-dihydroquinazoline-2-carboxylate (2a)

Prepared according to the general procedure using ethyl 2-azido-2-(4-fluorobenzamido)acetate (**1a**) (133.1 mg, 0.50 mmol) and irradiating for 72 h. Purification by column chromatography on silica gel (gradient elution 5 to 30% EtOAc/hexanes) afforded the title compound as a white solid (run 1 = 92.1 mg, 78% yield; run 2 = 96.0 mg, 81% yield). Characterization data are congruent with literature reports.[3]

^{1}H NMR (400 MHz, $CDCl_3$) δ 9.98 (br s, 1H), 8.37 (dd, *J* = 8.9, 6.0 Hz, 1H), 7.62 (dd, *J* = 9.3, 2.5 Hz, 1H), 7.34 (ddd, *J* = 8.8, 8.0, 2.5 Hz, 1H), 4.58 (q, *J* = 7.1 Hz, 2H), 1.50 (t, *J* = 7.1 Hz, 3H).
^{13}C{^{1}H} NMR (101 MHz, $CDCl_3$) δ 166.8 (d, *J* = 256.2 Hz), 160.5, 160.3, 150.0 (d, *J* = 13.0 Hz), 142.8, 129.6 (d, *J* = 10.5 Hz), 119.9 (d, *J* = 2.3 Hz), 118.1 (d, *J* = 23.3 Hz), 114.9 (d, *J* = 22.2 Hz), 64.6, 14.3.
^{19}F NMR (376 MHz, $CDCl_3$) δ –101.12 – –101.23 (m, 1F).
IR (ATR, cm^{-1}) 3463, 3187, 3073, 2940, 1741, 1679, 1627, 1606, 1501

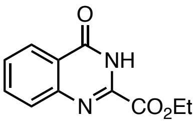

2b

ethyl 4-oxo-3,4-dihydroquinazoline-2-carboxylate (2b)

Prepared according to the general procedure using ethyl 2-azido-2-benzamidoacetate (**1b**) (124.1 mg, 0.50 mmol) and irradiating for 72 h. Purification by column chromatography on silica gel (gradient elution 3 to 10% MeCN/DCM) afforded the title compound as a white solid (run 1 = 99.5 mg, 91% yield; run 2 = 98.6 mg, 90% yield). Characterization data are congruent with literature reports.[10]

^{1}H NMR (400 MHz, $CDCl_3$) δ 9.93 (br s, 1H), 8.36 (dd, *J* = 8.0, 1.5 Hz, 1H), 7.97 (d, *J* = 8.0 Hz, 1H), 7.85 (ddd, *J* = 8.3, 7.2, 1.5 Hz, 1H), 7.67–7.60 (m, 1H), 4.58 (q, *J* = 7.1 Hz, 2H), 1.50 (t, *J* = 7.1 Hz, 3H).
^{13}C{^{1}H} NMR (101 MHz, $CDCl_3$) δ 161.0, 160.7, 147.7, 141.7, 135.2, 129.5, 129.4, 127.0, 123.3, 64.4, 14.3.
IR (ATR, cm^{-1}) 3182, 3070, 2994, 2966, 2923, 1737, 1726, 1674, 1603, 1494

Scale-up of 2b

An oven-dried 25 mL round-bottom flask equipped with a PTFE-coated stir bar was charged with ethyl 2-azido-2-benzamidoacetate (**1b**) (248.2 mg, 1.00 mmol), eosin Y disodium salt (17.3 mg, 0.025 mmol), and anhydrous 1,2-dichloroethane (5.0 mL). The round-bottom flask was then fitted with a vacuum/gas adapter (14/20 joint, bent 90 °), which was left open to the ambient atmosphere,

and irradiated with two Kessil PR160L-440 LED lamps (both 100% intensity, 3 cm horizontal distance from flask, 180° apart with two cooling fans as depicted in Figure S1b). After 72 h, the reaction mixture was concentrated under reduced pressure, and the residue was purified by column chromatography on silica gel (gradient elution 3 to 10% MeCN/DCM) to afford the title compound as a white solid (171.4 mg, 79% yield).

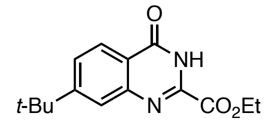

2d

ethyl 7-(tert-butyl)-4-oxo-3,4-dihydroquinazoline-2-carboxylate (2d)

Prepared according to the general procedure using ethyl 2-azido-2-(4-(*tert*-butyl)benzamido)acetate (**1d**) (152.2 mg, 0.50 mmol) and irradiating for 72 h. Purification by column chromatography on silica gel (gradient elution 10 to 30% EtOAc/hexanes) afforded the title compound as a white crystalline solid (run 1 = 92.6 mg, 68% yield; run 2 = 95.8 mg, 70% yield).

^{1}H NMR (400 MHz, $CDCl_3$) δ 9.91 (br s, 1H), 8.27 (d, J = 8.5 Hz, 1H), 7.97 (d, J = 1.8 Hz, 1H), 7.69 (dd, J = 8.4, 1.9 Hz, 1H), 4.58 (q, J = 7.1 Hz, 2H), 1.50 (t, J = 7.1 Hz, 3H), 1.40 (s, 9H).
^{13}C{^{1}H} NMR (101 MHz, $CDCl_3$) δ 160.9, 160.8, 159.5, 147.7, 141.7, 127.5, 126.6, 125.9, 120.7, 64.3, 35.7, 31.2, 14.3.
IR (ATR, cm^{-1}) 3197, 3159, 3097, 2965, 2905, 2872, 1727, 1670, 1608, 1477
HRMS (ESI) calculated for $C_{15}H_{19}N_2O_3^+$ $[M+H]^+$ 275.1390; found: 275.1386
R_f = 0.29 in 40% EtOAc/hexanes
mp = 167–169°C

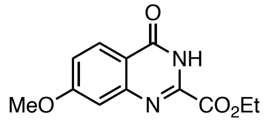

2e

ethyl 7-methoxy-4-oxo-3,4-dihydroquinazoline-2-carboxylate (2e)

Prepared according to the general procedure using ethyl 2-azido-2-(4-methoxybenzamido)acetate (**1e**) (139.1 mg, 0.50 mmol) and irradiating for 168 h. Purification by column chromatography on silica gel (gradient elution 10 to 70% EtOAc/hexanes) afforded the title compound as a white solid (run 1 = 89.1 mg, 72% yield; run 2 = 110.5, 89% yield; run 3 = 94.7 mg, 76% yield). Characterization data are congruent with literature reports.[3]

^{1}H NMR (400 MHz, $CDCl_3$) δ 10.23 (br s, 1H), 8.24 (d, J = 8.9 Hz, 1H), 7.35 (d, J = 2.5 Hz, 1H), 7.17 (dd, J = 8.9, 2.5 Hz, 1H), 4.57 (q, J = 7.1 Hz, 2H), 3.92 (s, 3H), 1.49 (t, J = 7.1 Hz, 3H).
^{13}C{^{1}H} NMR (101 MHz, $CDCl_3$) δ 165.2, 160.7, 160.5, 150.0, 142.3, 128.3, 119.4, 116.6, 110.2, 64.3, 56.0, 14.3.
IR (ATR, cm^{-1}) 3147, 3051, 2986, 2092, 1752, 1734, 1678, 1657, 1607, 1567

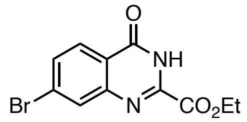

2f

ethyl 7-bromo-4-oxo-3,4-dihydroquinazoline-2-carboxylate (2f)

Prepared according to the general procedure using ethyl 2-azido-2-(4-bromobenzamido)acetate (**1f**) (163.6 mg, 0.50 mmol) and irradiating for 72 h. Purification by column chromatography on silica gel (gradient elution 10 to 20% EtOAc/hexanes then 15 to 30% acetone/hexanes) afforded the title compound as a white solid (run 1 = 103.0 mg, 69% yield; run 2 = 102.4 mg, 69% yield). Characterization data are congruent with literature reports.[3]

^{1}H NMR (400 MHz, CDCl$_3$) 10.04 (br s, 1H), 8.20 (d, *J* = 8.5 Hz, 1H), 8.15 (d, *J* = 1.8 Hz, 1H), 7.73 (dd, *J* = 8.5, 1.9 Hz, 1H), 4.58 (q, *J* = 7.1 Hz, 2H), 1.50 (t, *J* = 7.1 Hz, 3H).
^{13}C{^{1}H} NMR (101 MHz, CDCl$_3$) δ 160.7, 160.4, 148.7, 142.8, 132.7, 132.1, 130.0, 128.3, 122.0, 64.5, 14.3.
IR (ATR, cm^{-1}) 3262, 3173, 3066, 2909, 1757, 1738, 1678, 1610, 1594, 1556

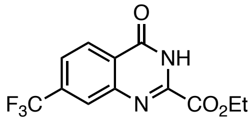

2g

ethyl 4-oxo-7-(trifluoromethyl)-3,4-dihydroquinazoline-2-carboxylate (2g)

Prepared according to the general procedure using ethyl 2-azido-2-(4-(trifluoromethyl)benzamido)acetate (**1g**) (158.1 mg, 0.50 mmol) and irradiating for 72 h. Purification by column chromatography on silica gel (gradient elution 5 to 30% EtOAc/hexanes) afforded the title compound as a white solid (run 1 = 81.4 mg, 57% yield; run 2 = 124.9 mg, 87% yield).[3]

^{1}H NMR (400 MHz, CDCl$_3$) δ 10.82 (br s, 1H), 8.47 (d *J* = 8.4 Hz, 1H), 8.24 (s, 1H), 7.81 (dd, *J* = 8.6, 1.7 Hz, 1H), 4.59 (q, *J* = 7.1 Hz, 2H), 1.50 (t, *J* = 7.2 Hz, 3H).
^{13}C{^{1}H} NMR (101 MHz, CDCl$_3$) δ 160.5, 160.4, 147.8, 143.0, 136.8 (q, *J* = 33.3 Hz), 128.1, 126.8 (q, *J* = 4.1 Hz), 125.6, 125.4 (q, *J* = 3.4 Hz), 123.2 (q, J = 273.1 Hz), 64.6, 14.3.
^{19}F NMR (376 MHz, CDCl$_3$) δ –63.23 (s, 3F).
IR (ATR, cm^{-1}) 3194, 3158, 3094, 2984, 1758, 1664, 1611, 1569, 1499
HRMS (ESI) calculated for $C_{12}H_{10}F_3N_2O_3^+$ [M+H]$^+$ 287.0638; found: 287.0640
R*f* = 0.27 in 40% EtOAc/hexanes
mp = 151–153 °C

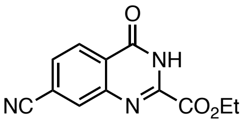

2h

ethyl 7-cyano-4-oxo-3,4-dihydroquinazoline-2-carboxylate (2h)

Prepared according to the general procedure using ethyl 2-azido-2-(4-cyanobenzamido)acetate (**1h**) (136.6 mg, 0.50 mmol) and irradiating for 168 h. Purification by column chromatography on silica gel (gradient elution 10 to 30% acetone/hexanes) afforded the title compound as an off-white solid (run 1 = 66.0 mg, 54% yield; run 2 = 61.5 mg, 51% yield).[3]

^{1}H NMR (400 MHz, $CDCl_3$) δ 10.16 (br s, 1H), 8.45 (d, J = 8.2 Hz, 1H), 8.26 (d, J = 1.5 Hz, 1H), 7.83 (dd, J = 8.2, 1.5 Hz, 1H), 4.59 (q, J = 7.1 Hz, 2H), 1.51 (t, J = 7.1 Hz, 3H).
^{13}C{^{1}H} NMR (101 MHz, $CDCl_3$) δ 160.2, 160.1, 147.8, 143.3, 133.8, 131.2, 128.3, 126.2, 118.7, 117.3, 64.8, 14.2.
IR (ATR, cm^{-1}) 3147, 3035, 2924, 2888, 2235, 1749, 1674, 1609, 1557, 1493
HRMS (ESI) calculated for $C_{12}H_9N_3NaO_3^+$ [M+Na]$^+$ 266.0536; found: 266.0529
R$_f$ = 0.27 in 60% EtOAc/hexanes
mp = 211–213 °C

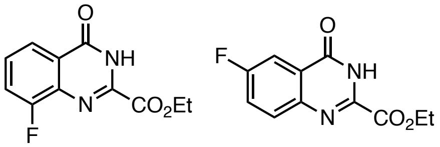

2i-8 **2i-6**

ethyl 8-fluoro-4-oxo-3,4-dihydroquinazoline-2-carboxylate (2i-8) and ethyl 6-fluoro-4-oxo-3,4-dihydroquinazoline-2-carboxylate (2i-6)

Prepared according to the general procedure using ethyl 2-azido-2-(3-fluorobenzamido)acetate (**1i**) (133.1 mg, 0.50 mmol) and irradiating for 72 h. Purification by column chromatography on silica gel (gradient elution 5 to 100% EtOAc/hexanes) afforded the title compounds as a white solid. The 8- and 6-substituted regioisomers were not separable by column chromatography and were isolated as a 1.7:1 mixture, favoring **2i-8** (run 1 = 83.6 mg, 71% yield, 1.7:1 r.r.; run 2 = 108.7 mg, 92% yield, 1.7:1 r.r.).[3]

Mixture ^{1}H NMR (400 MHz, $CDCl_3$) δ 10.04 (br s, 2H), 8.18–8.11 (m, 1H), 8.02–7.96 (m, 1H), 7.63 – 7.53 (m, 4H), 4.58 (two overlapping quartets, 4H), 1.50 (app t, J = 7.1 Hz, 6H).
Mixture ^{13}C{^{1}H, ^{19}F} NMR (101 MHz, $CDCl_3$) δ 162.5, 160.6, 160.6, 160.6, 160.3, 158.1, 144.3, 142.1, 141.3, 137.2, 131.9, 129.7, 125.0, 124.9, 123.7, 122.5, 120.9, 112.2, 64.4, 64.3, 14.3, 14.2.
Mixture ^{19}F NMR (376 MHz, $CDCl_3$) [1.7:1 mixture of regioisomers] **2i-6** δ –107.97 (ddd, J = 8.0, 8.0, 4.8 Hz, 1F); **2i-8** δ –120.45 – –120.56 (m, 1F).
Mixture IR (ATR, cm^{-1}) 3175, 3073, 3003, 2906, 1750, 1668, 1617, 1606, 1573, 1470
Mixture HRMS (ESI) calculated for $C_{11}H_9FN_2NaO_3^+$ [M+Na]$^+$ 259.0489; found: 259.0481
Mixture R$_f$ = 0.18 in 40% EtOAc/hexanes
Mixture mp = 193–195 °C (dec)

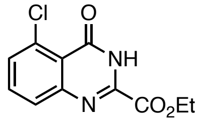

2j

ethyl 5-chloro-4-oxo-3,4-dihydroquinazoline-2-carboxylate (2j)

Prepared according to the general procedure using ethyl 2-azido-2-(2-chlorobenzamido)acetate (**1j**) (141.3 mg, 0.50 mmol) and irradiating for 168 h. Purification by column chromatography on silica gel (isocratic elution using 30% EtOAc/hexanes) afforded the title compound as a white solid (run 1 = 12.8 mg, 10% yield; run 2 = 14.6 mg, 12% yield).

^{1}H NMR (400 MHz, $CDCl_3$) δ 9.81 (br s, 1H), 7.86 (dd, *J* = 8.1, 1.2 Hz, 1H), 7.70 (t, *J* = 8.0 Hz, 1H), 7.60 (dd, *J* = 7.9, 1.2 Hz, 1H), 4.57 (q, *J* = 7.1 Hz, 2H), 1.49 (t, *J* = 7.2 Hz, 3H).
^{13}C{^{1}H} NMR (101 MHz, $CDCl_3$) δ 160.3, 159.2, 150.1, 142.3, 134.9, 134.5, 132.1, 128.6, 120.5, 64.5, 14.3.
IR (ATR, cm^{-1}) 3178, 3111, 3057, 2984, 2934, 1750, 1671, 1619, 1593, 1553
HRMS (ESI) calculated for $C_{11}H_9ClN_2NaO_3^+$ $[M+Na]^+$ 275.0194; found: 275.0190
R_f = 0.20 in 40% EtOAc/hexanes
mp = 171–173 °C (discoloration beginning at 135 °C)

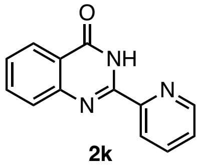

2k

2-(pyridin-2-yl)quinazolin-4(3*H*)-one (2k)

Prepared according to the general procedure using *N*-(azido(pyridin-2-yl)methyl)benzamide (**1k**) (126.6 mg, 0.50 mmol) and irradiating for 168 h. Purification by column chromatography on silica gel (isocratic elution using 3% $MeCN/CH_2Cl_2$) afforded the title compound as a white solid (run 1 = 61.0 mg, 55% yield; run 2 = 39.8 mg, 36% yield). Characterization data are congruent with literature reports.[11]

^{1}H NMR (400 MHz, $CDCl_3$) δ 10.96 (br s, 1H), 8.68 (ddd, *J* = 4.8, 1.7, 0.9 Hz, 1H), 8.60 (dt, *J* = 8.0, 1.1 Hz, 1H), 8.36 (ddd, *J* = 8.0, 1.5, 0.7 Hz, 1H), 7.93 (td, *J* = 7.8, 1.7 Hz, 1H), 7.86–7.76 (m, 2H), 7.56–7.46 (m, 2H).
^{13}C{^{1}H} NMR (101 MHz, $CDCl_3$) δ 161.5, 149.3, 149.1, 148.9, 148.5, 137.7, 134.7, 128.2, 127.4, 126.9, 126.4, 122.6, 122.1.
IR (ATR, cm^{-1}) 3252, 3094, 3069, 3006, 1671, 1601, 1564, 1487, 1470

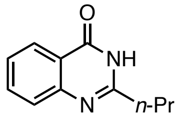

2l

2-propylquinazolin-4(3*H*)-one (2l)

Prepared according to the general procedure using *N*-(1-azidobutyl)benzamide (**1l**) (109.1 mg, 0.50 mmol) and irradiating for 168 h. Purification by column chromatography on silica gel (gradient elution 20 to 40% EtOAc/hexanes) afforded the title compound as a white solid (run 1 = 12.0 mg, 13% yield; run 2 = 8.1 mg, 9% yield). Characterization data are congruent with literature reports.[12]

^{1}H NMR (400 MHz, DMSO-d_6) δ 12.17 (br s, 1H), 8.07 (dd, *J* = 7.9, 1.6 Hz, 1H), 7.77 (ddd, *J* = 8.6, 7.1, 1.6 Hz, 1H), 7.62–7.56 (m, 1H), 7.49–7.42 (m, 1H), 2.57 (t, *J* = 7.5 Hz, 2H), 1.74 (sext, *J* = 7.4 Hz, 2H), 0.93 (t, *J* = 7.4 Hz, 3H).
^{13}C{^{1}H} NMR (101 MHz, DMSO-d_6) δ 161.8, 157.3, 149.0, 134.3, 126.8, 125.9, 125.7, 120.8, 36.4, 20.2, 13.5.
IR (ATR, cm^{-1}) 3168, 3033, 2966, 2921, 2875, 1673, 1621, 1565, 1505, 1471

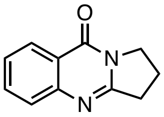

2m

2,3-dihydropyrrolo[2,1-*b*]quinazolin-9(1*H*)-one (deoxyvasicinone, 2m)

Prepared according to the general procedure using (2-azidopyrrolidin-1-yl)(phenyl)methanone (**1m**) (108.1 mg, 0.50 mmol) and irradiating for 168 h. Purification by column chromatography on silica gel (gradient elution 0 to 30% acetone/hexanes) followed by preparative thin layer chromatography (75% EtOAc/hexanes) afforded the title compound as a white solid (run 1 = 8.1 mg, 9% yield; run 2 = 8.0 mg, 9% yield). Characterization data are congruent with literature reports.[13]

^{1}H NMR (400 MHz, CDCl$_3$) δ 8.29 (dd, *J* = 8.2, 1.3 Hz, 1H), 7.74 (ddd, *J* = 8.4, 6.9, 1.5 Hz, 1H), 7.71–7.66 (m, 1H), 7.46 (ddd, *J* = 8.1, 6.9, 1.3 Hz, 1H), 4.23 (t, *J* = 7.4 Hz, 2H), 3.23 (t, *J* = 7.9 Hz, 2H), 2.31 (app p, *J* = 7.7 Hz, 2H).
^{13}C{^{1}H} NMR (101 MHz, CDCl$_3$) δ 160.8, 160.1, 147.9, 134.6, 126.8, 126.7, 126.2, 120.4, 46.9, 32.5, 19.6.
IR (ATR, cm^{-1}) 3473, 3238, 2921, 2852, 1650, 1612, 1562, 1469

Other substrates examined:

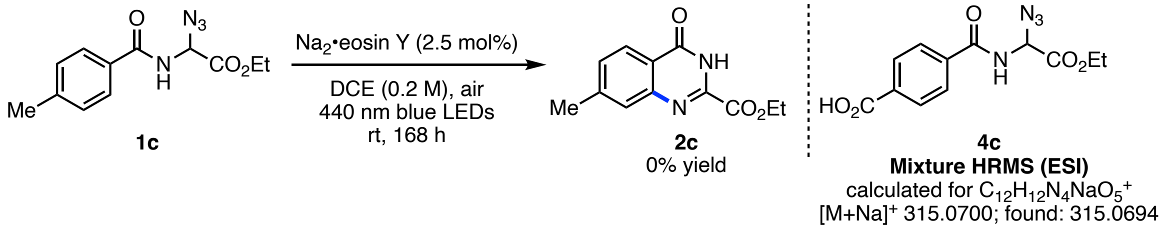

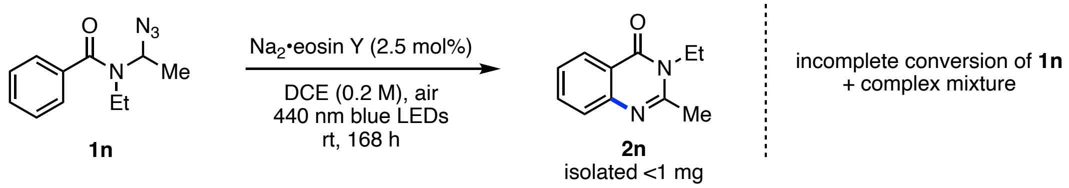

IV. Additional Experiments

Optimization Studies

General Procedure for Reaction Optimization (Tables 1, S1–S4)
To an oven-dried, one-dram clear borosilicate glass vial equipped with a PTFE-coated stir bar was added substrate **1a** (10.6 mg, 0.040 mmol, 1.0 equiv) and photocatalyst (0.002 mmol, 0.050 equiv, or amount noted). If applicable, solid additive was then added to vial (0.020 mmol, 0.5 equiv, or amount noted); liquid additive was added via microsyringe after the addition of reaction solvent. The vial was then sealed with a plastic cap lined with a Teflon septum and contents were kept under ambient atmosphere using an open 21-gauge vent needle inserted into septum. Anhydrous solvent was added to the vial (0.2 mL) via syringe. The reaction mixture was irradiated with a 440 nm Kessil LED lamp (100% intensity, 2 cm horizontal distance with cooling fan, or wavelength and intensity noted) for 24 h with stirring. Upon completion, the reaction mixture was concentrated under reduced pressure, and the crude residue was analyzed by ^{19}F NMR spectroscopy with 1,4-dibromo-2,5-difluorobenzene as an external standard.

Reactions conducted under a nitrogen atmosphere were sealed with a plastic cap lined with a Teflon septum, evacuated and backfilled with nitrogen 3×, and sealed with Parafilm prior to irradiation.

Note: Oxidation product **3a** (ethyl 2-(4-fluorobenzamido)-2-oxoacetate) was observed and characterized as the major side product when using eosin Y or Na_2·eosin Y as the photocatalyst.

Table S1. Evaluation of Photocatalysts

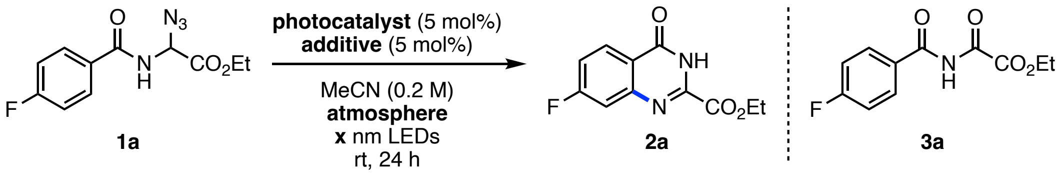

photocatalyst	atmosphere	additive	light source	yield **2a** (%)
TBADT	sealed under N_2	–	390 nm Kessil[a]	0
TBADT	sealed under N_2	–	390 nm Kessil	0
TBADT	sealed under N_2	COPC	390 nm Kessil	0
TBADT	sealed under N_2	COBF	390 nm Kessil	trace
benzophenone	sealed under N_2	–	390 nm Kessil[a]	4
benzophenone	ambient	–	390 nm Kessil[a]	4
xanthone	sealed under N_2	–	390 nm Kessil[a]	2
anthraquinone	sealed under N_2	–	390 nm Kessil[a]	5
rhodamine 6G	sealed under N_2	–	440 nm Kessil	trace
rhodamine 6G	ambient	–	440 nm Kessil	2
eosin Y	sealed under N_2	–	440 nm Kessil	3
eosin Y	ambient	–	440 nm Kessil	26

[a]LED lamp set to 25% intensity. TBADT = tetra-*n*-butylammonium decatungstate, COPC = chloro(pyridine)bis(dimethylglyoximato)cobalt(III), COBF = *N*,*N*',*N*,*N*'-(tetrafluorodiborato)bis[μ-(2,3-butanedionedioximato)]cobalt(II) dihydrate

Table S2. Evaluation of Reaction Solvent

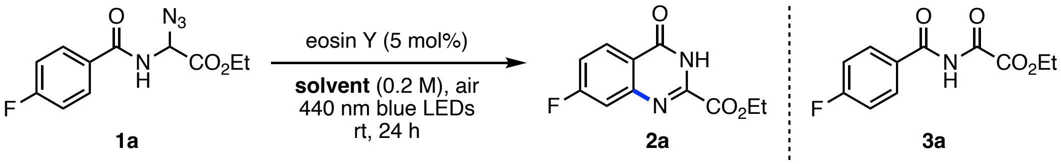

solvent	yield **2a** (%)
MeCN	26
DCM	45
acetone	15
DCE	63
methanol	0

Table S3: Evaluation of Additives

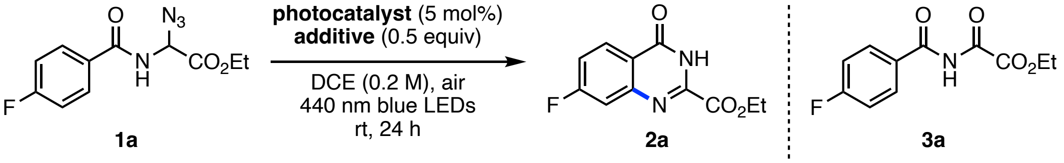

photocatalyst	additive	yield **2a** (%)
eosin Y	–	63
eosin Y	triflic acid	20
eosin Y	*p*-TsOH·H_2O	44
eosin Y	TFA	64
eosin Y	NaOH	39
eosin Y	Na_2CO_3	52
eosin Y	K_2HPO_4	42
eosin Y	$NaH_2PO_4 \cdot H_2O$	71
eosin Y	TMEDA	6
eosin Y	pyridine	1
eosin Y	DBU	trace
Na_2·eosin Y	–	71
Na_2·eosin Y	TFA	73
Na_2·eosin Y	acetic acid	73
Na_2·eosin Y	$NaH_2PO_4 \cdot H_2O$	72

TFA = trifluoroacetic acid, TMEDA = *N*,*N*,*N*′,*N*′-tetramethylethylenediamine, DBU = 1,8-diazabicyclo[5.4.0]undec-7-ene

Table S4: Evaluation of Oxidants

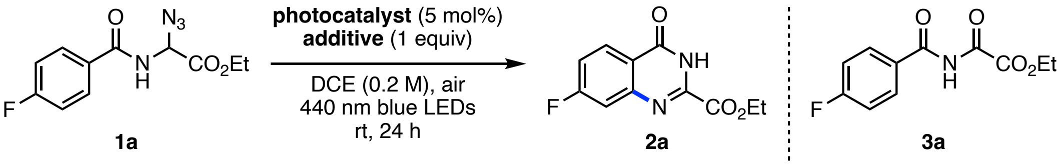

photocatalyst	atmosphere	additive	yield **2a** (%)
eosin Y	ambient	–	63
eosin Y	sealed under N_2	*t*-BuOOH	7
eosin Y	sealed under N_2	$K_2S_2O_8$	10
eosin Y	sealed under N_2	$PhI(OAc)_2$	7
Na_2·eosin Y	ambient	–	71
Na_2·eosin Y	O_2 balloon	–	62
Na_2·eosin Y[a]	sealed under N_2	–	3
Na_2·eosin Y[a]	sealed under N_2	KO_2	0
Na_2·eosin Y[a]	sealed under N_2	*t*-BuOOH	8
Na_2·eosin Y[a]	sealed under N_2	$K_2S_2O_8$	7
Na_2·eosin Y[a]	sealed under N_2	$PhI(OAc)_2$	8
Na_2·eosin Y[a]	sealed under N_2	$AgNO_3$	0

[a]Reactions were conducted using 2.5 mol% Na_2·eosin Y.

We thank Prof. E. William Hamilton for providing oxygen for control experiments.

Reaction Profile and Photodegradation Experiments

On the 0.04 mmol scale, Na$_2$·eosin Y and quinazolinone product **2a** are partially soluble in 1,2-dichloroethane. Therefore, each data point of the reaction profile represents the average of at least two independent experiments, each of which was terminated at the time indicated, followed by analysis the reaction mixture. Figure 3A from the manuscript is included below as Figure S2 with standard deviations represented as error bars.

General Procedure for Reaction Profile

To an oven-dried, one-dram clear borosilicate glass vial was added eosin Y disodium salt (0.7 mg, 0.001 mmol, 0.025 equiv) as a stock solution in ethanol. Solvent was removed by evaporation before substrate **1a** (10.6 mg, 0.040 mmol, 1.0 equiv) was added. The vial was then sealed with a plastic cap lined with a Teflon septum and contents were kept under ambient atmosphere using an open 21-gauge vent needle inserted into septum. Anhydrous 1,2-dichloroethane (0.2 mL) was added to the vial via syringe. The reaction mixture was irradiated with a 440 nm Kessil LED lamp (100% intensity, 2 cm horizontal distance with cooling fan) for the given time with stirring. After the allotted time, irradiation was discontinued, and the reaction mixture was concentrated under reduced pressure. The crude residue was analyzed by ^{19}F NMR spectroscopy with 1,4-dibromo-2,5-difluorobenzene as an external standard.

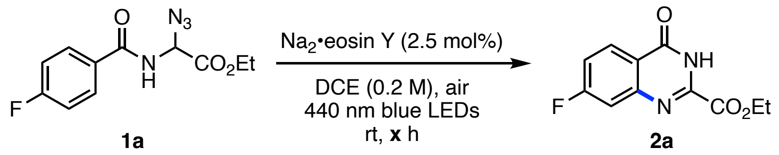

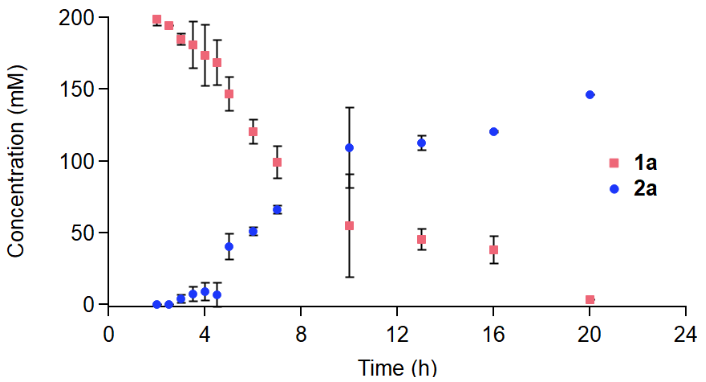

Figure S2. Reaction profile of the iminyl radical cyclization.

An aliquot of the NMR sample was then diluted with DMSO (6.67×10^{-5} M Na$_2$·eosin Y after dilution), and the resulting solution was added to a 1 cm path length quartz cuvette and analyzed via UV–vis spectroscopy. A representative UV–vis absorption spectrum at various time points is shown in Figure S3.

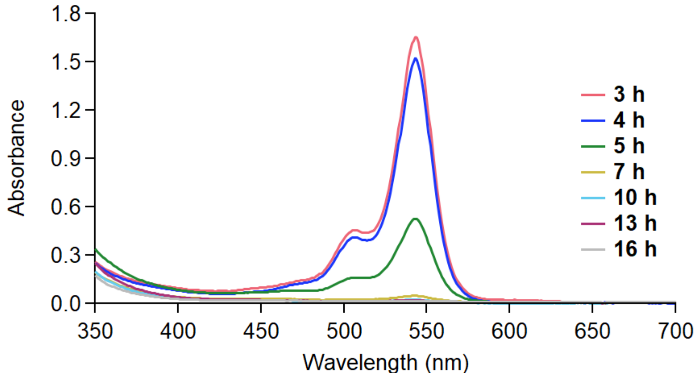

Figure S3. UV–vis analysis of iminyl radical cyclization.

Preliminary Mechanistic Investigations

General Procedure for Radical and Reactive Oxygen Scavenging Experiments

To an oven-dried, one-dram clear borosilicate glass vial equipped with a PTFE-coated stir bar was added substrate **1a** (10.6 mg, 0.040 mmol, 1.0 equiv), eosin Y disodium salt (0.7 mg, 0.001 mmol, 0.025 equiv), and scavenger additive (0.040 mmol, 1.0 equiv). The vial was then sealed with a plastic cap lined with a Teflon septum and contents were kept under ambient atmosphere using an open 21-gauge vent needle inserted into septum. Anhydrous 1,2-dichloroethane (0.2 mL) was added to the vial via syringe. The reaction mixture was irradiated with a 440 nm Kessil LED lamp (100% intensity, 2 cm horizontal distance with cooling fan) for 24 h with stirring. Upon completion, the reaction mixture was concentrated under reduced pressure, and the crude residue was analyzed by ^{19}F NMR spectroscopy with 1,4-dibromo-2,5-difluorobenzene as an external standard.

Table S5: Evaluation of Quenchers

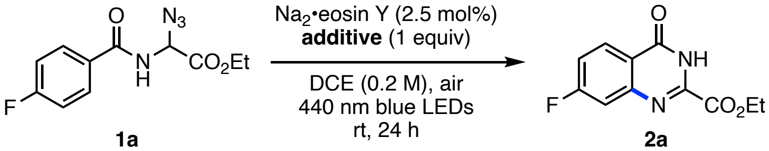

additive	yield **2a** (%)
TEMPO	0
BHT	trace
DABCO	trace
benzoquinone	1

TEMPO = 2,2,6,6-tetramethylpiperidine 1-oxyl, BHT = 2,6-di-*tert*-butyl-4-methylphenol, DABCO = 1,4-diazabicyclo[2.2.2]octane

V. NMR Spectra

^{1}H NMR (400 MHz, DMSO-d_6) of ethyl 2-(4-fluorobenzamido)-2-hydroxyacetate (**S1a**)

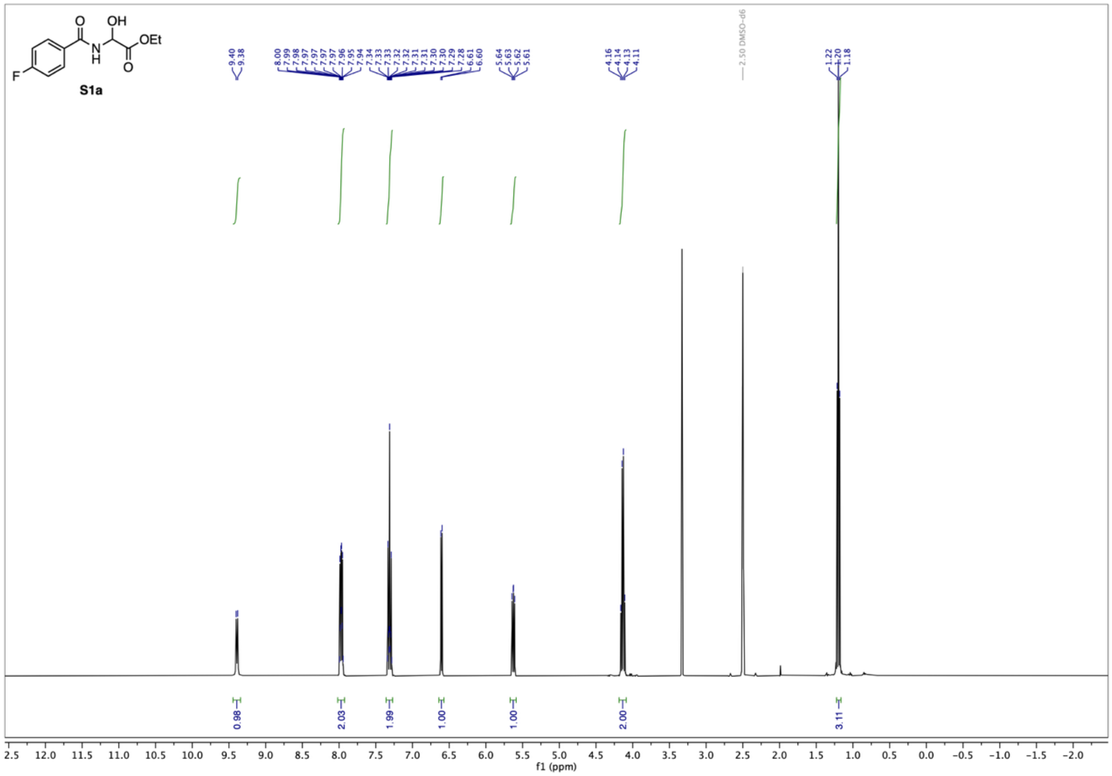

^{13}C{^{1}H} NMR (101 MHz, DMSO-d_6) of ethyl 2-(4-fluorobenzamido)-2-hydroxyacetate (**S1a**)

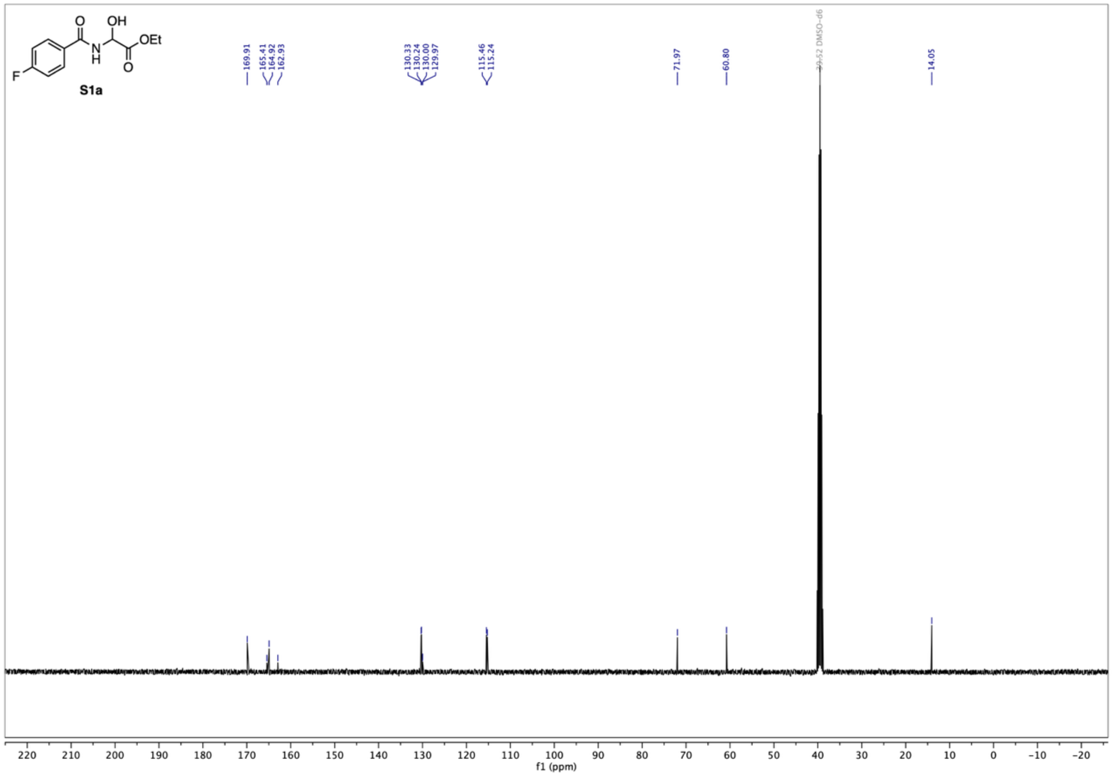

^{19}F NMR (376 MHz, DMSO-d_6) of ethyl 2-(4-fluorobenzamido)-2-hydroxyacetate (**S1a**)

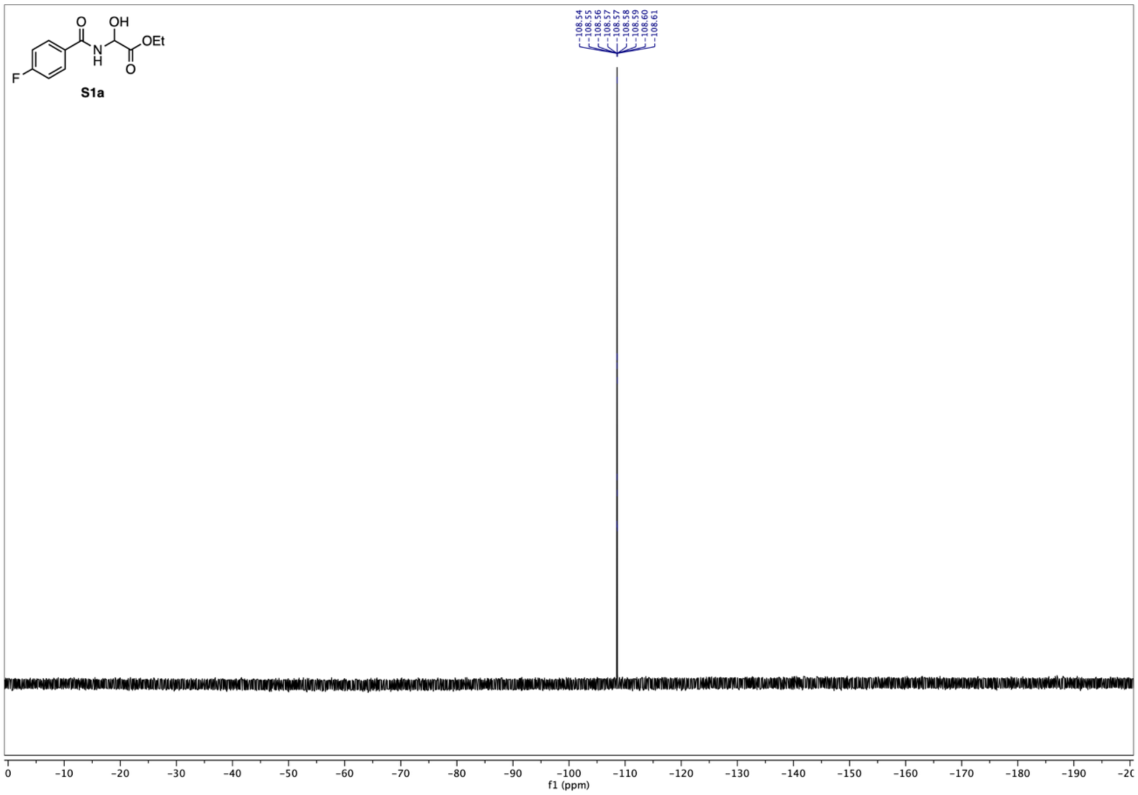

^{1}H NMR (400 MHz, $CDCl_3$) of ethyl 2-azido-2-(4-fluorobenzamido)acetate (**1a**)

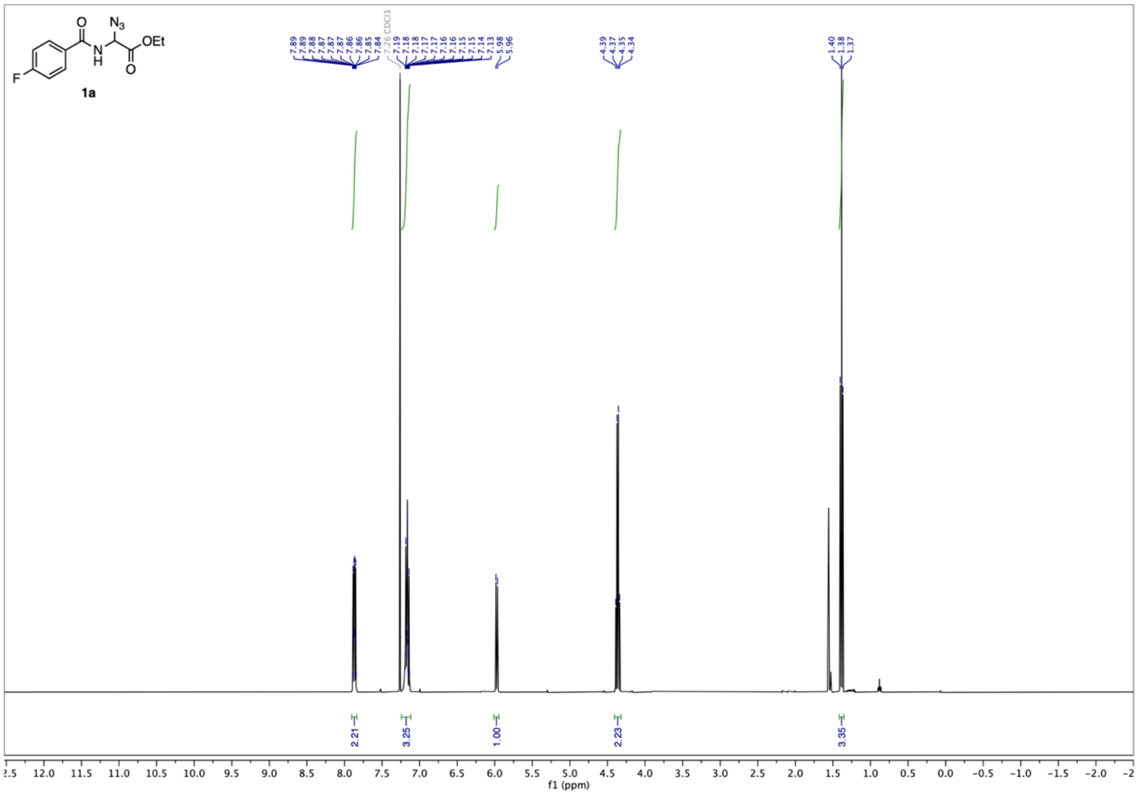

$^{13}C\{^{1}H\}$ NMR (101 MHz, $CDCl_3$) of ethyl 2-azido-2-(4-fluorobenzamido)acetate (**1a**)

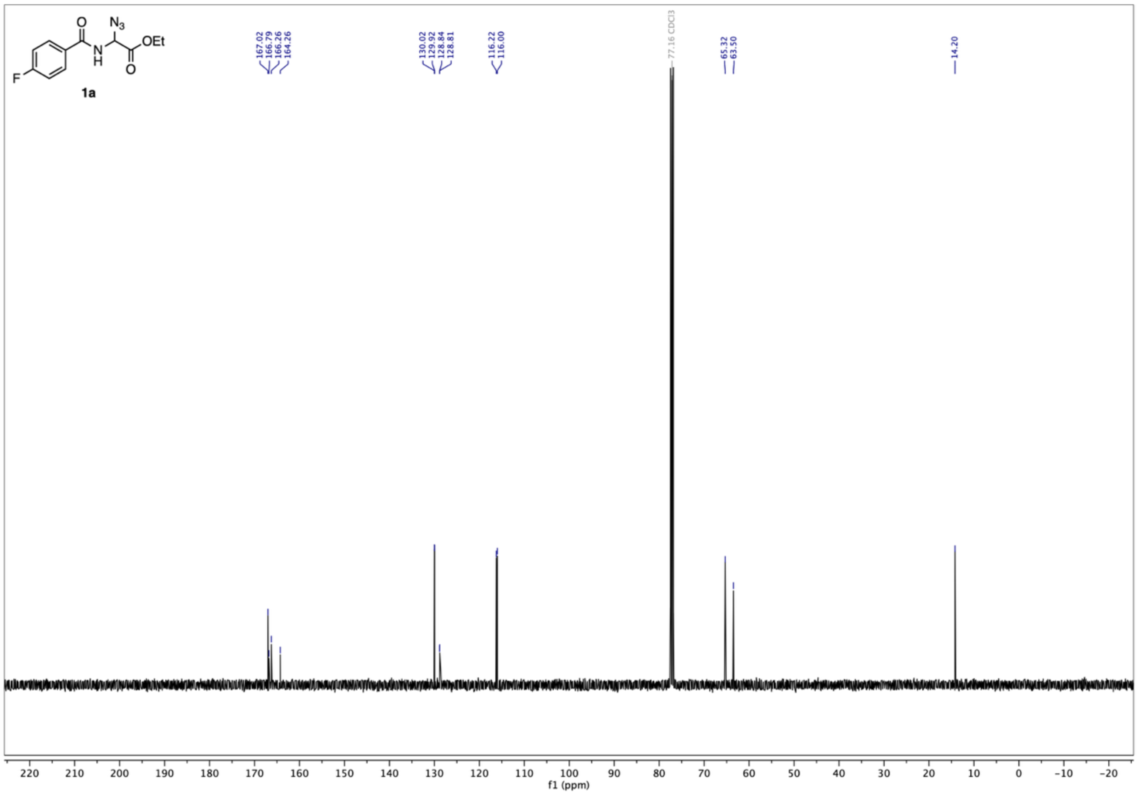

^{19}F NMR (376 MHz, $CDCl_3$) of ethyl 2-azido-2-(4-fluorobenzamido)acetate (**1a**)

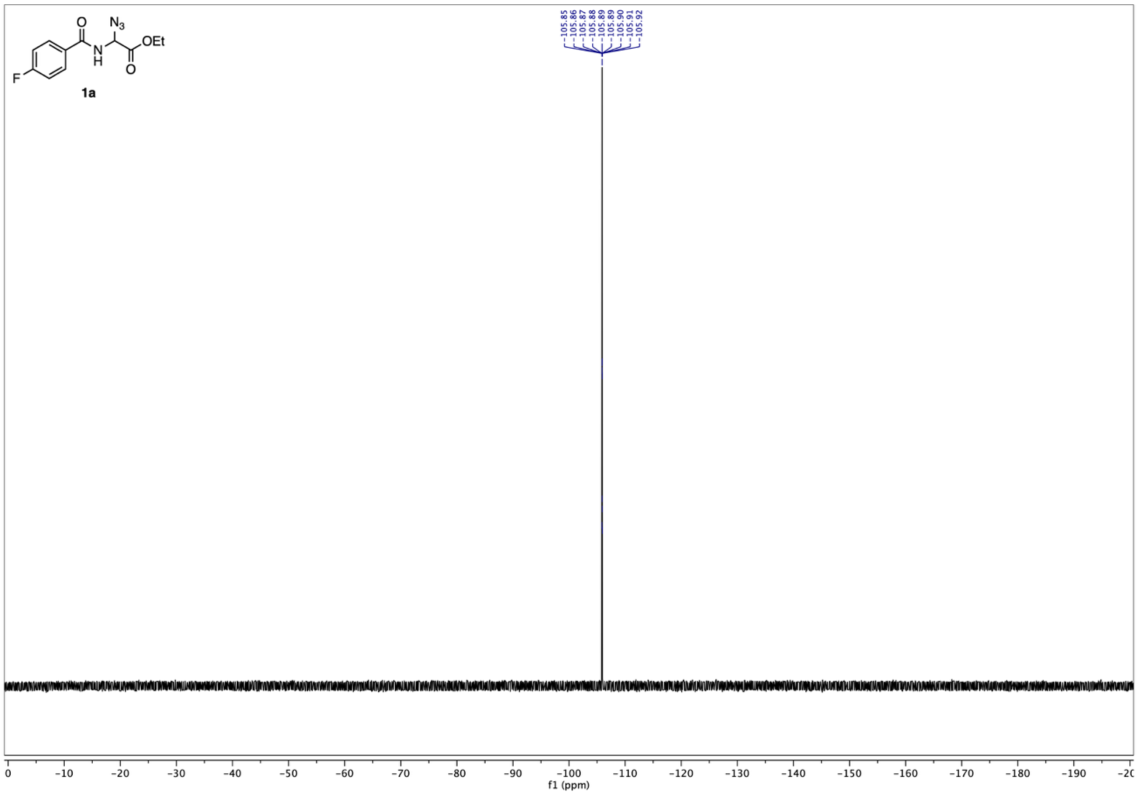

^{1}H NMR (400 MHz, DMSO-d_6) of ethyl 2-benzamido-2-hydroxyacetate (**S1b**)

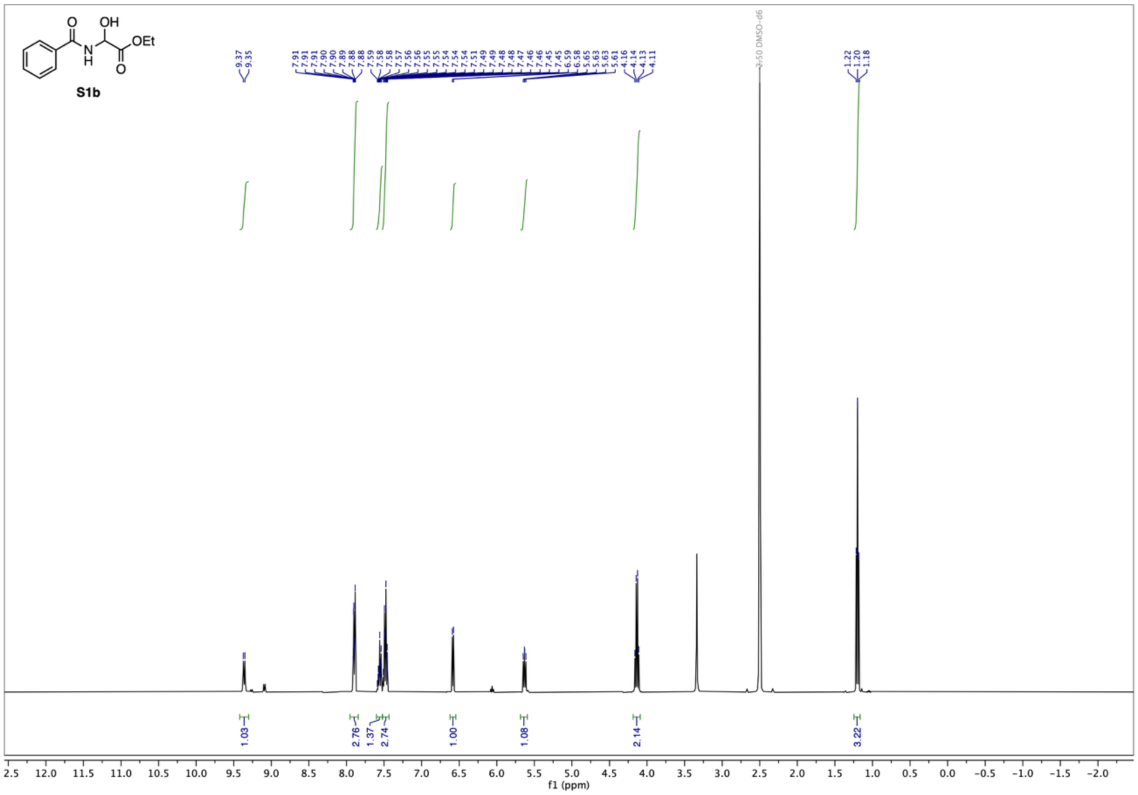

^{13}C{^{1}H} NMR (101 MHz, DMSO-d_6) of ethyl 2-benzamido-2-hydroxyacetate (**S1b**)

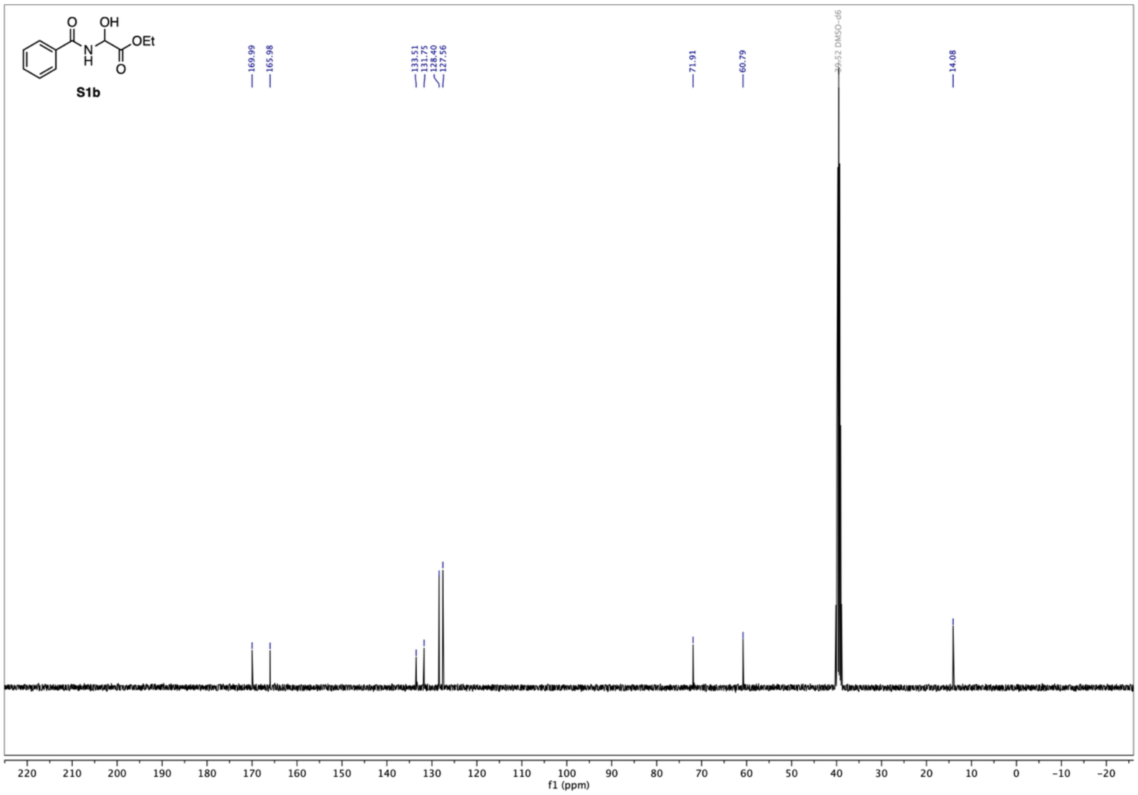

^{1}H NMR (400 MHz, DMSO-d_6) of ethyl 2-(4-(*tert*-butyl)benzamido)-2-hydroxyacetate (**S1d**)

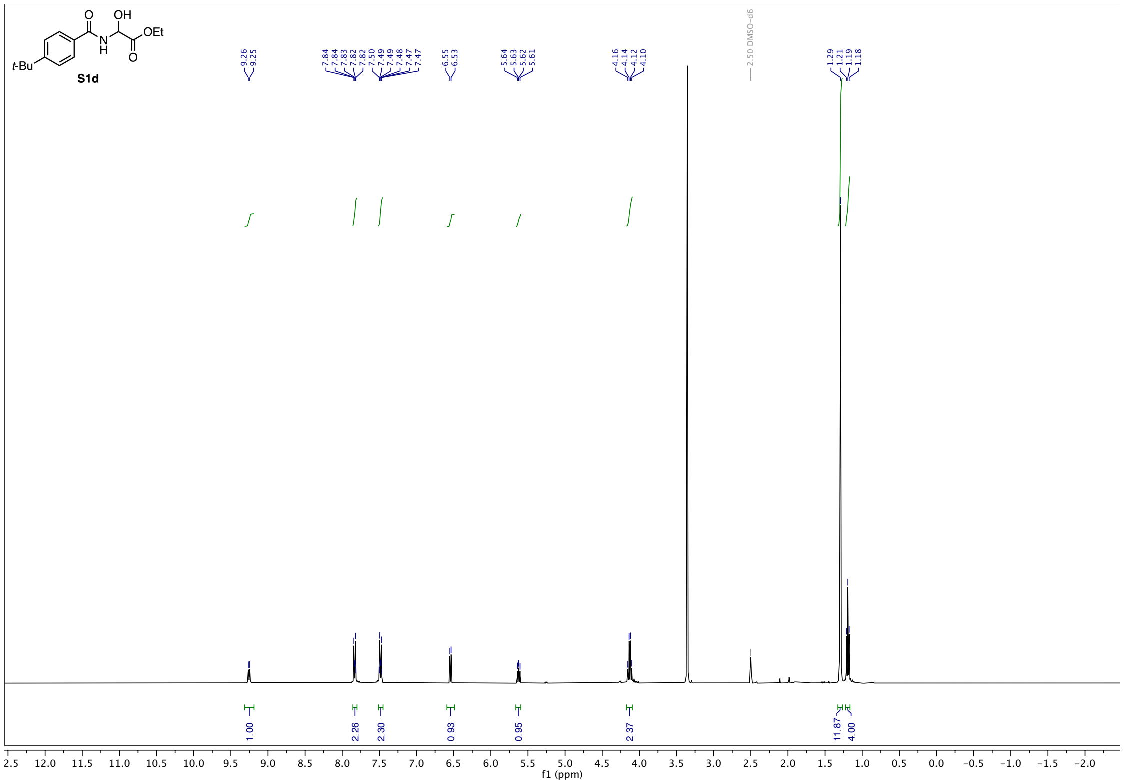

^{13}C{^{1}H} NMR (101 MHz, DMSO-d_6) of ethyl 2-(4-(*tert*-butyl)benzamido)-2-hydroxyacetate (**S1d**)

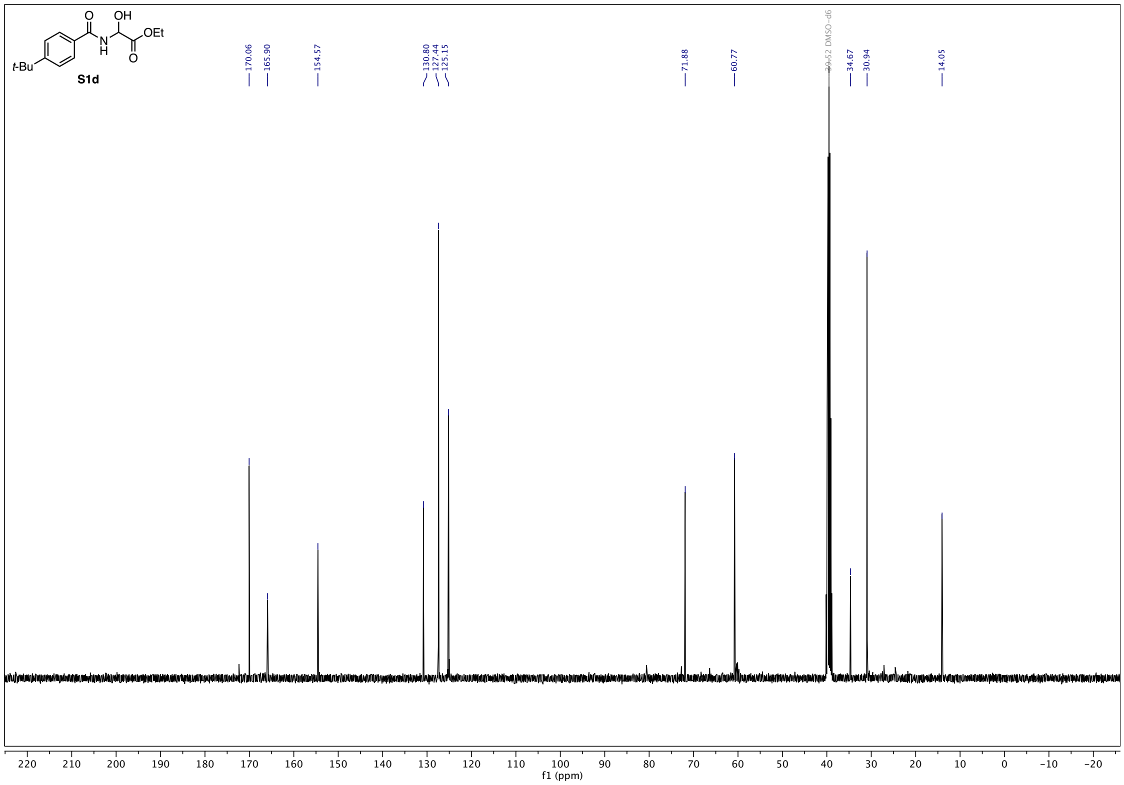

^1H NMR (400 MHz, DMSO-d_6) of ethyl 2-hydroxy-2-(4-methoxybenzamido)acetate (**S1e**)

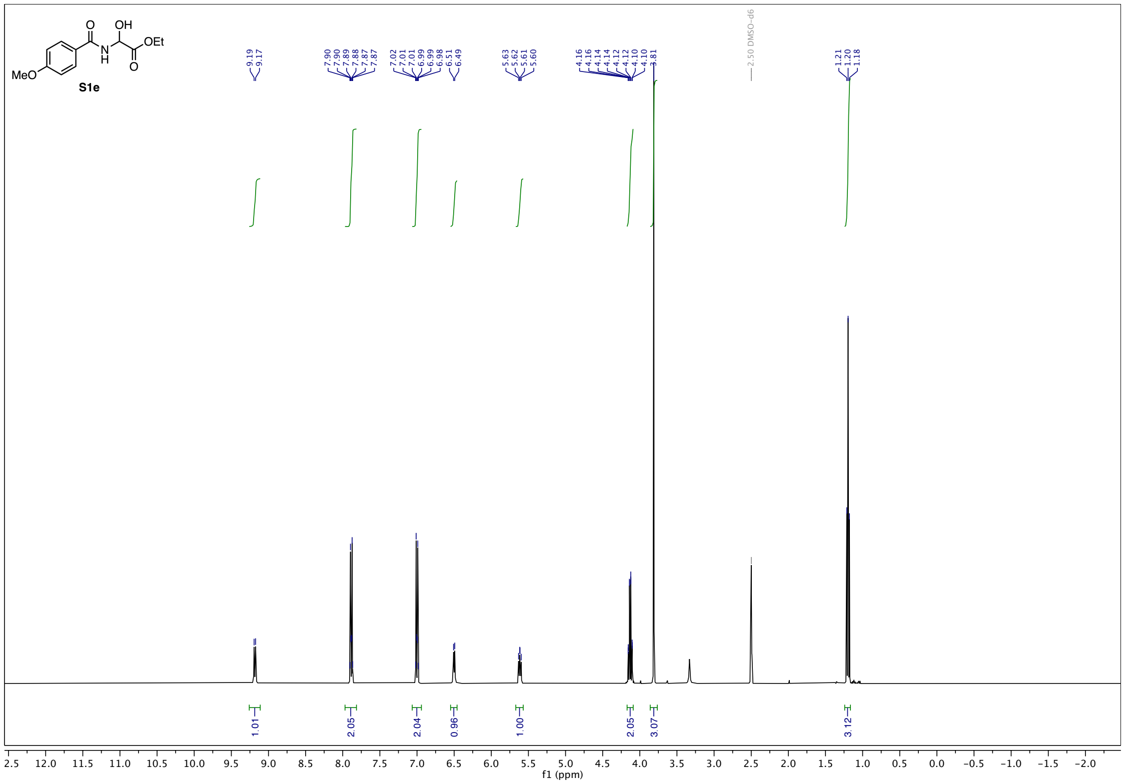

$^{13}C\{^1H\}$ NMR (101 MHz, DMSO-d_6) of ethyl 2-hydroxy-2-(4-methoxybenzamido)acetate (**S1e**)

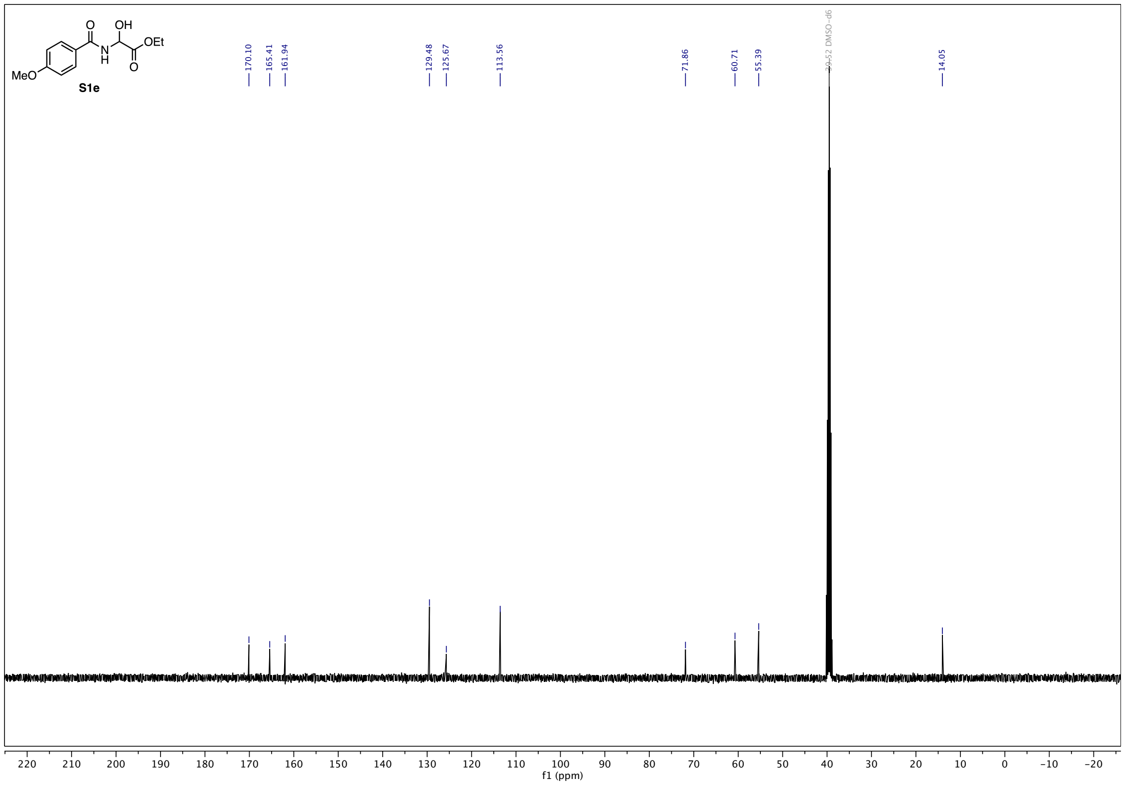

^{1}H NMR (400 MHz, DMSO-d_6) of ethyl 2-(4-bromobenzamido)-2-hydroxyacetate (**S1f**)

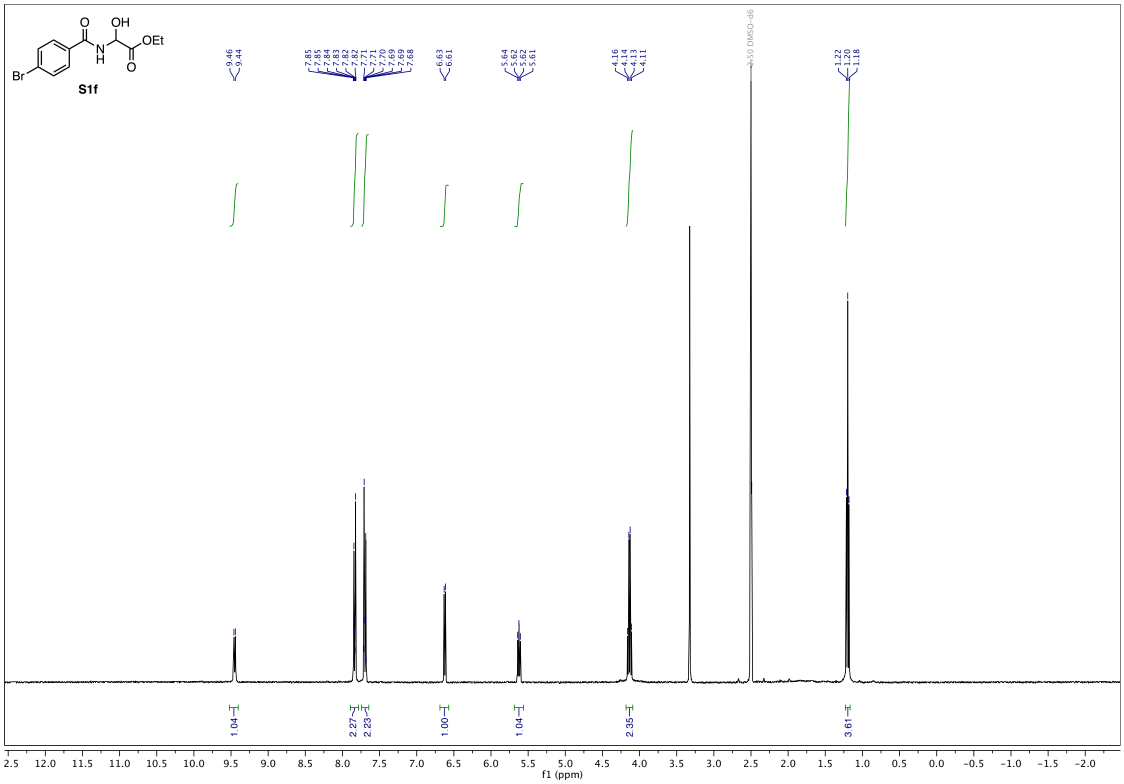

$^{13}C\{^{1}H\}$ NMR (101 MHz, DMSO-d_6) of ethyl 2-(4-bromobenzamido)-2-hydroxyacetate (**S1f**)

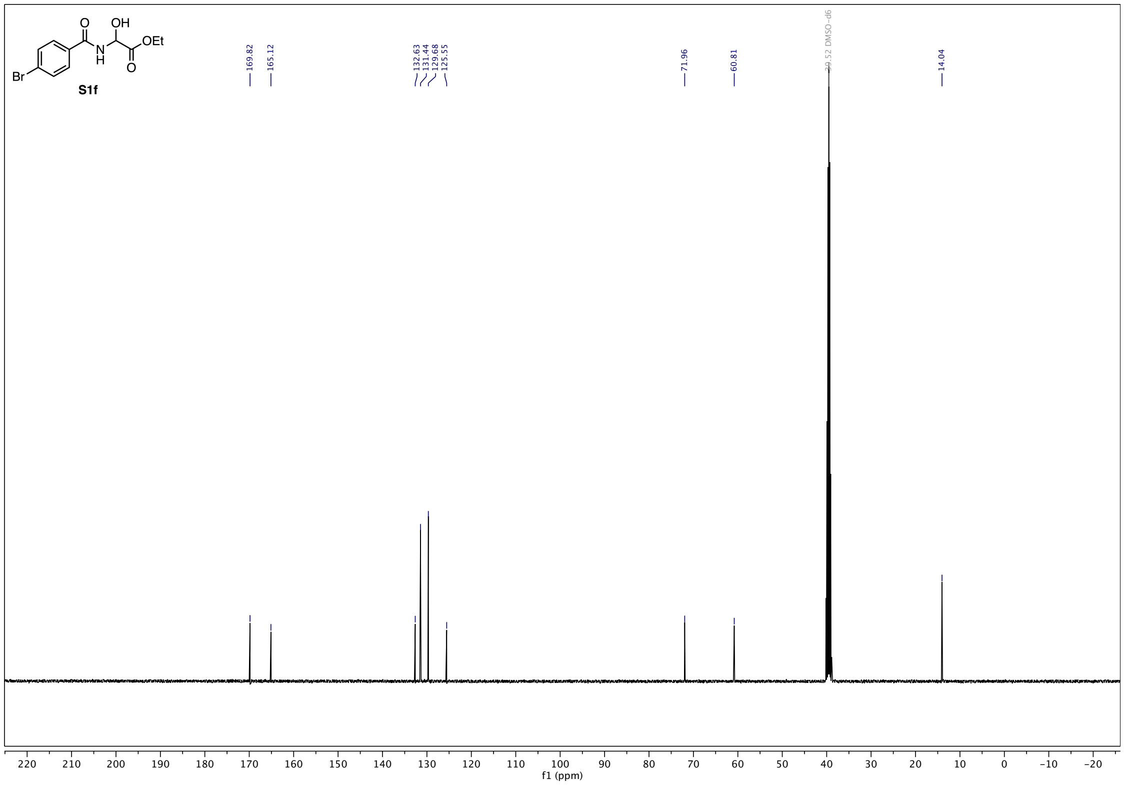

^{1}H NMR (400 MHz, DMSO-d_6) of ethyl 2-hydroxy-2-(4-(trifluoromethyl)benzamido)acetate (**S1g**)

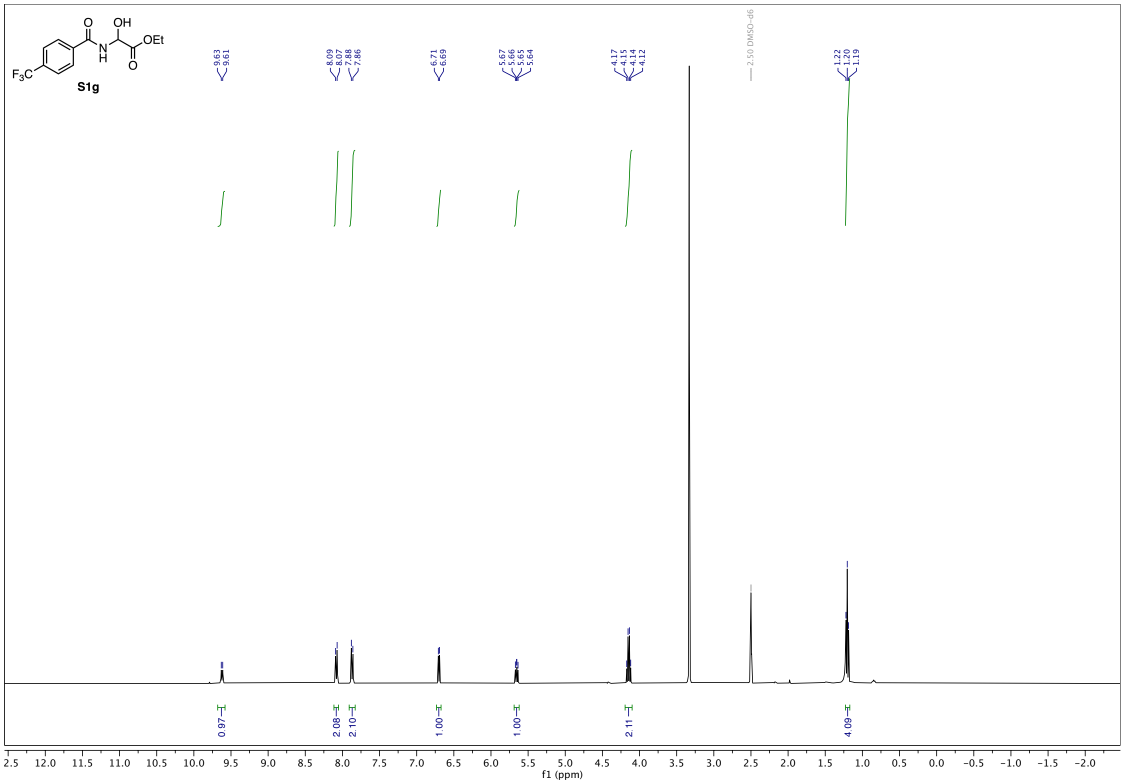

^{13}C{^{1}H} NMR (101 MHz, DMSO-d_6) of ethyl 2-hydroxy-2-(4-(trifluoromethyl)benzamido)acetate (**S1g**)

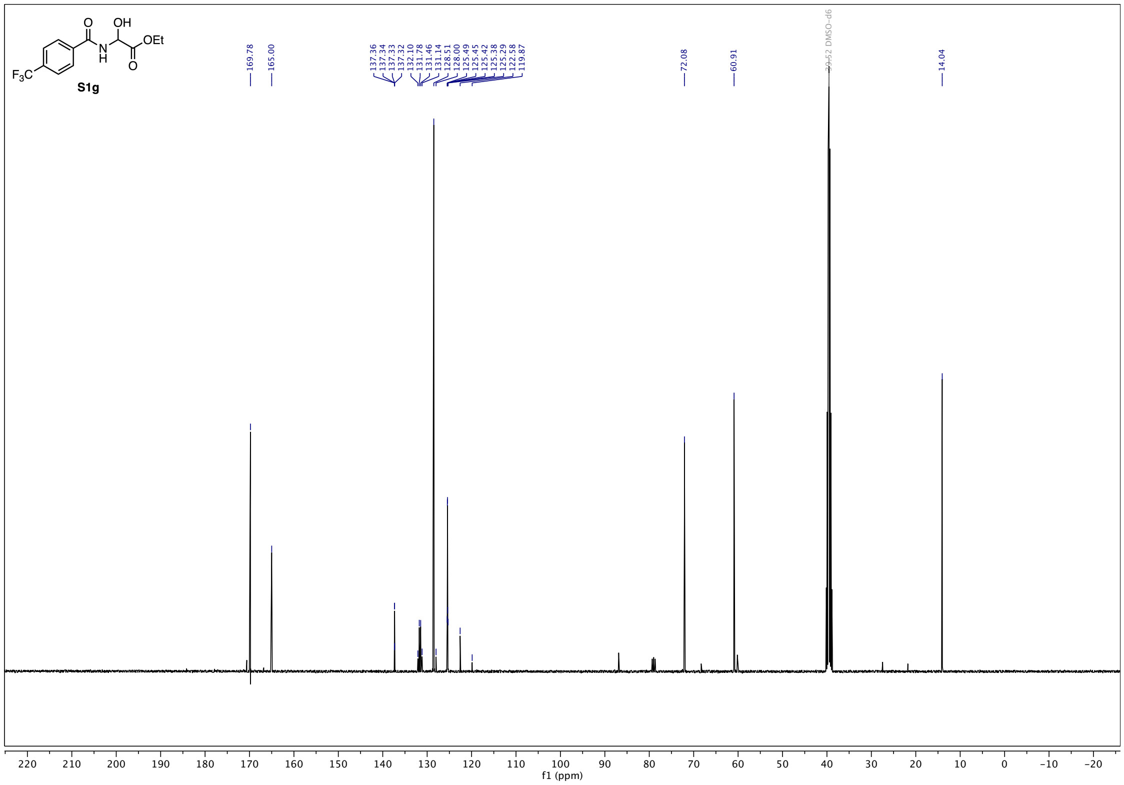

^{19}F NMR (376 MHz, DMSO-d_6) of ethyl 2-hydroxy-2-(4-(trifluoromethyl)benzamido)acetate (**S1g**)

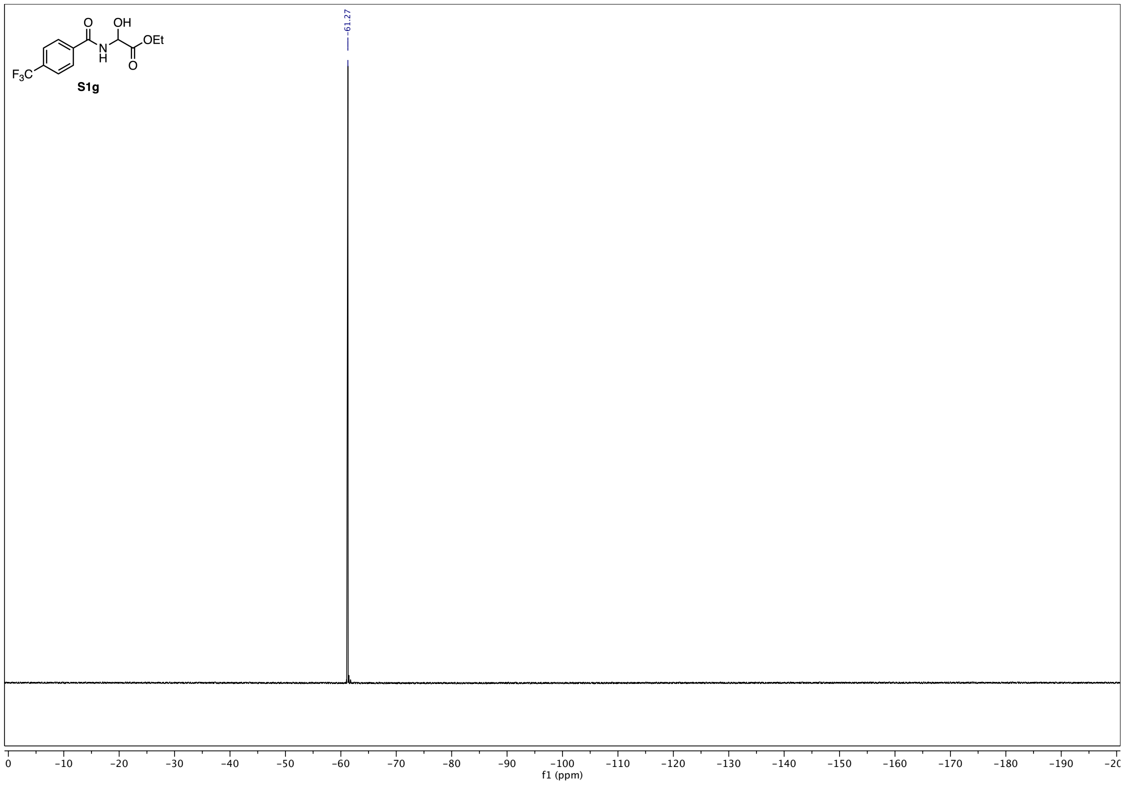

^1H NMR (400 MHz, $CDCl_3$) of ethyl 2-azido-2-(4-(trifluoromethyl)benzamido)acetate (**1g**)

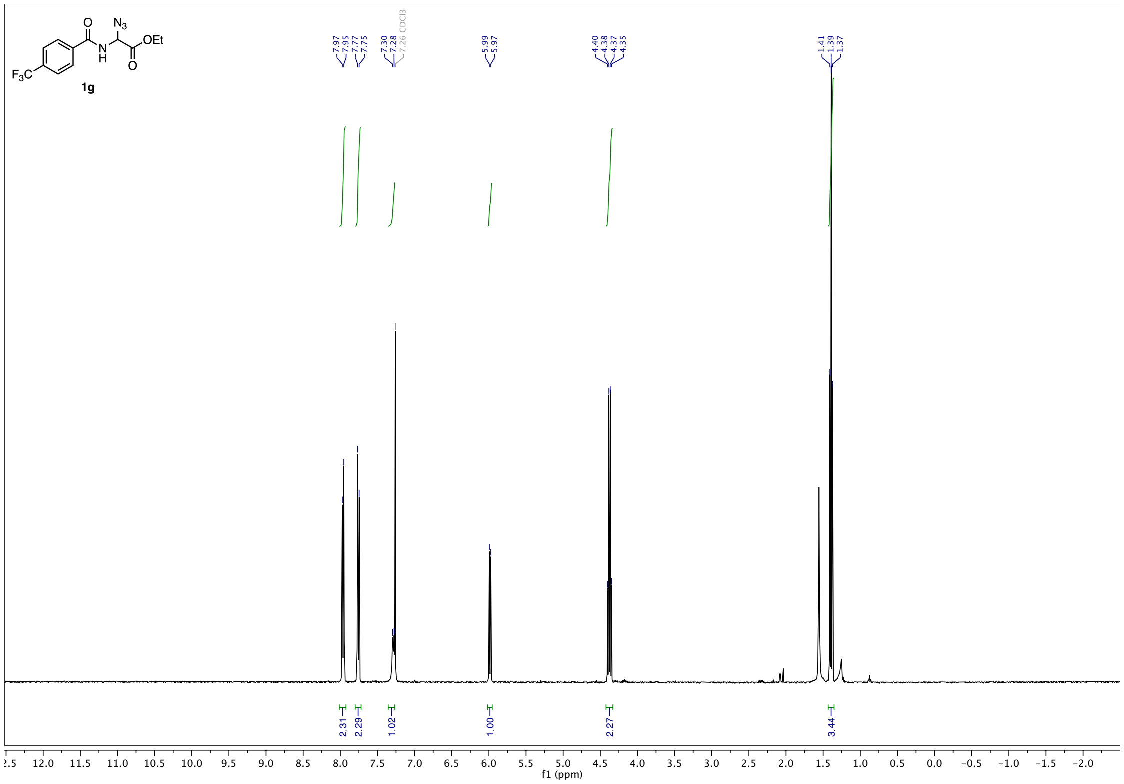

$^{13}C\{^1H\}$ NMR (101 MHz, $CDCl_3$) of ethyl 2-azido-2-(4-(trifluoromethyl)benzamido)acetate (**1g**)

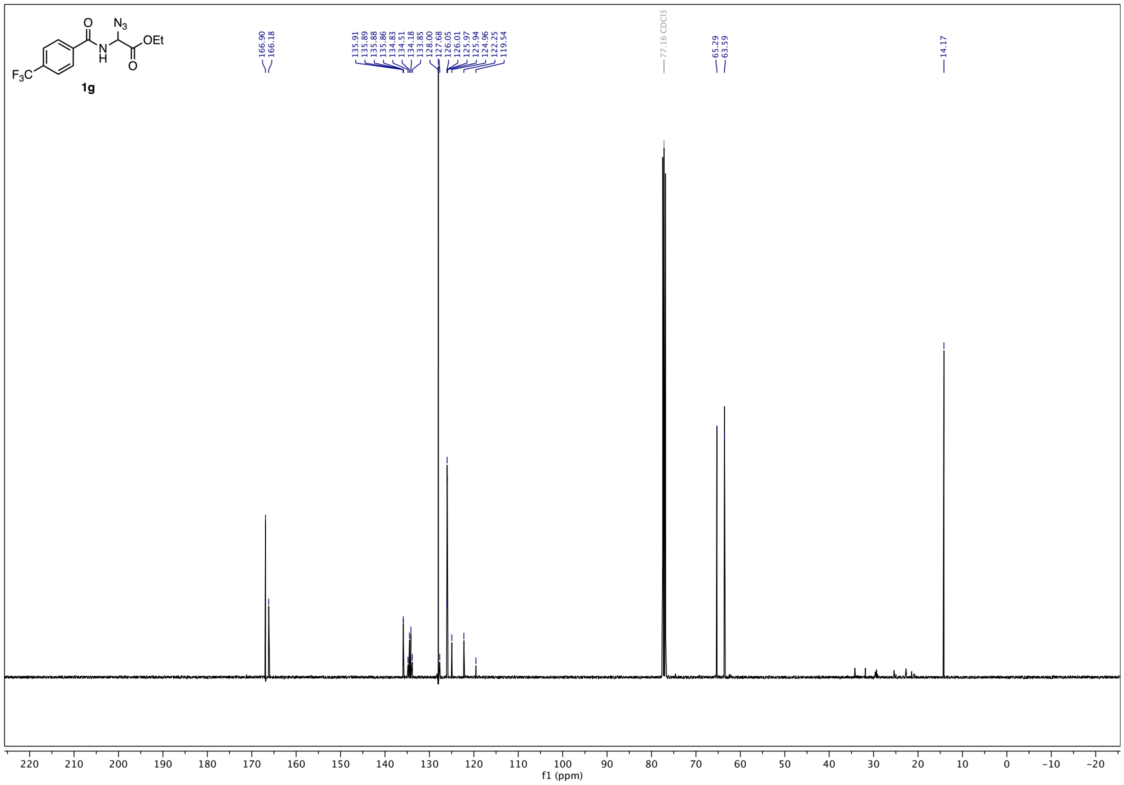

^{19}F NMR (376 MHz, $CDCl_3$) of ethyl 2 ethyl 2-azido-2-(4-(trifluoromethyl)benzamido)acetate (**1g**)

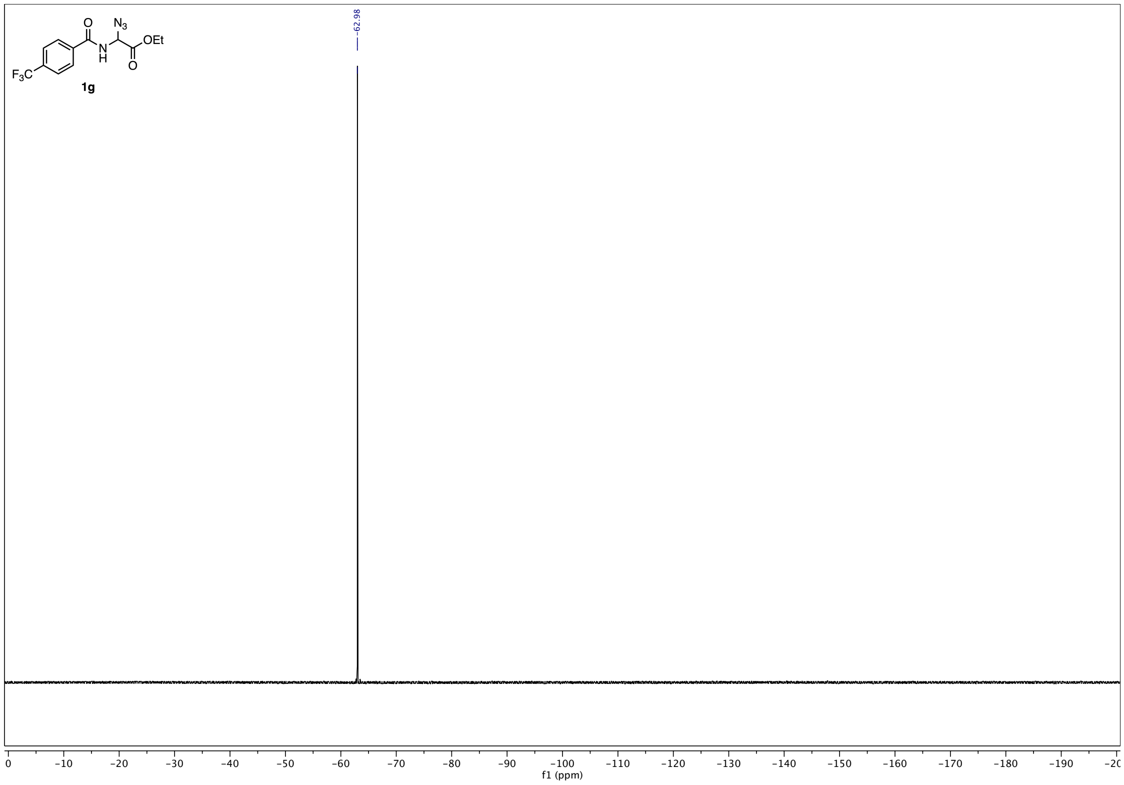

^{1}H NMR (400 MHz, DMSO-d_6) of ethyl 2-(4-cyanobenzamido)-2-hydroxyacetate (**S1h**)

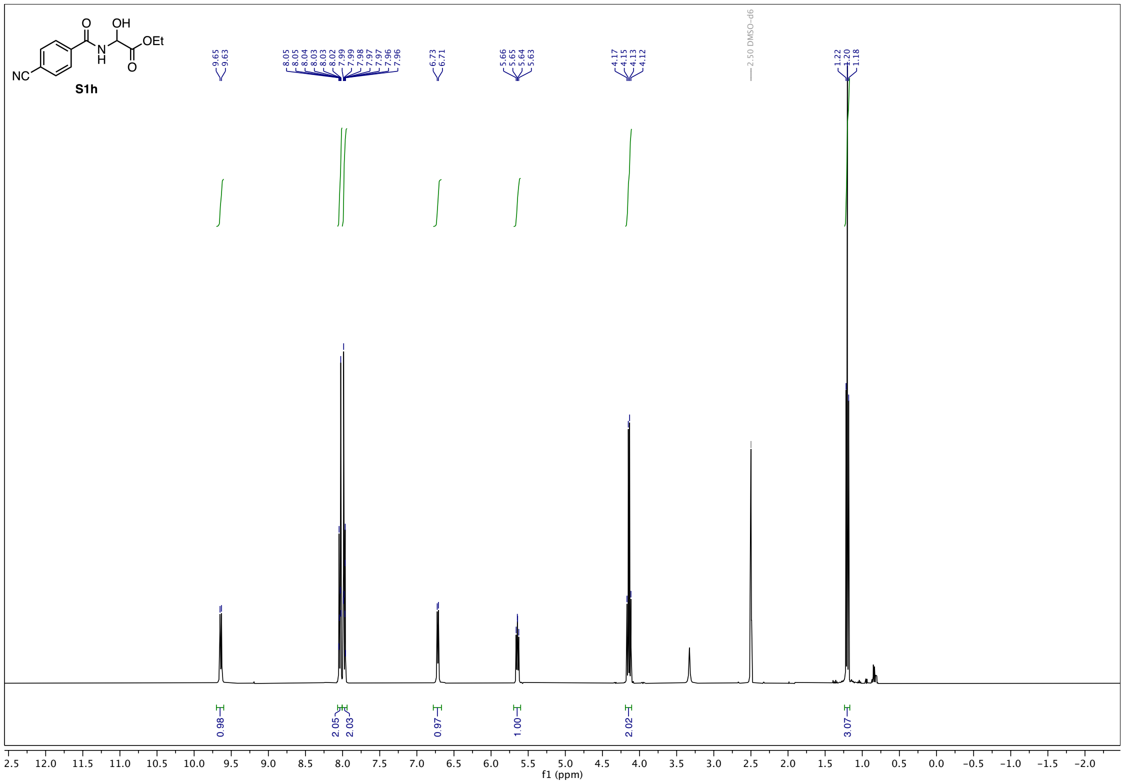

$^{13}C\{^{1}H\}$ NMR (101 MHz, DMSO-d_6) of ethyl 2-(4-cyanobenzamido)-2-hydroxyacetate (**S1h**)

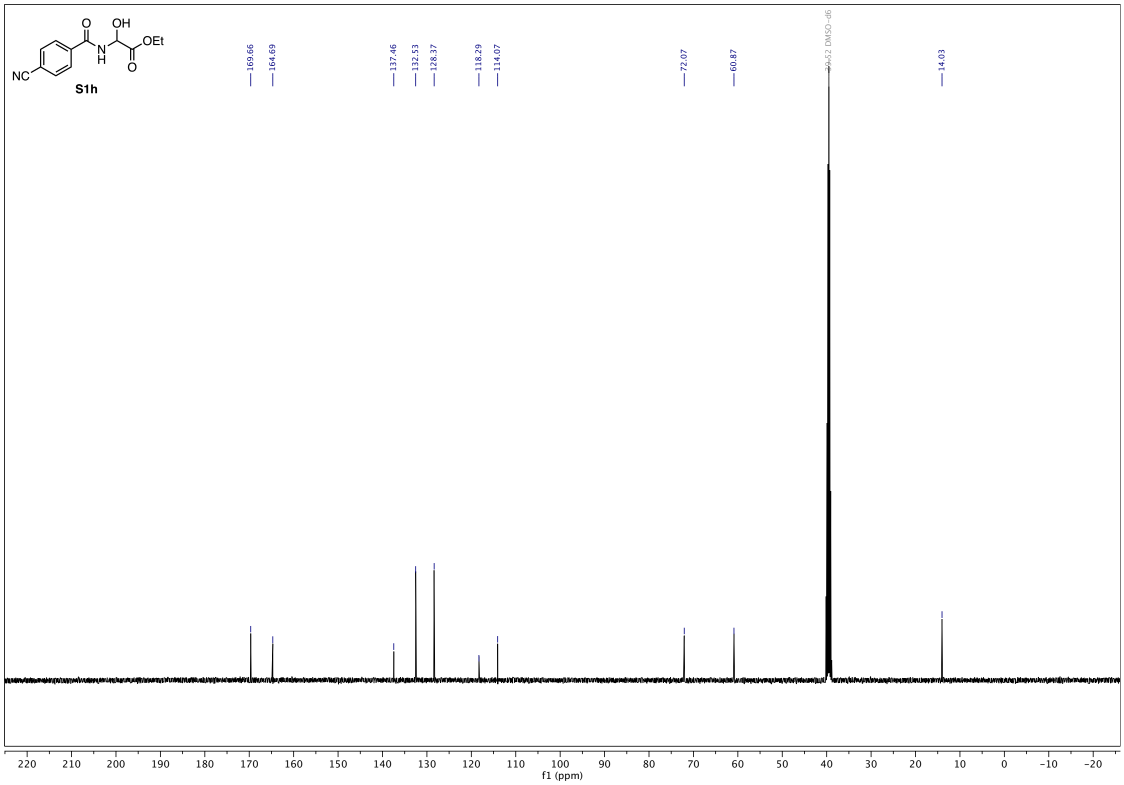

^{1}H NMR (400 MHz, DMSO-d_6) of ethyl 2-(3-fluorobenzamido)-2-hydroxyacetate (**S1i**)

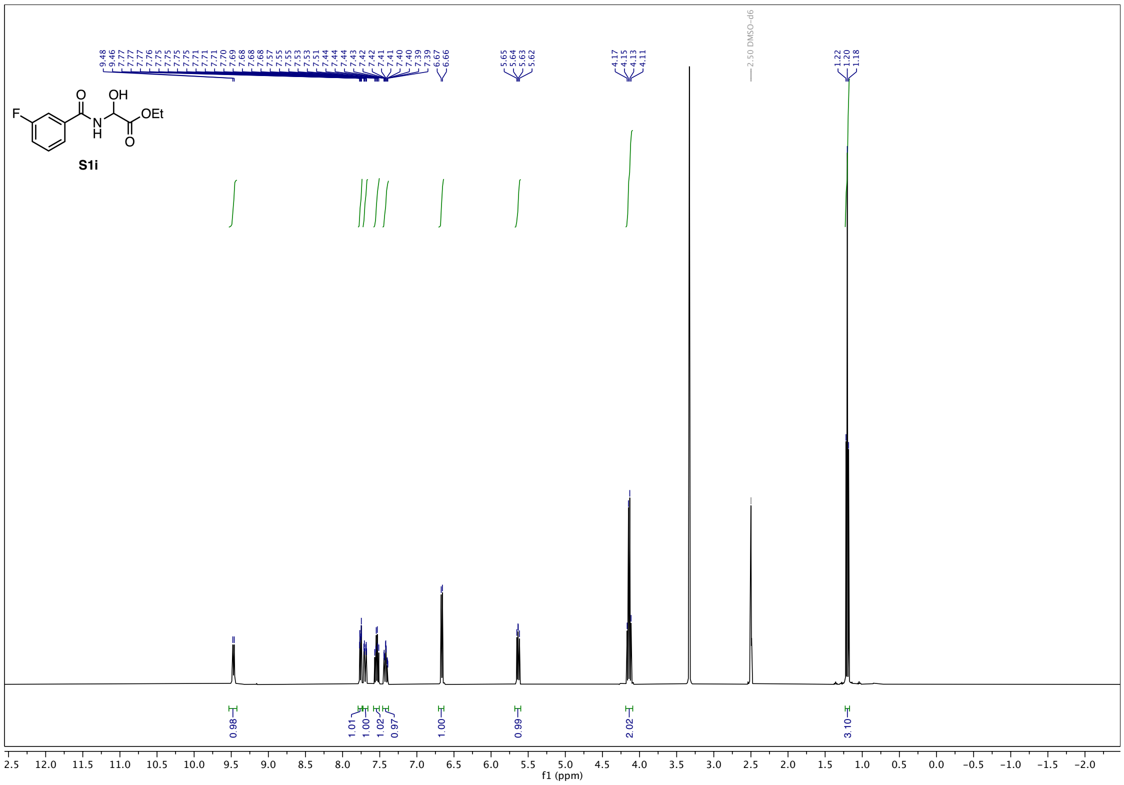

$^{13}C\{^{1}H\}$ NMR (101 MHz, DMSO-d_6) of ethyl 2-(3-fluorobenzamido)-2-hydroxyacetate (**S1i**)

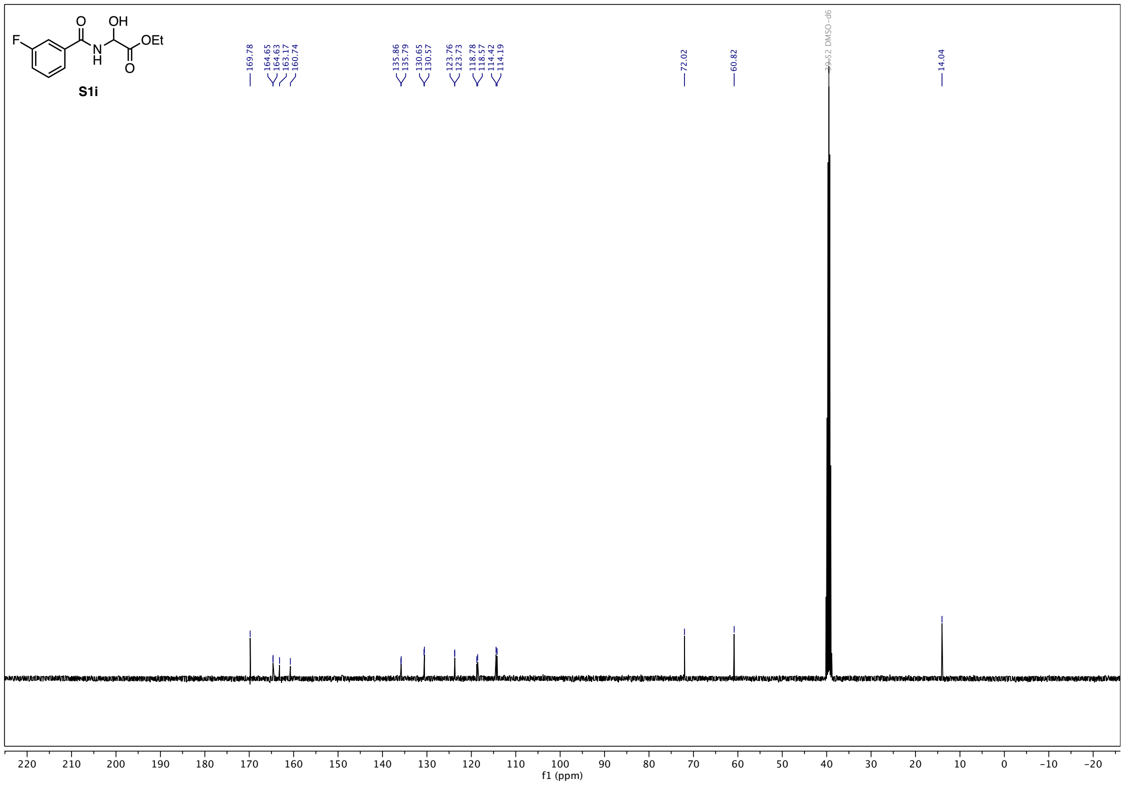

^{19}F NMR (376 MHz, DMSO-d_6) of ethyl 2-(3-fluorobenzamido)-2-hydroxyacetate (**S1i**)

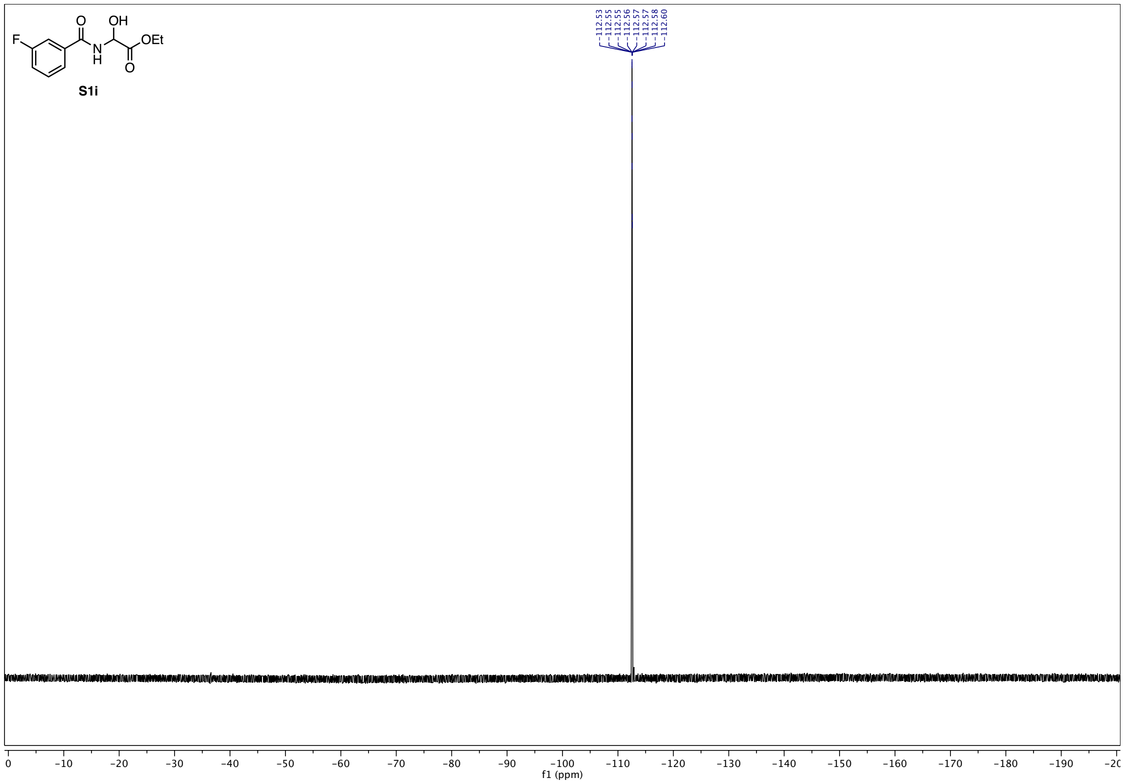

^{1}H NMR (400 MHz, $CDCl_3$) of ethyl 2-azido-2-(3-fluorobenzamido)acetate (**1i**)

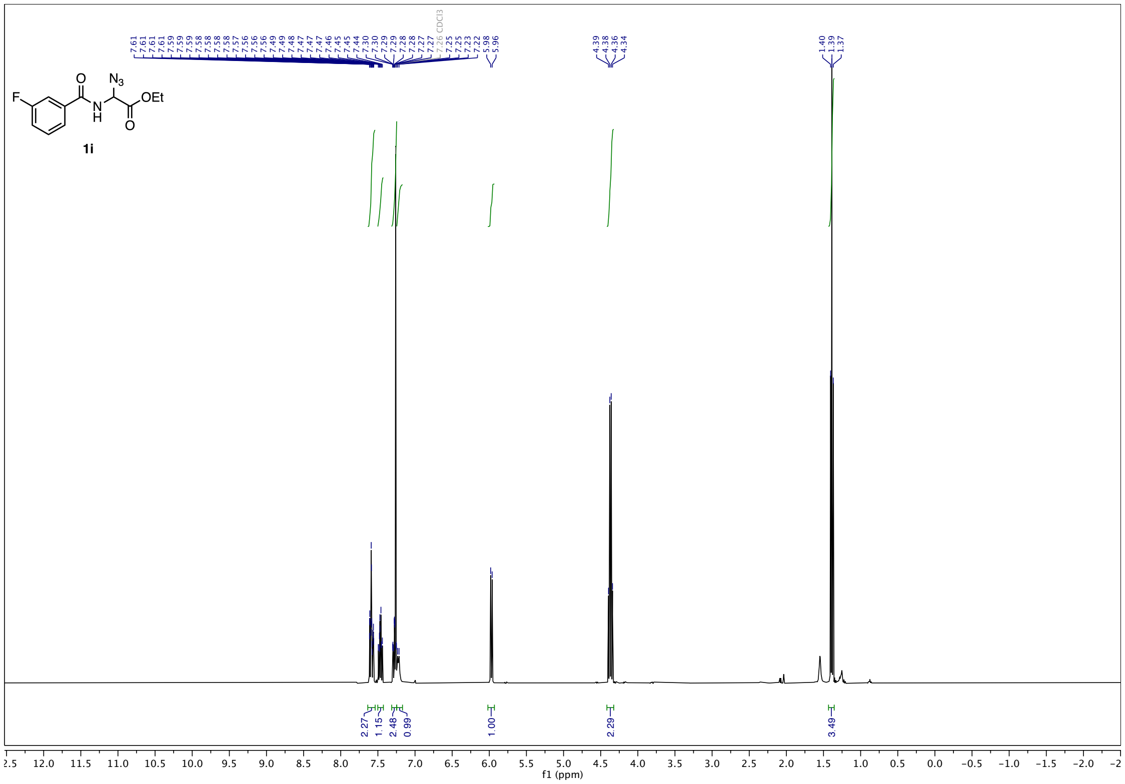

$^{13}C\{^{1}H\}$ NMR (101 MHz, $CDCl_3$) of ethyl 2-azido-2-(3-fluorobenzamido)acetate (**1i**)

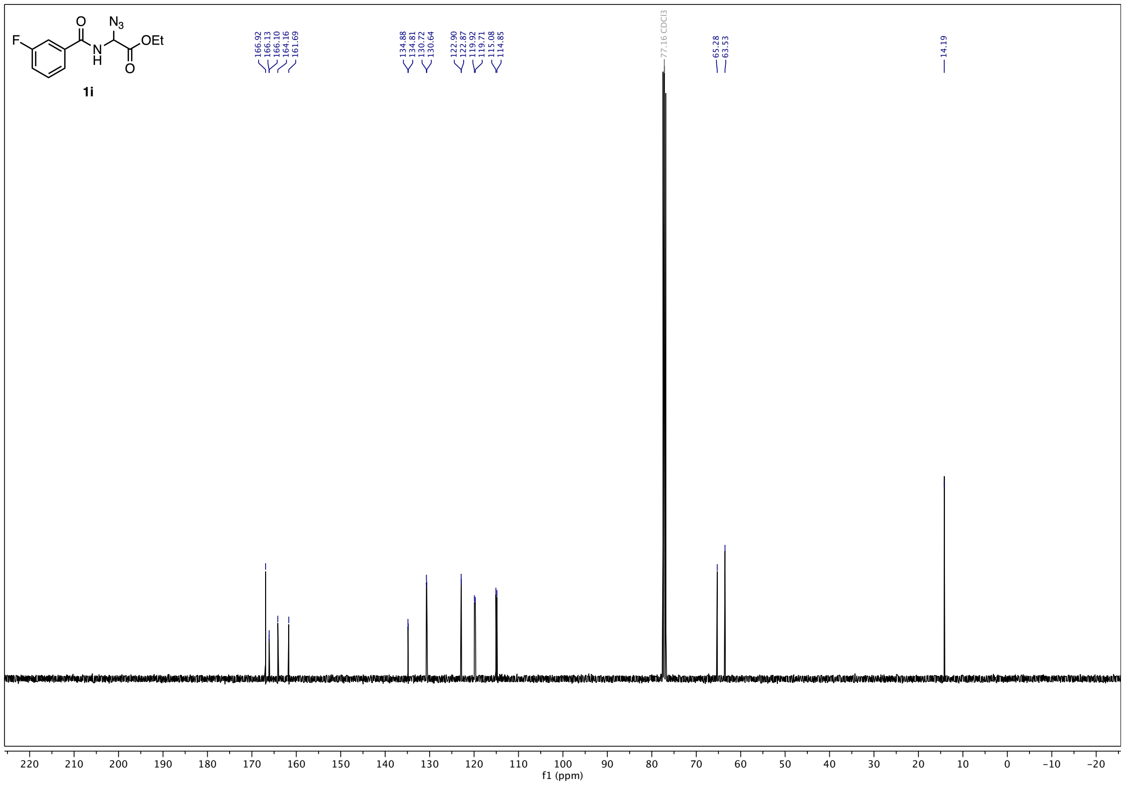

^{19}F NMR (376 MHz, $CDCl_3$) of ethyl 2-azido-2-(3-fluorobenzamido)acetate (**1i**)

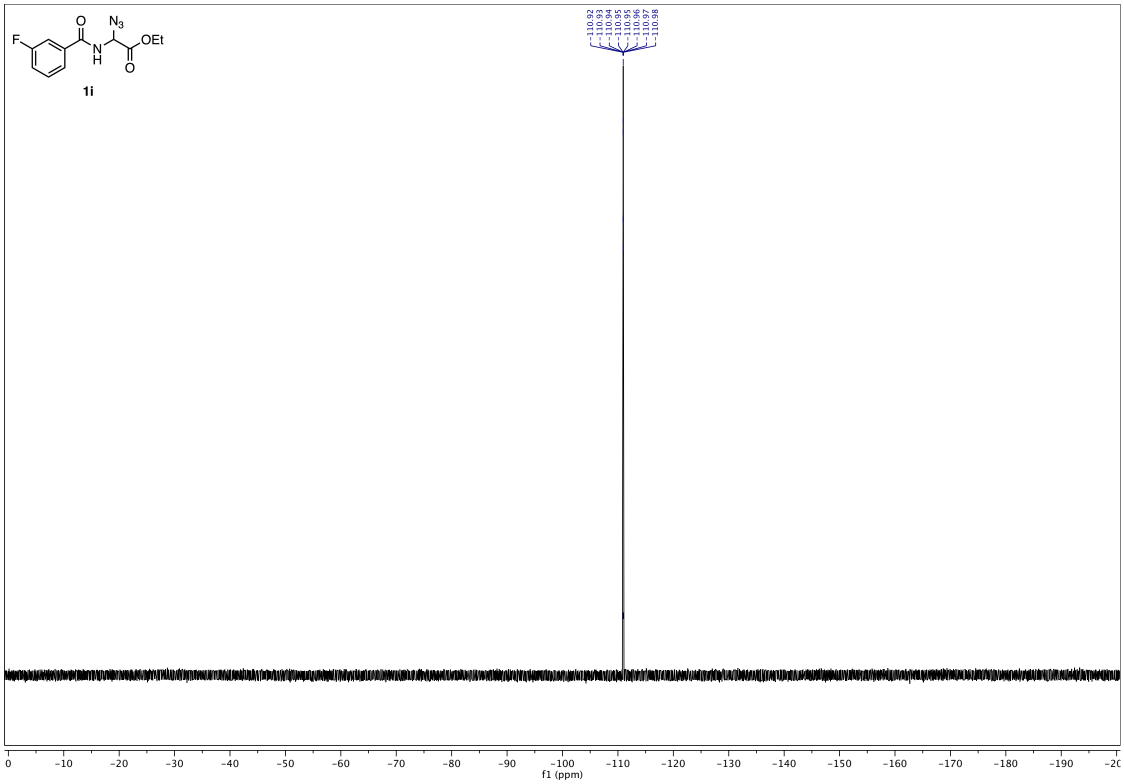

^{1}H NMR (400 MHz, DMSO-d_6) of ethyl 2-(2-chlorobenzamido)-2-hydroxyacetate (**S1j**)

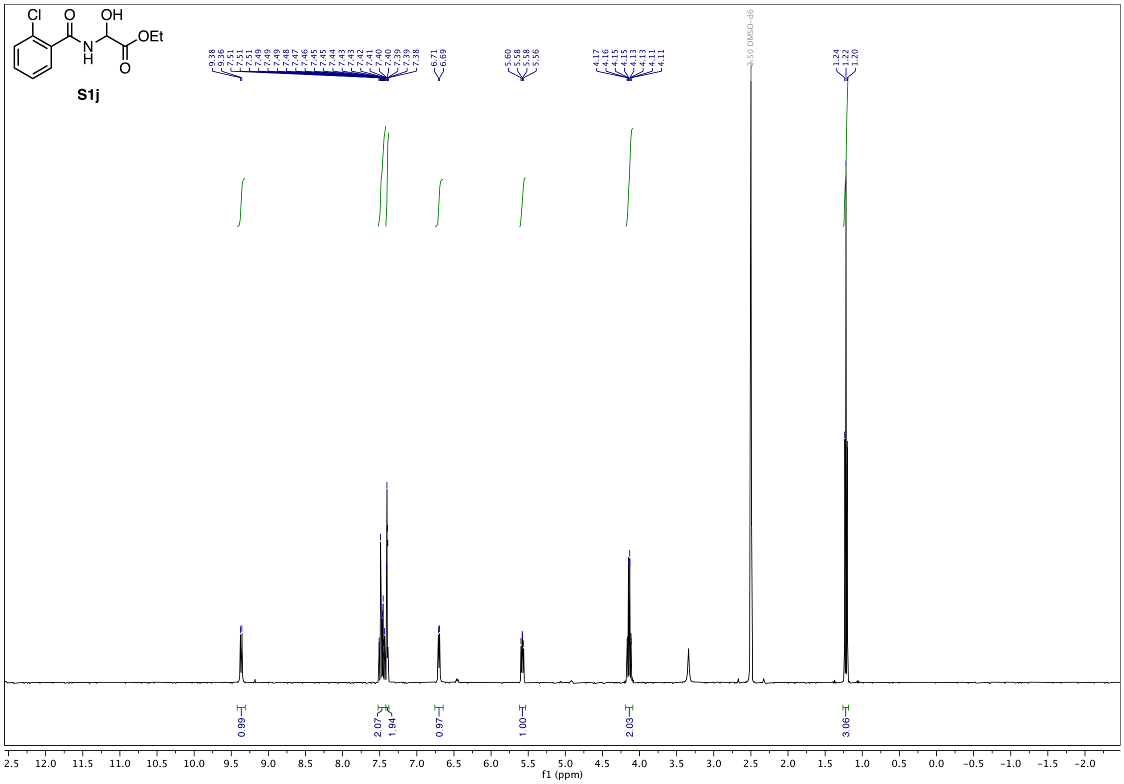

$^{13}C\{^{1}H\}$ NMR (101 MHz, DMSO-d_6) of ethyl 2-(2-chlorobenzamido)-2-hydroxyacetate (**S1j**)

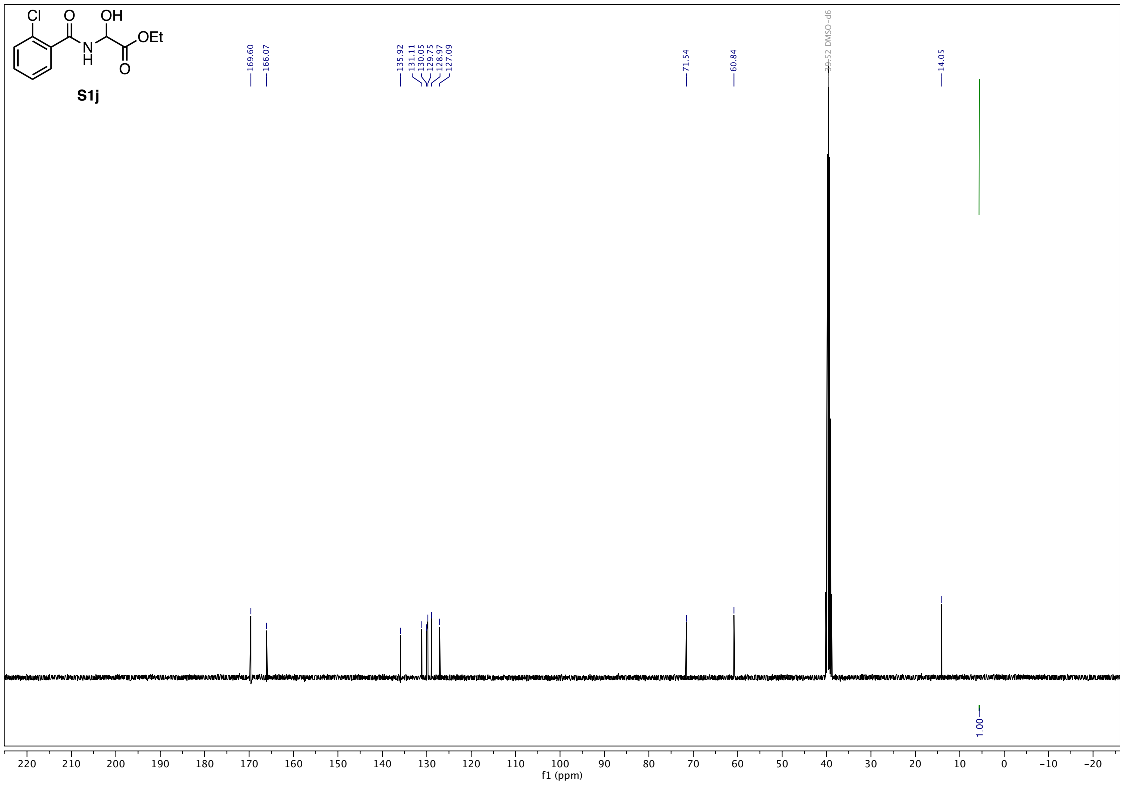

^{1}H NMR (400 MHz, $CDCl_3$) of ethyl 2-azido-2-(2-chlorobenzamido)acetate (**1j**)

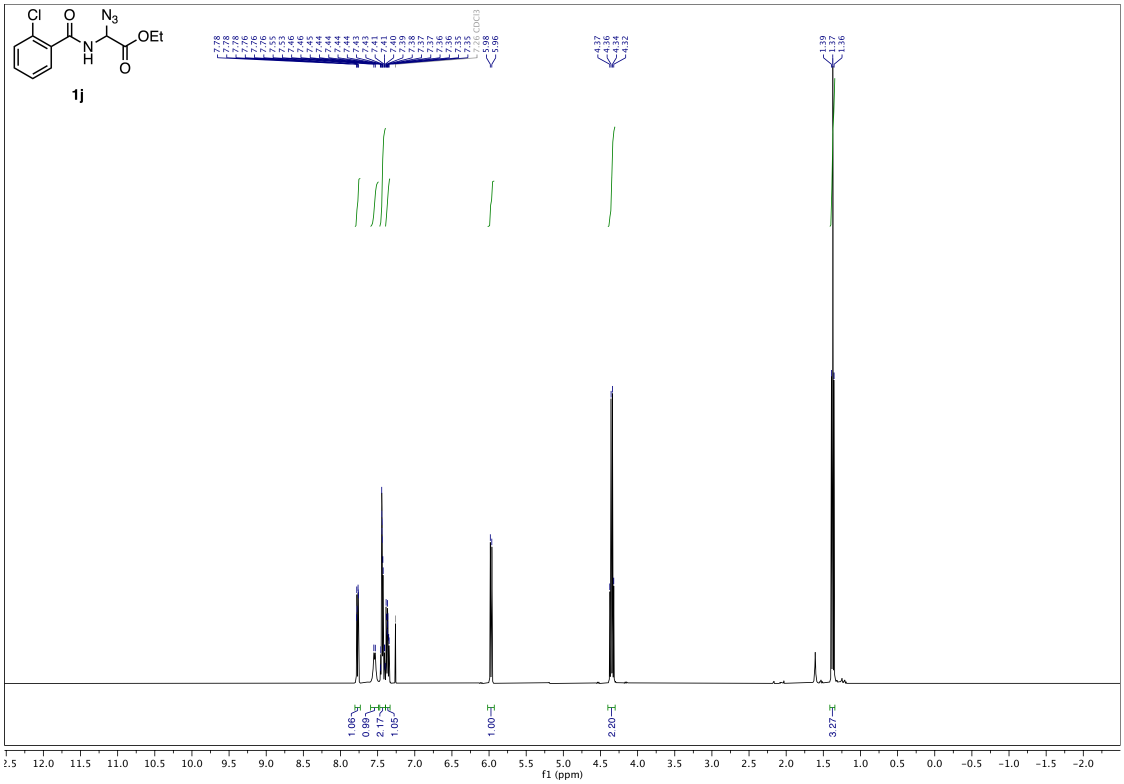

^{13}C{^{1}H} NMR (101 MHz, $CDCl_3$) of ethyl 2-azido-2-(2-chlorobenzamido)acetate (**1j**)

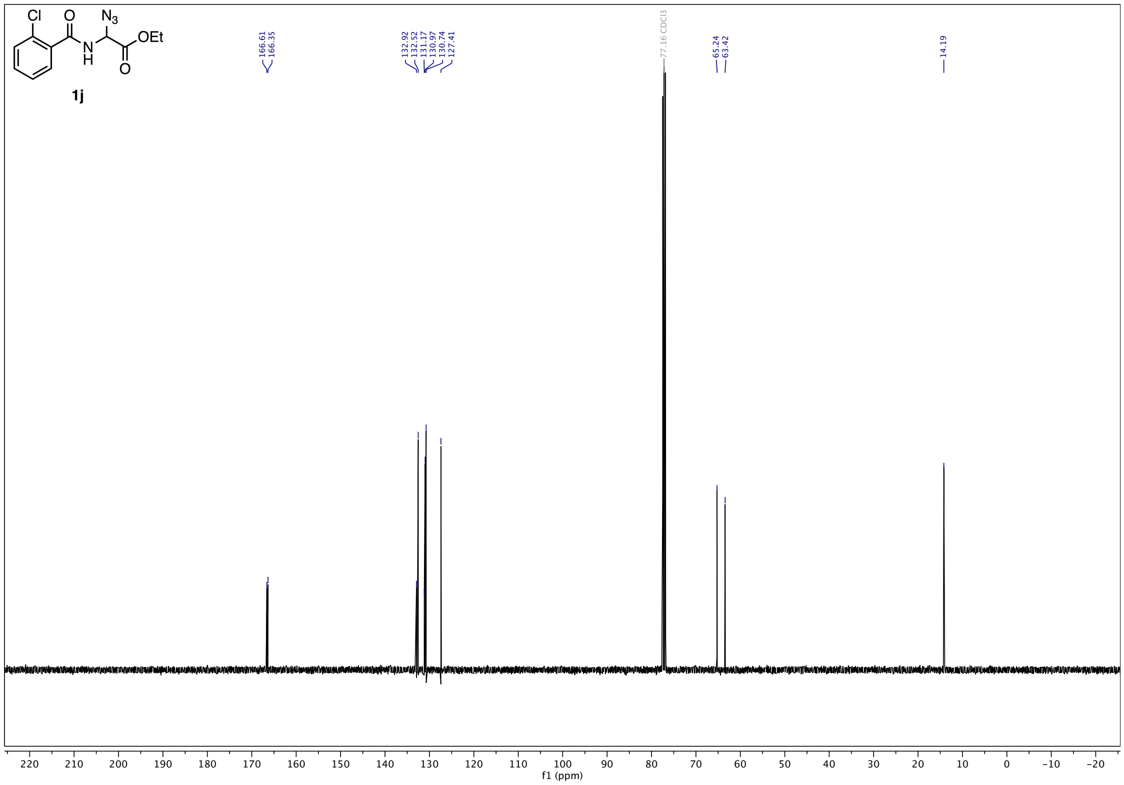

^{1}H NMR (400 MHz, $CDCl_3$) of *N*-(azido(pyridin-2-yl)methyl)benzamide (**1k**)

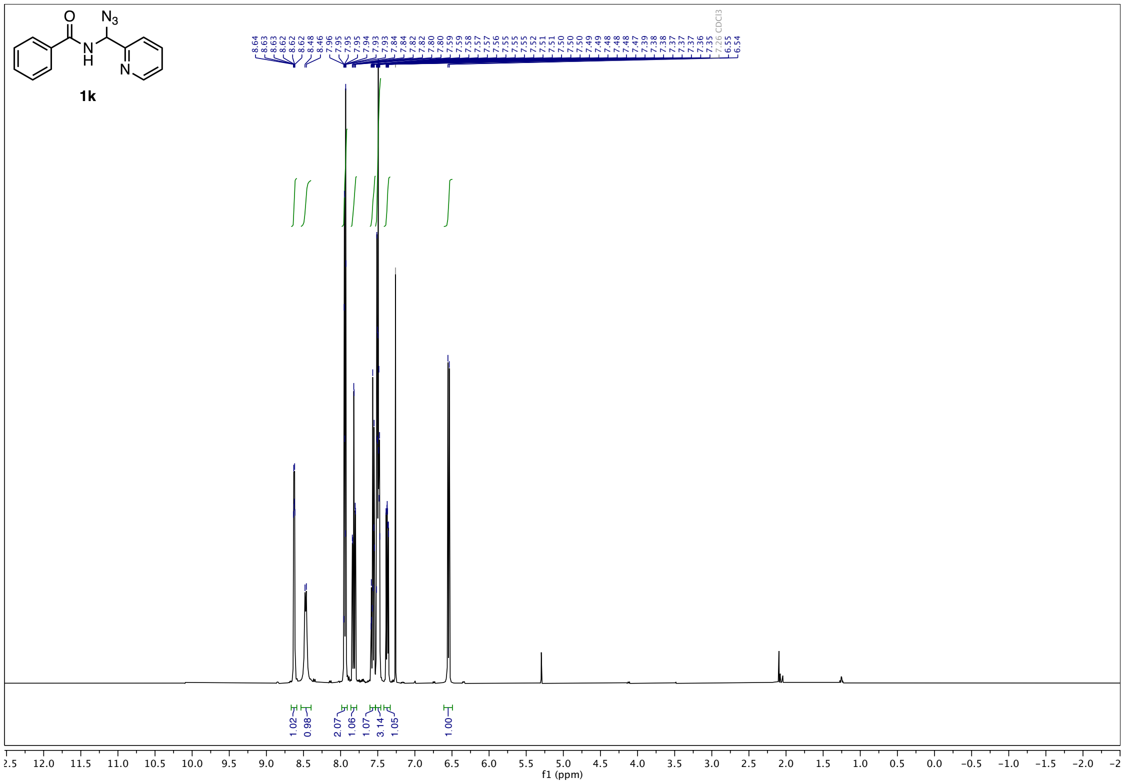

^{13}C{^{1}H} NMR (101 MHz, $CDCl_3$) of *N*-(azido(pyridin-2-yl)methyl)benzamide (**1k**)

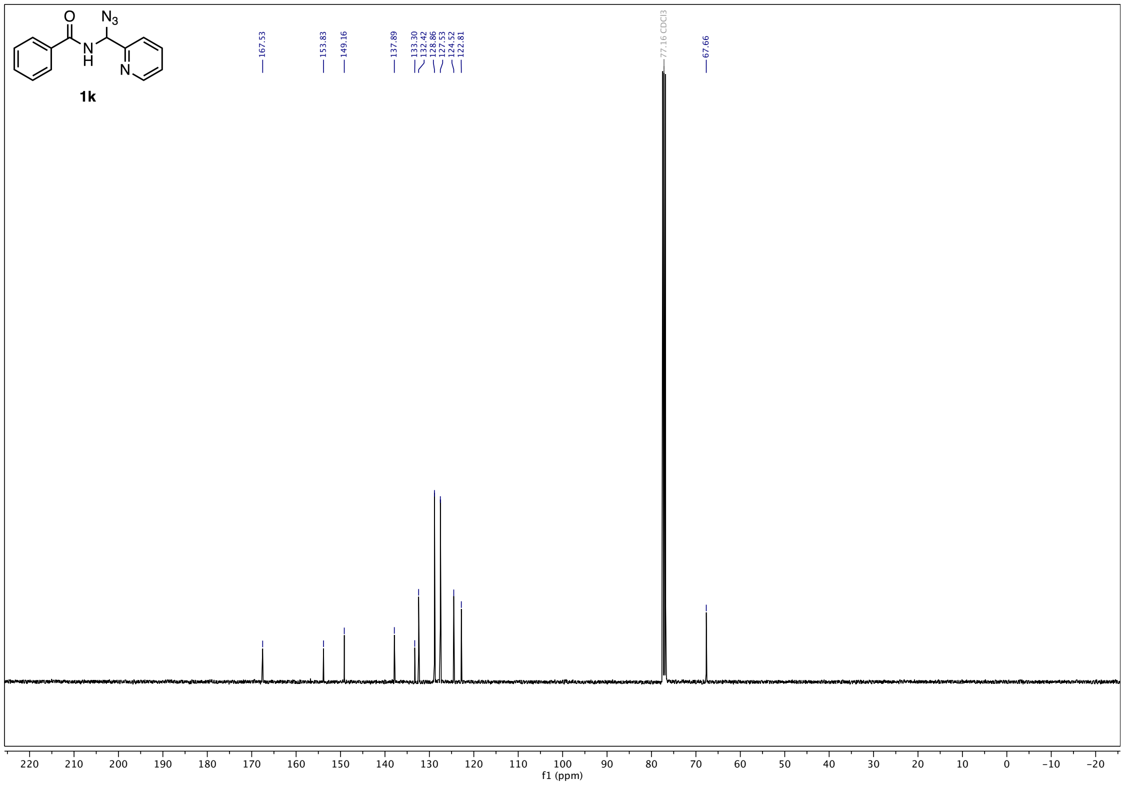

^{1}H NMR (400 MHz, $CDCl_3$) of ethyl 7-fluoro-4-oxo-3,4-dihydroquinazoline-2-carboxylate (**2a**)

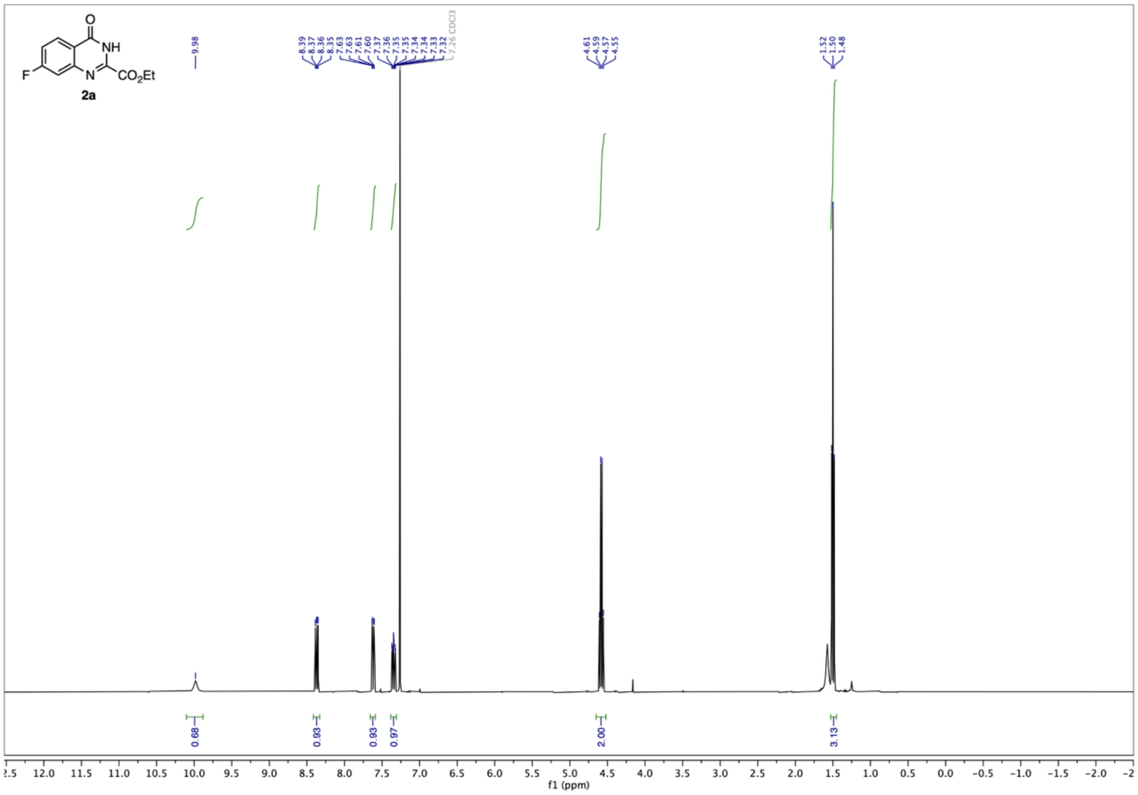

^{13}C{^{1}H} NMR (101 MHz, $CDCl_3$) of ethyl 7-fluoro-4-oxo-3,4-dihydroquinazoline-2-carboxylate (**2a**)

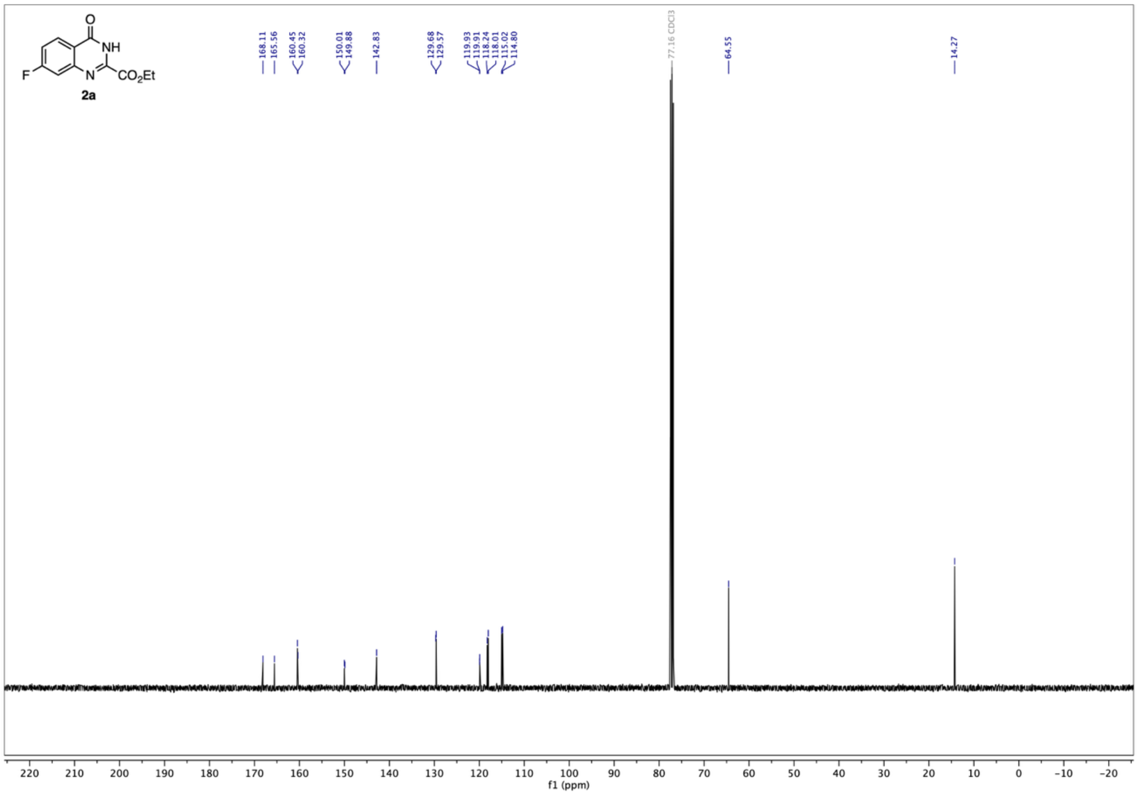

^{19}F NMR (376 MHz, $CDCl_3$) of ethyl 7-fluoro-4-oxo-3,4-dihydroquinazoline-2-carboxylate (**2a**)

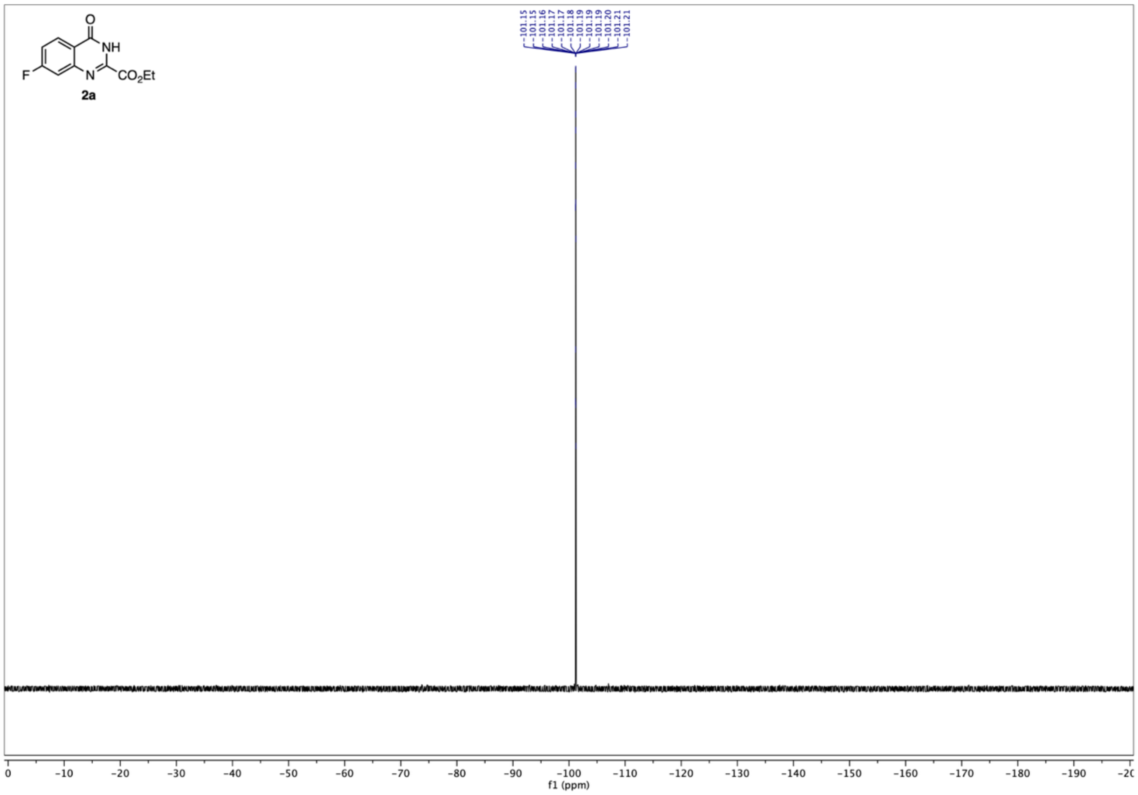

^{1}H NMR (400 MHz, $CDCl_3$) of ethyl 4-oxo-3,4-dihydroquinazoline-2-carboxylate (**2b**)

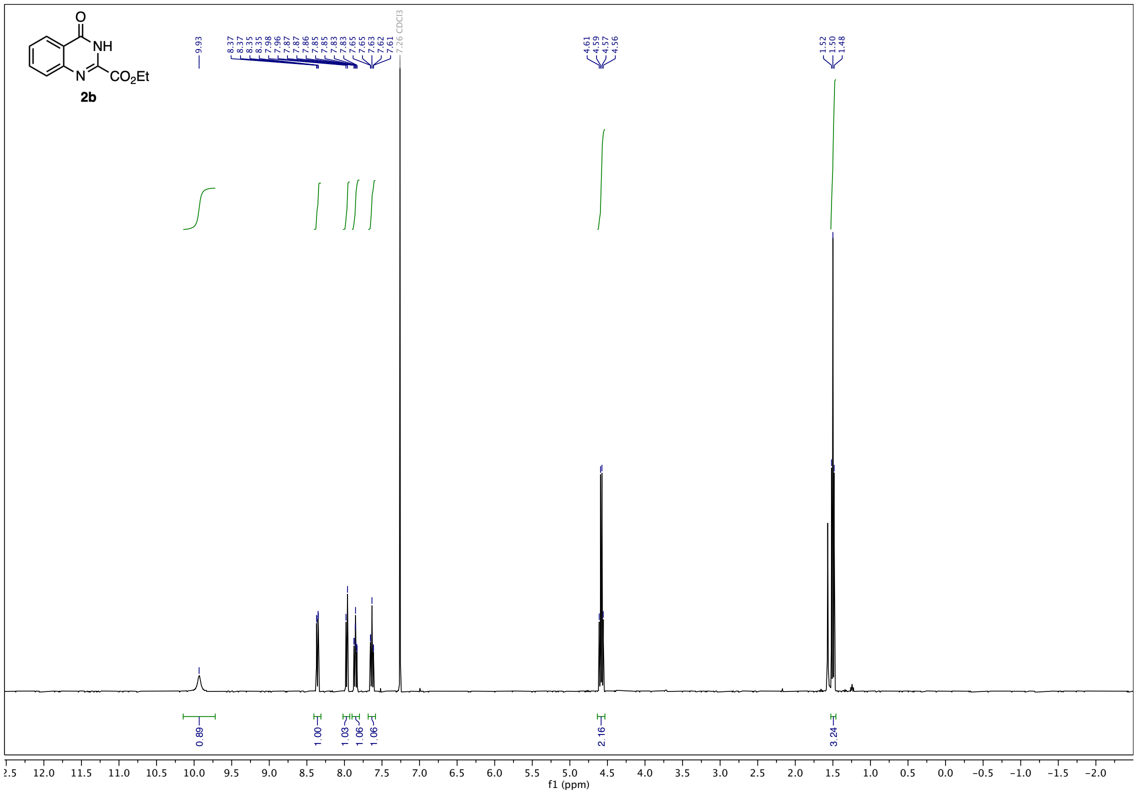

^{13}C{^{1}H} NMR (101 MHz, $CDCl_3$) of ethyl 4-oxo-3,4-dihydroquinazoline-2-carboxylate (**2b**)

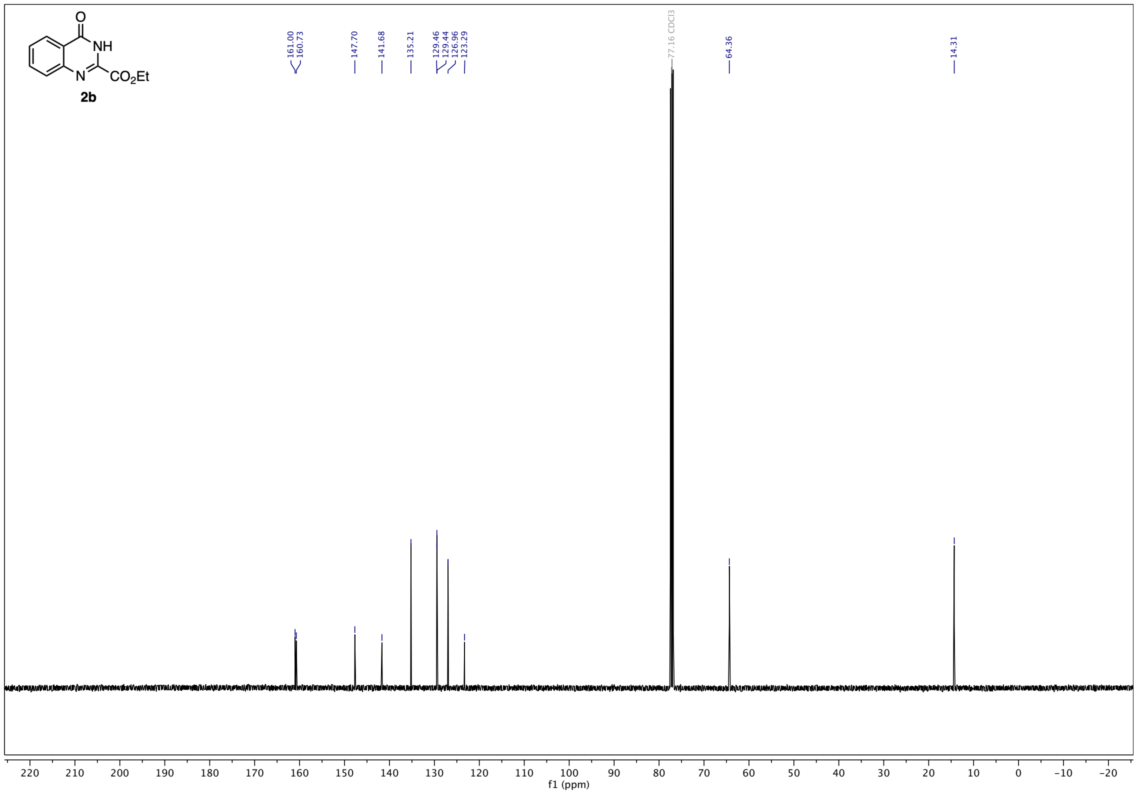

^{1}H NMR (400 MHz, $CDCl_3$) of ethyl 7-(*tert*-butyl)-4-oxo-3,4-dihydroquinazoline-2-carboxylate (**2d**)

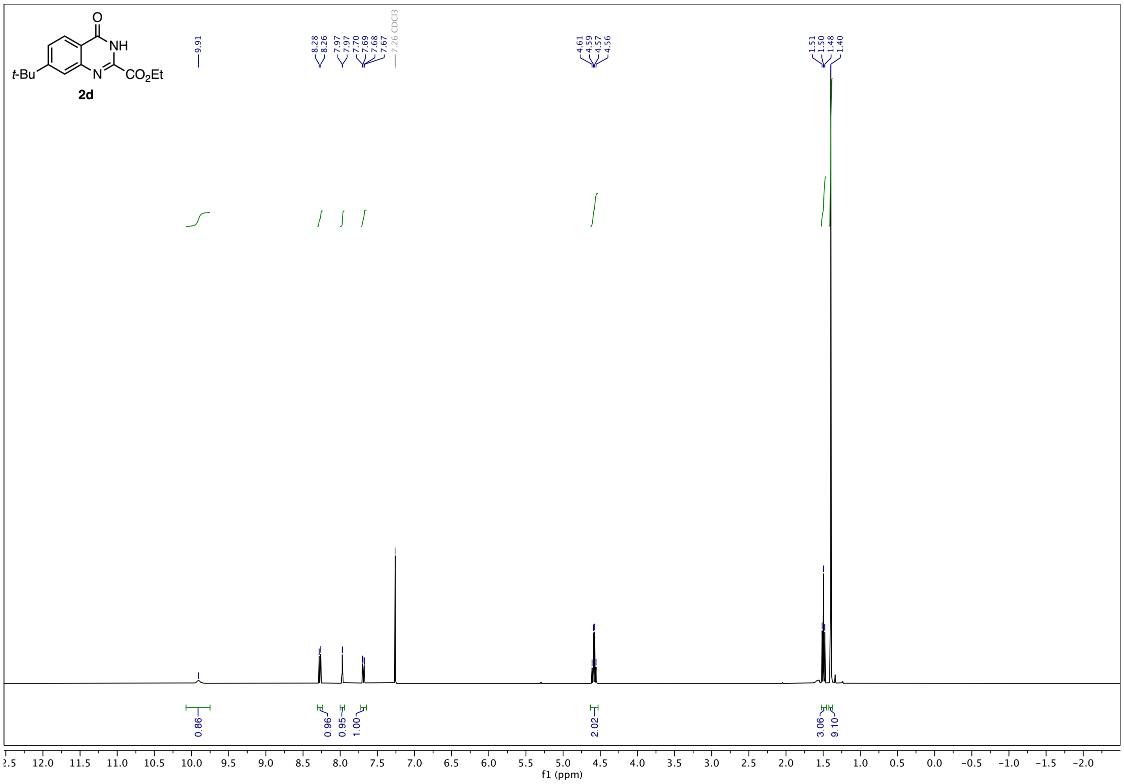

^{13}C{^{1}H} NMR (101 MHz, $CDCl_3$) of ethyl 7-(*tert*-butyl)-4-oxo-3,4-dihydroquinazoline-2-carboxylate (**2d**)

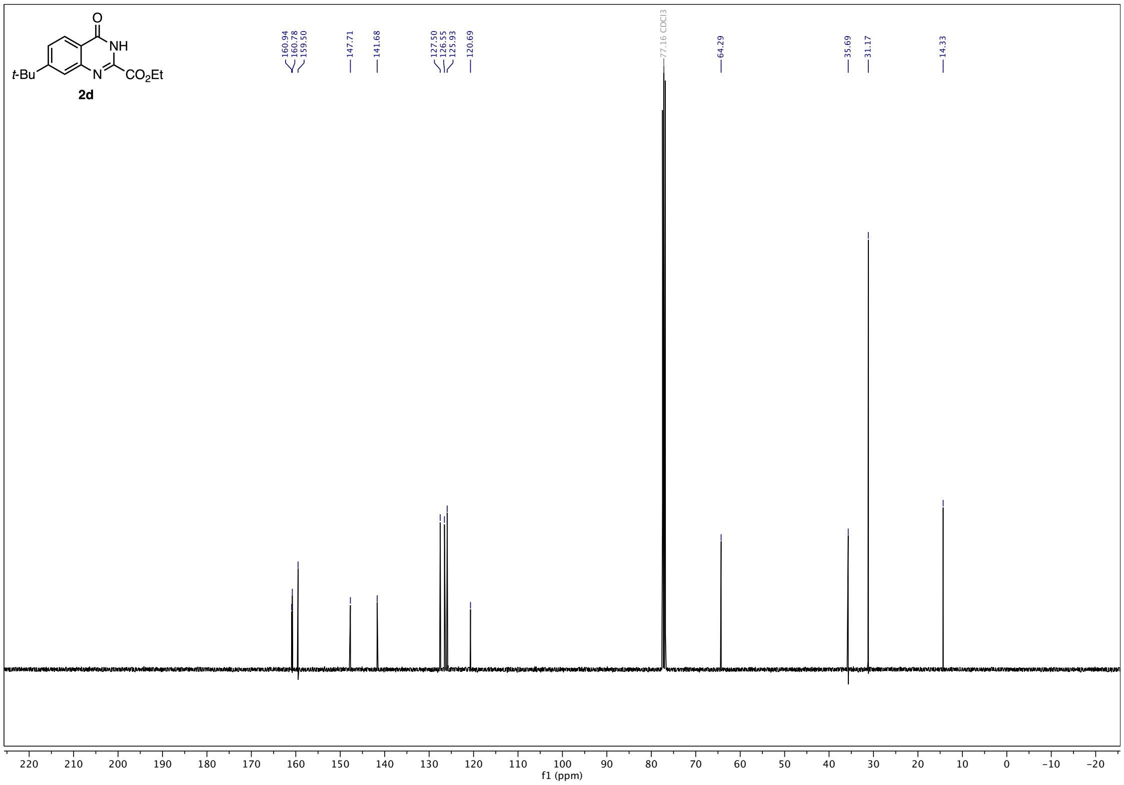

^{1}H NMR (400 MHz, $CDCl_3$) of ethyl 7-methoxy-4-oxo-3,4-dihydroquinazoline-2-carboxylate (**2e**)

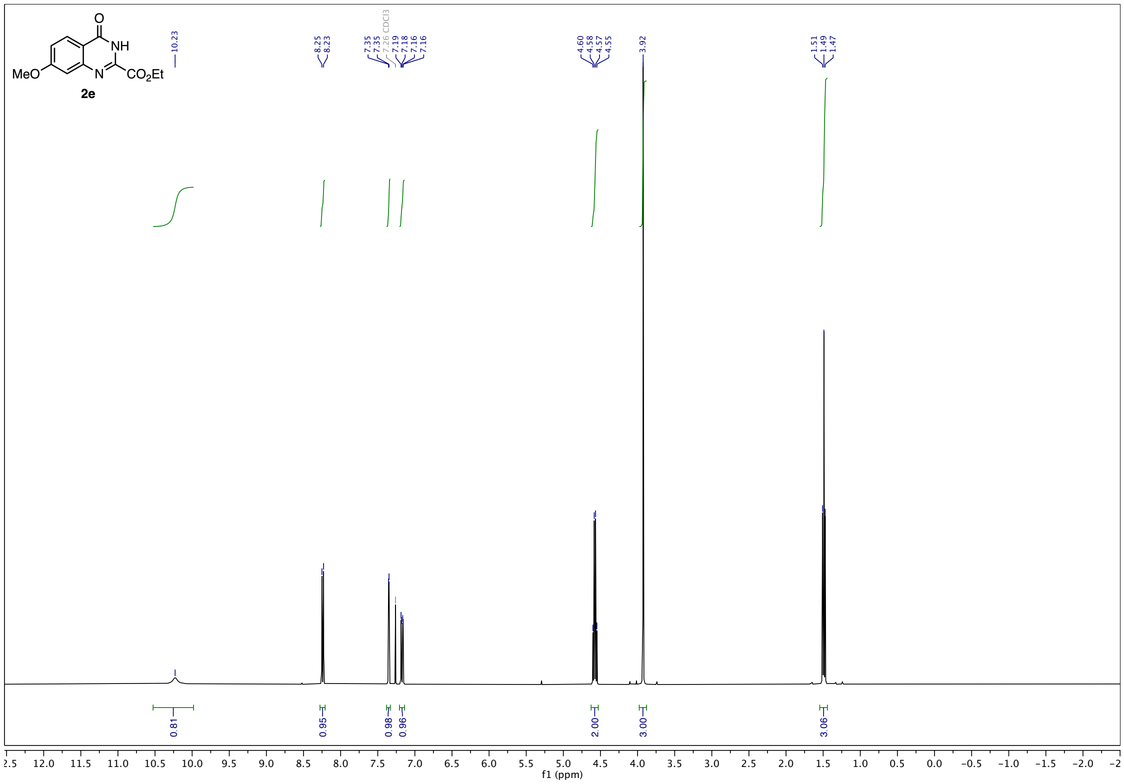

$^{13}C\{^{1}H\}$ NMR (101 MHz, $CDCl_3$) of ethyl 7-methoxy-4-oxo-3,4-dihydroquinazoline-2-carboxylate (**2e**)

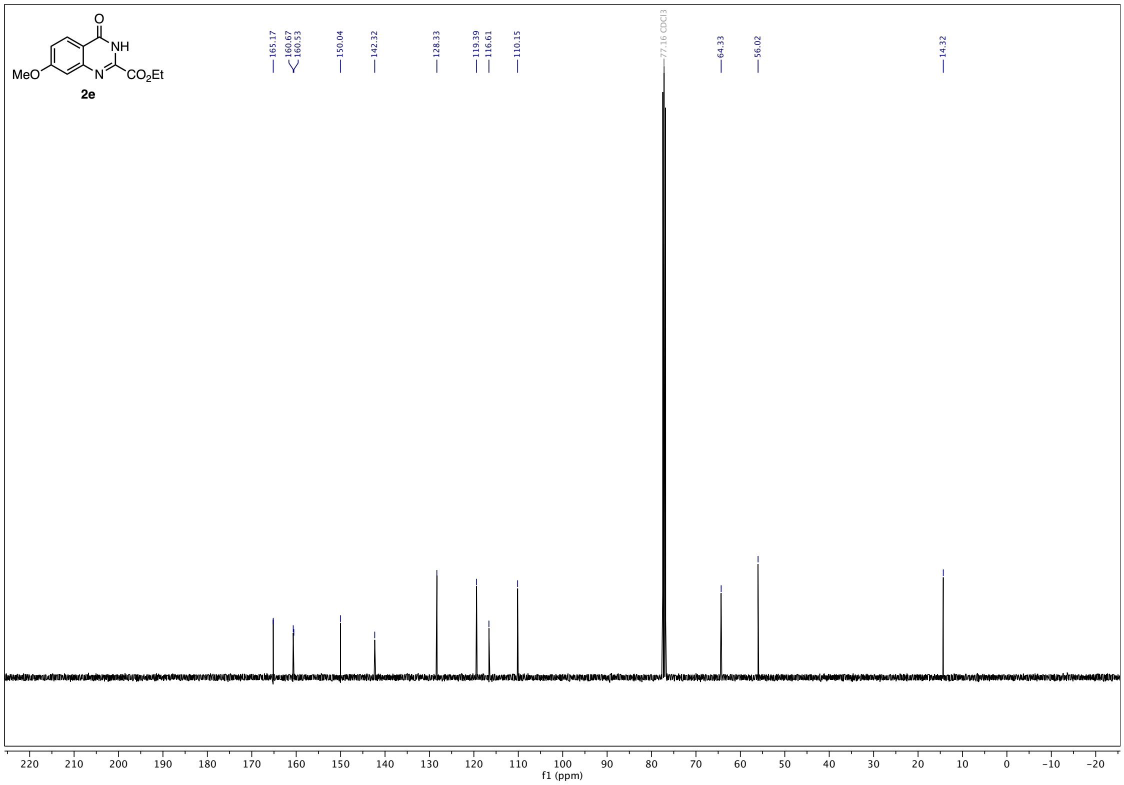

^{1}H NMR (400 MHz, $CDCl_3$) of ethyl 7-bromo-4-oxo-3,4-dihydroquinazoline-2-carboxylate (**2f**)

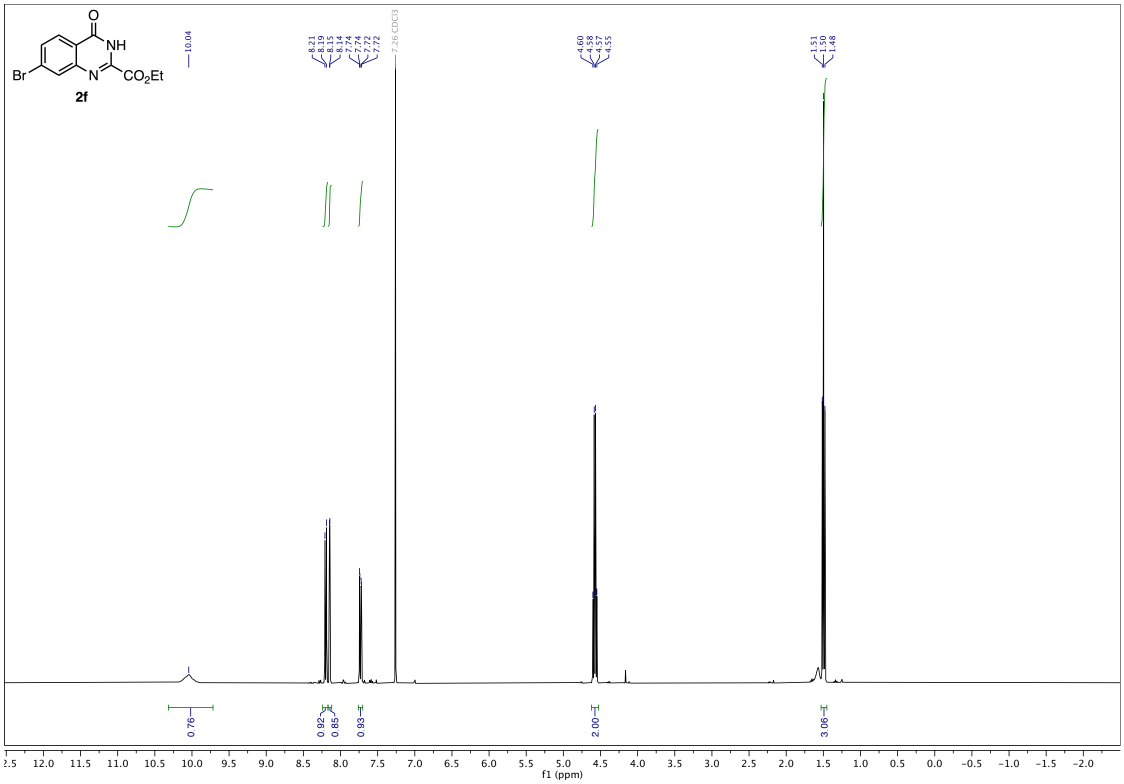

^{13}C{^{1}H} NMR (101 MHz, $CDCl_3$) of ethyl 7-bromo-4-oxo-3,4-dihydroquinazoline-2-carboxylate (**2f**)

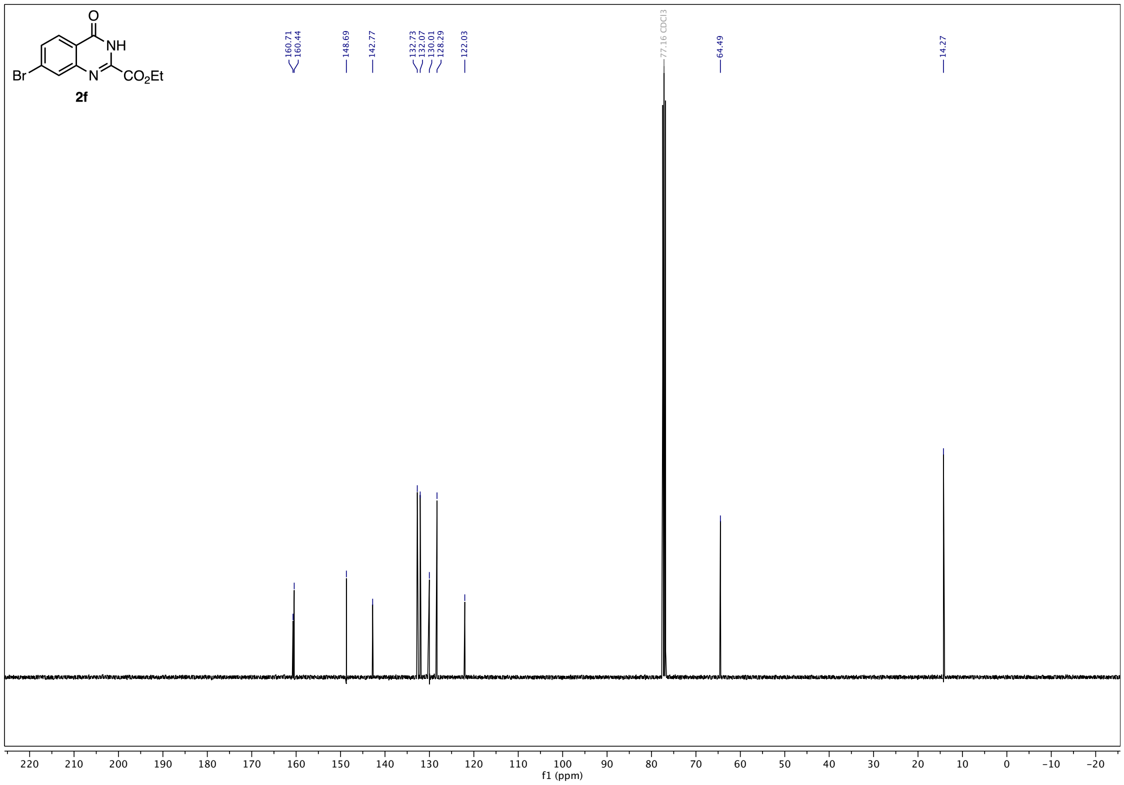

^{1}H NMR (400 MHz, $CDCl_3$) of ethyl 4-oxo-7-(trifluoromethyl)-3,4-dihydroquinazoline-2-carboxylate (**2g**)

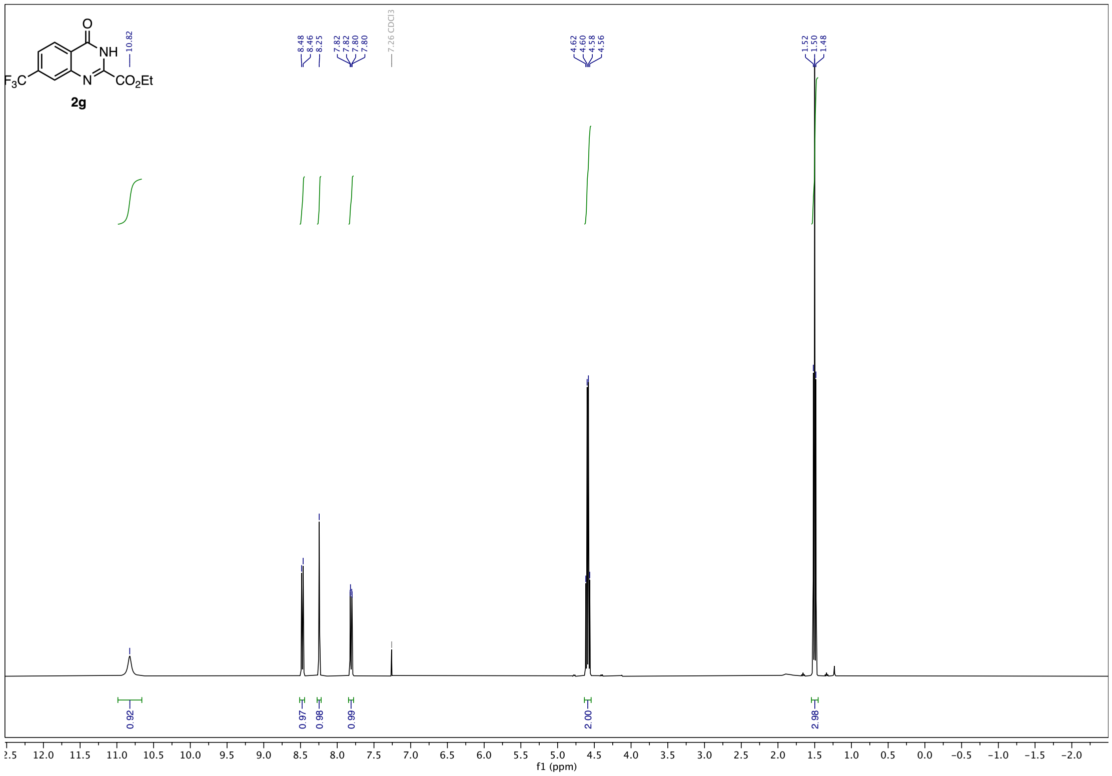

$^{13}C\{^{1}H\}$ NMR (101 MHz, $CDCl_3$) of ethyl 4-oxo-7-(trifluoromethyl)-3,4-dihydroquinazoline-2-carboxylate (**2g**)

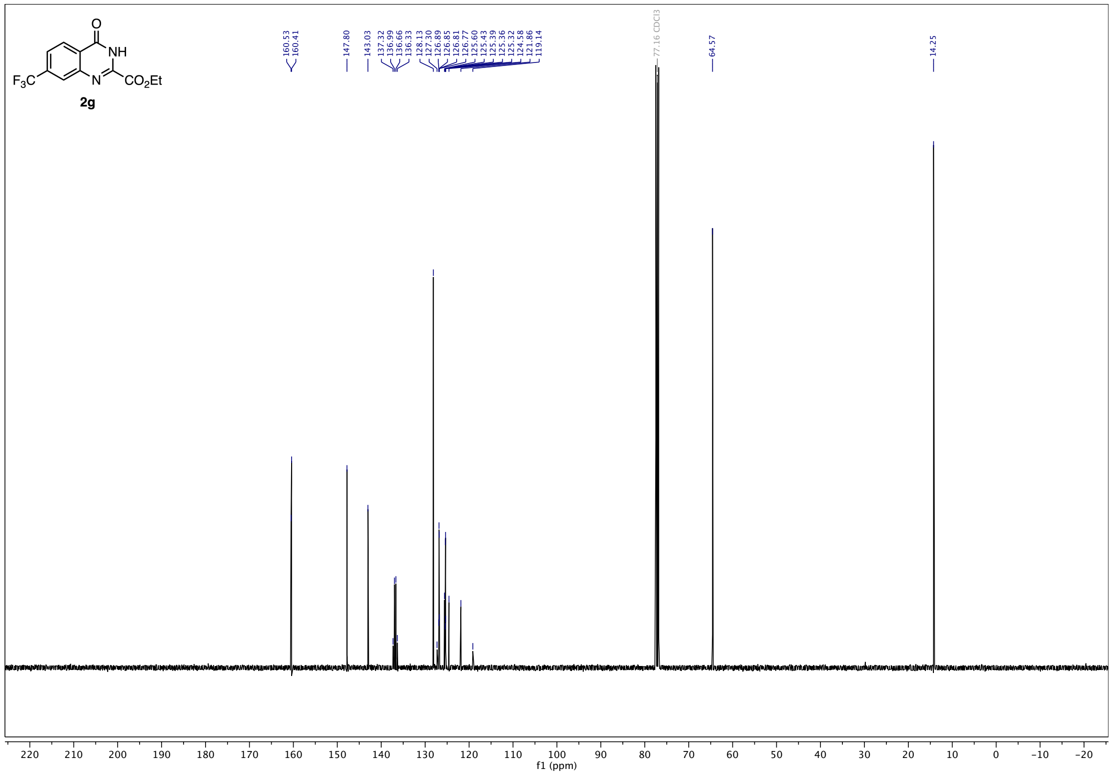

^{19}F NMR (376 MHz, $CDCl_3$) of ethyl 4-oxo-7-(trifluoromethyl)-3,4-dihydroquinazoline-2-carboxylate (**2g**)

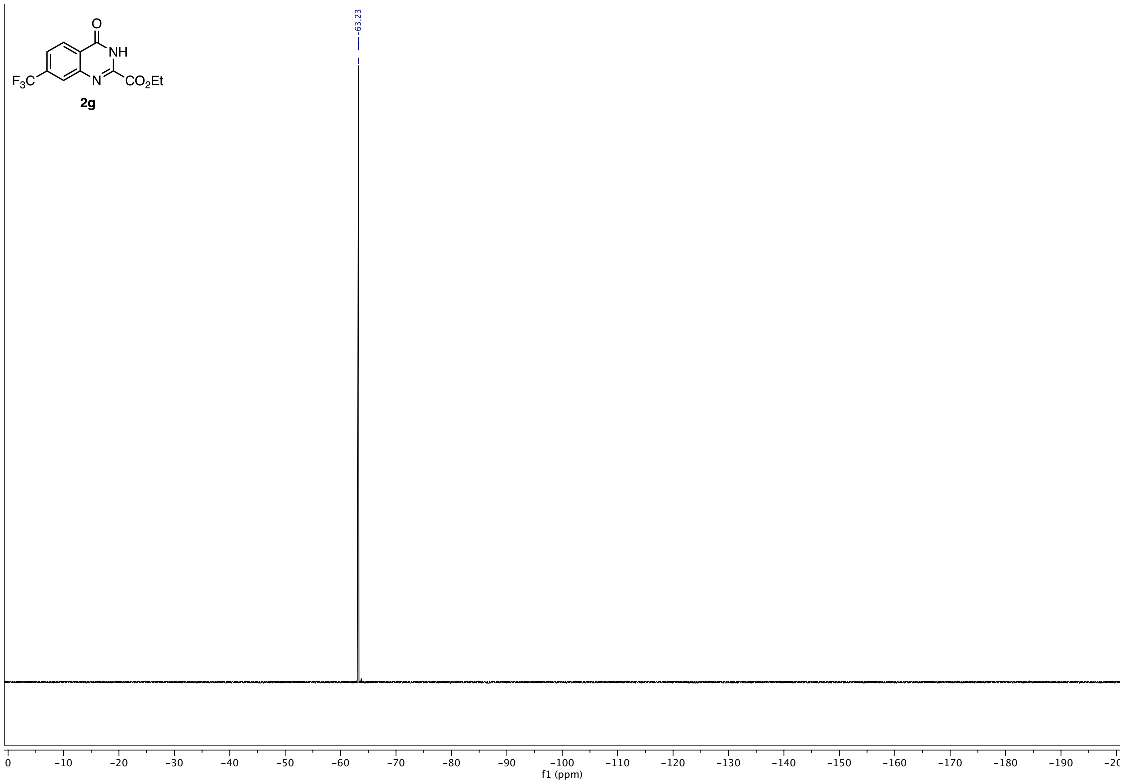

^{1}H NMR (400 MHz, $CDCl_3$) of ethyl 7-cyano-4-oxo-3,4-dihydroquinazoline-2-carboxylate (**2h**)

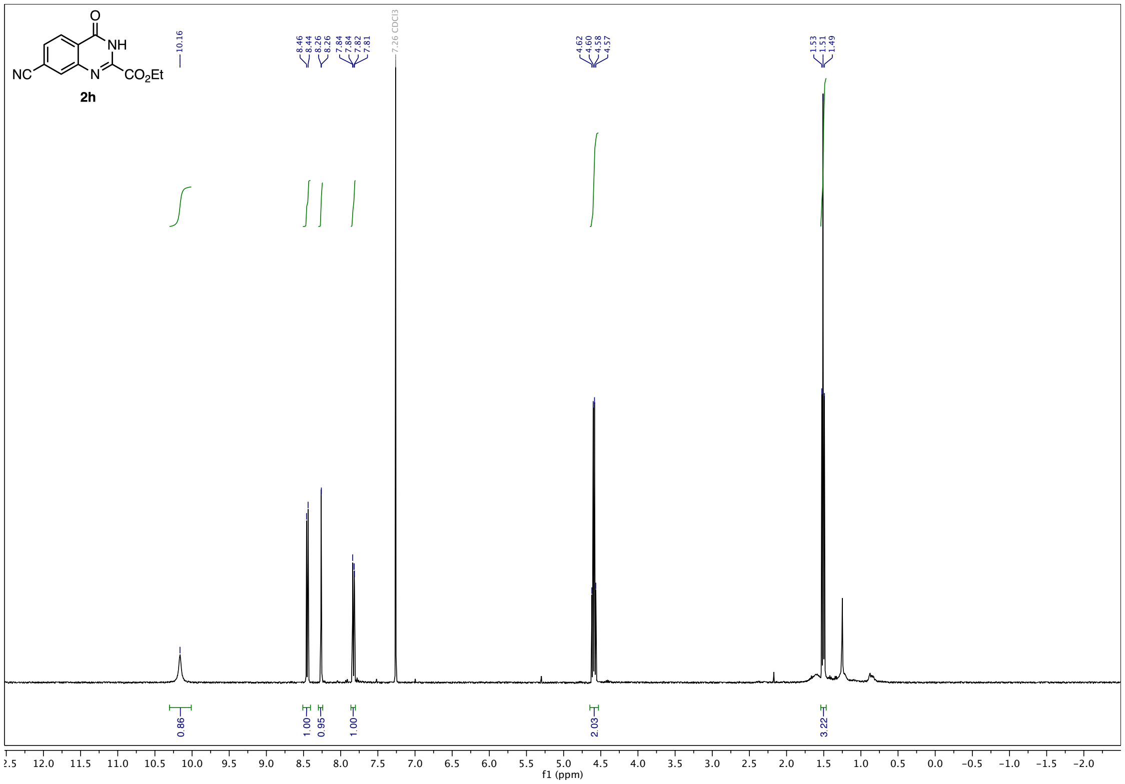

$^{13}C\{^{1}H\}$ NMR (101 MHz, $CDCl_3$) of ethyl 7-cyano-4-oxo-3,4-dihydroquinazoline-2-carboxylate (**2h**)

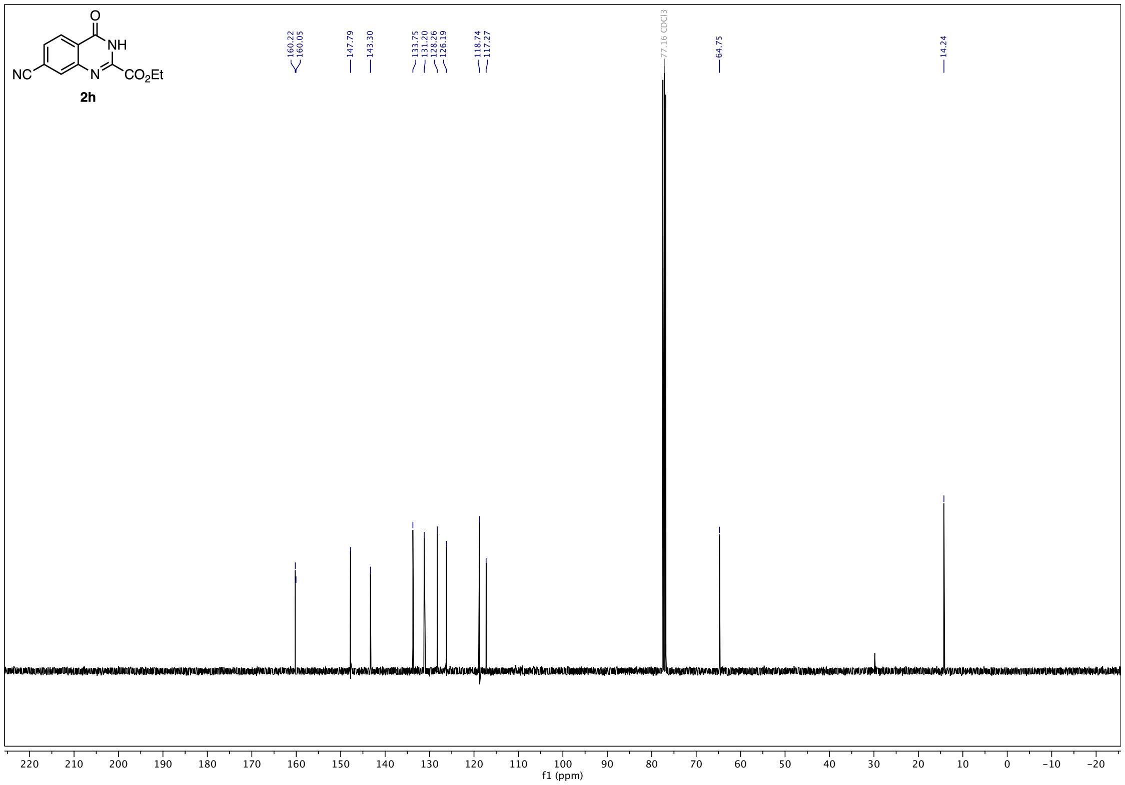

^{1}H NMR (400 MHz, $CDCl_3$) of **2i-8** and **2i-6**

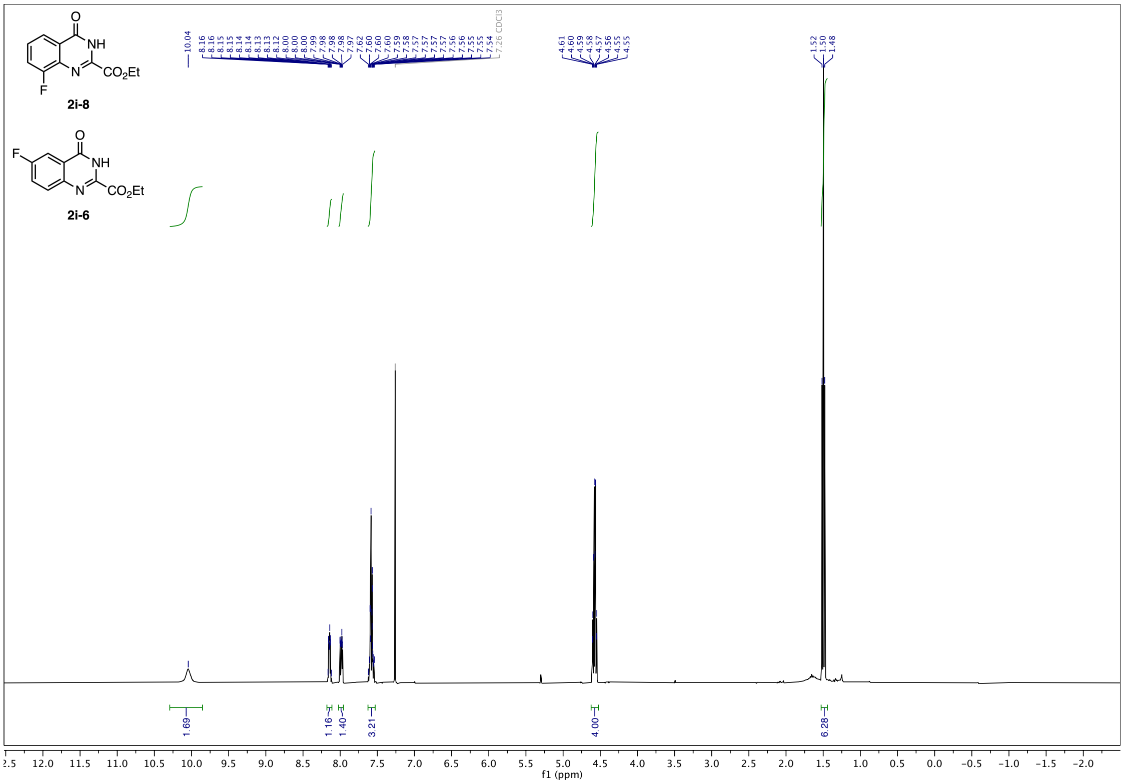

$^{13}C\{^{1}H, ^{19}F\}$ NMR (101 MHz, $CDCl_3$) of **2i-8** and **2i-6**

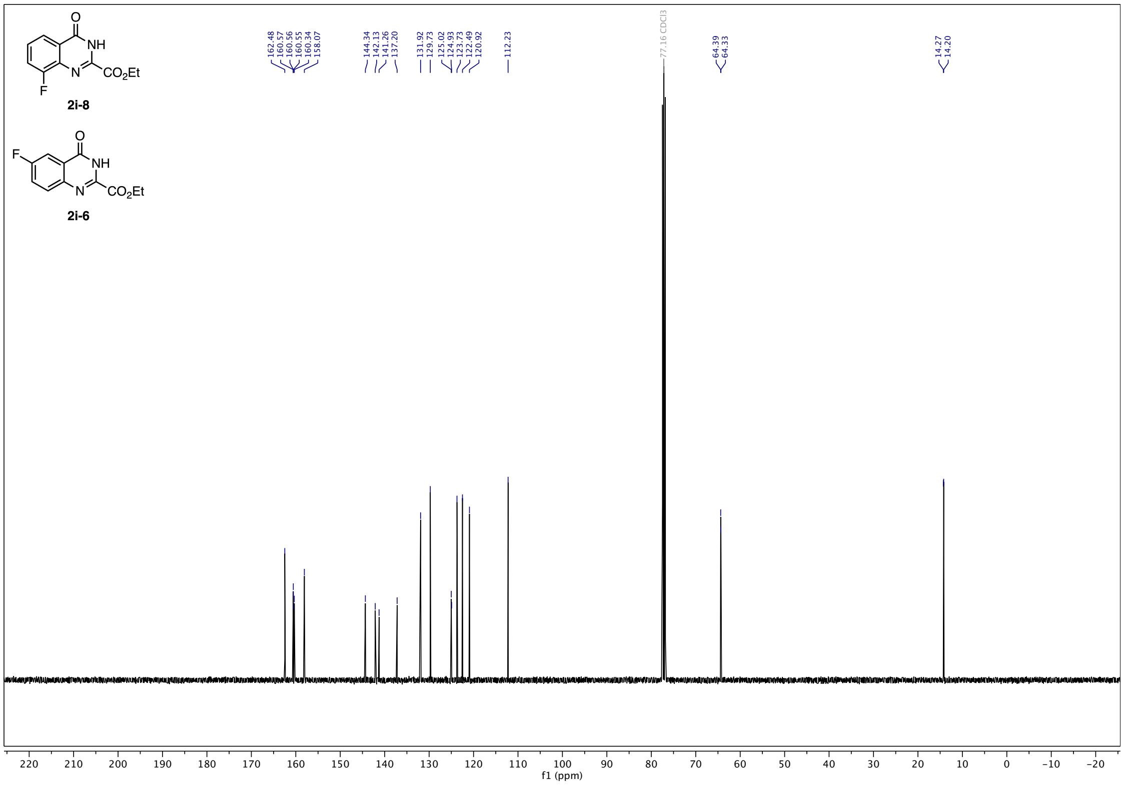

^{19}F NMR (376 MHz, $CDCl_3$) of **2i-8** and **2i-6**

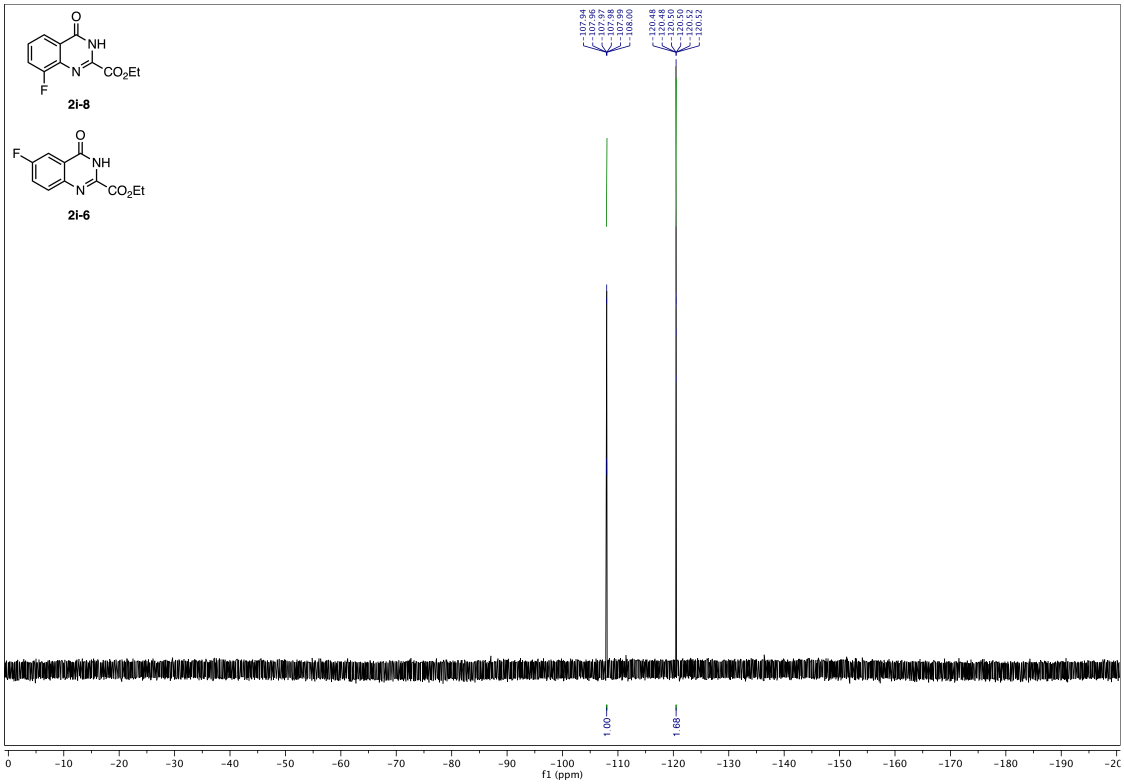

^1H NMR (400 MHz, $CDCl_3$) of ethyl 5-chloro-4-oxo-3,4-dihydroquinazoline-2-carboxylate (**2j**)

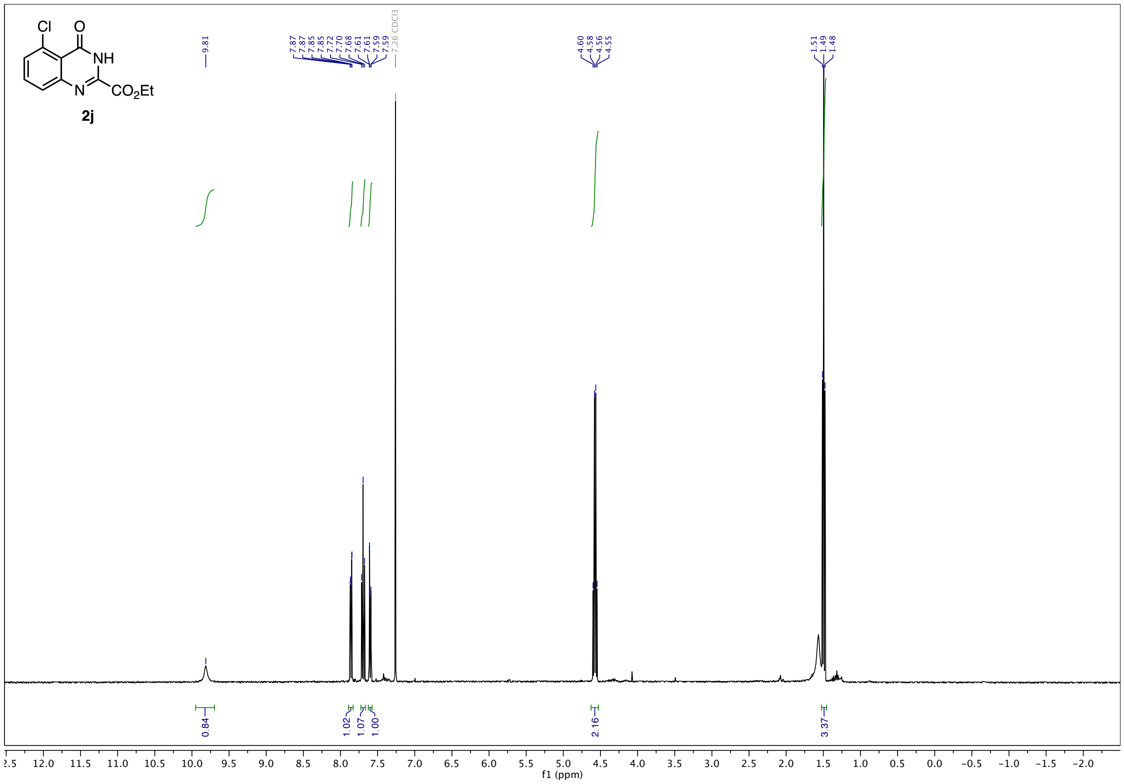

$^{13}C\{^1H\}$ NMR (101 MHz, $CDCl_3$) of ethyl 5-chloro-4-oxo-3,4-dihydroquinazoline-2-carboxylate (**2j**)

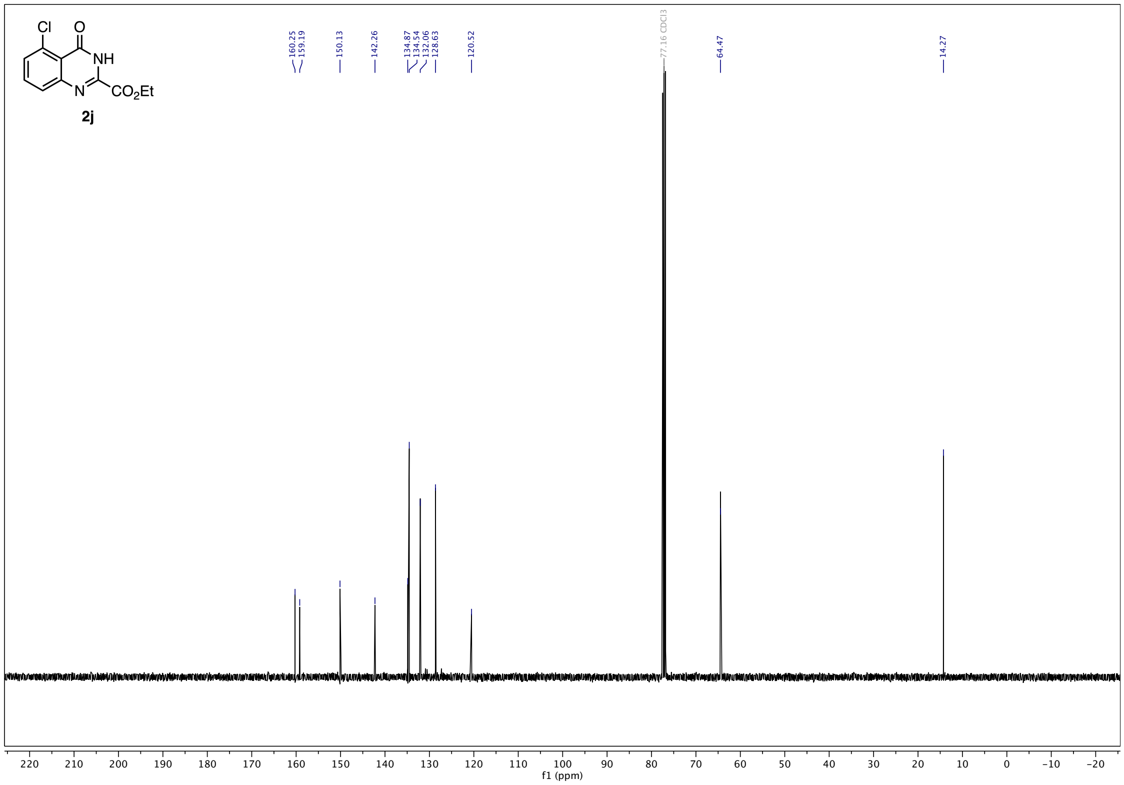

^{1}H NMR (400 MHz, $CDCl_3$) of 2-(pyridin-2-yl)quinazolin-4(3*H*)-one (**2k**)

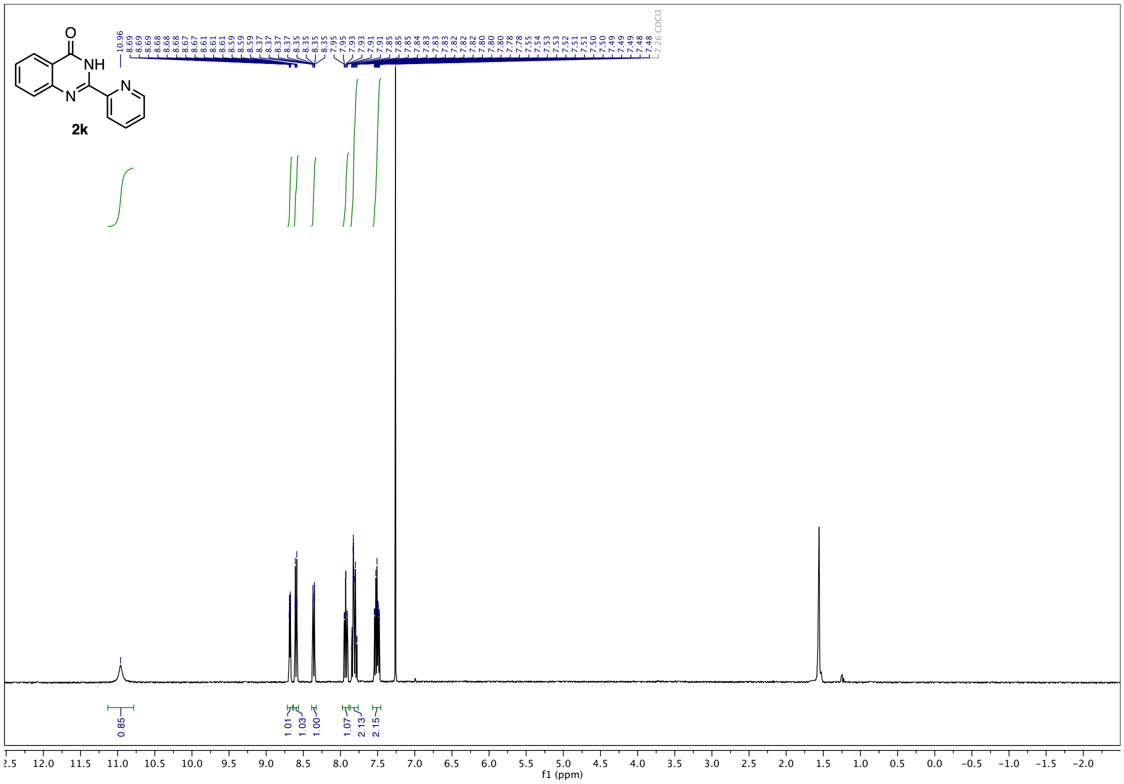

$^{13}C\{^{1}H\}$ NMR (101 MHz, $CDCl_3$) of 2-(pyridin-2-yl)quinazolin-4(3*H*)-one (**2k**)

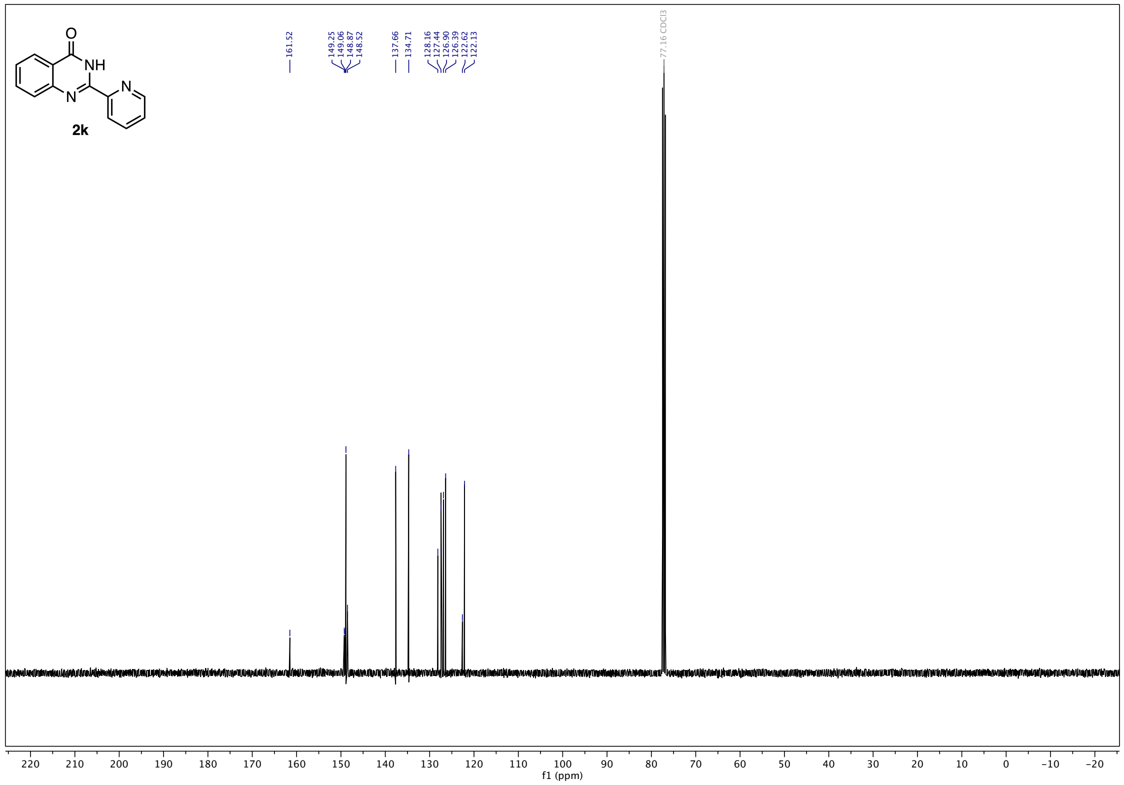

^{1}H NMR (400 MHz, DMSO-d_6) of 2-propylquinazolin-4(3*H*)-one (**2l**)

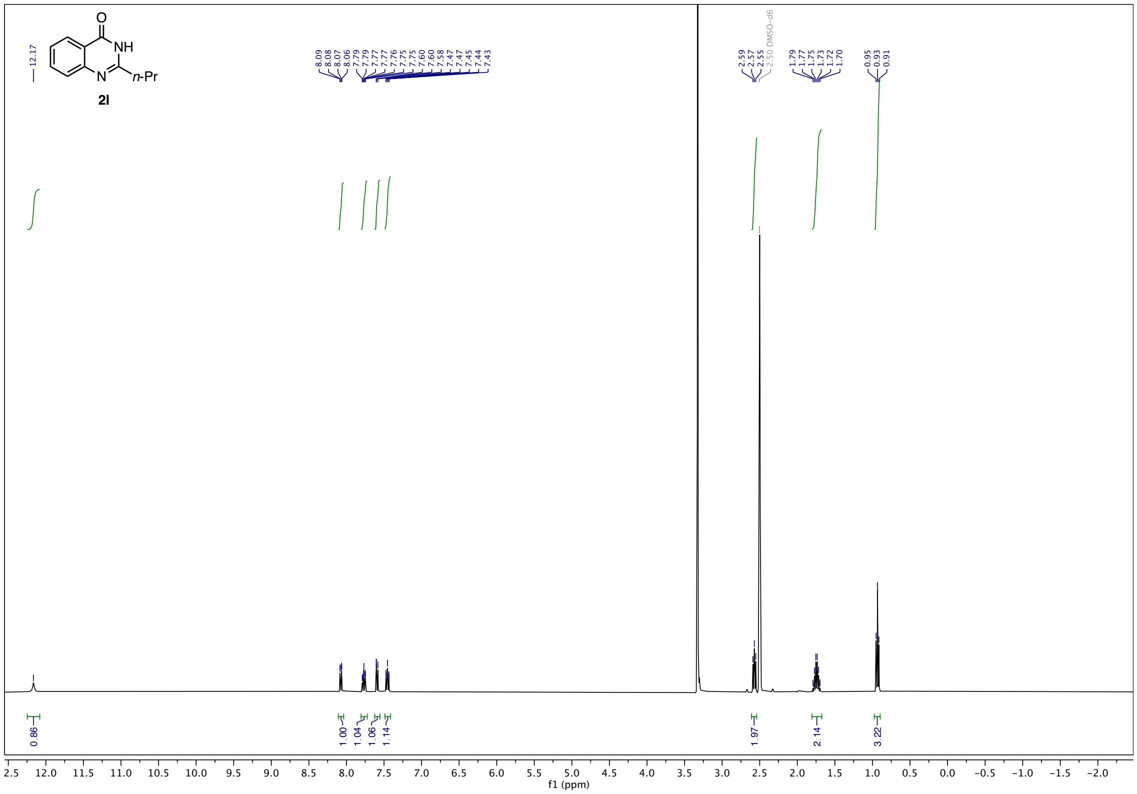

^{13}C{^{1}H} NMR (101 MHz, DMSO-d_6) of 2-propylquinazolin-4(3*H*)-one (**2l**)

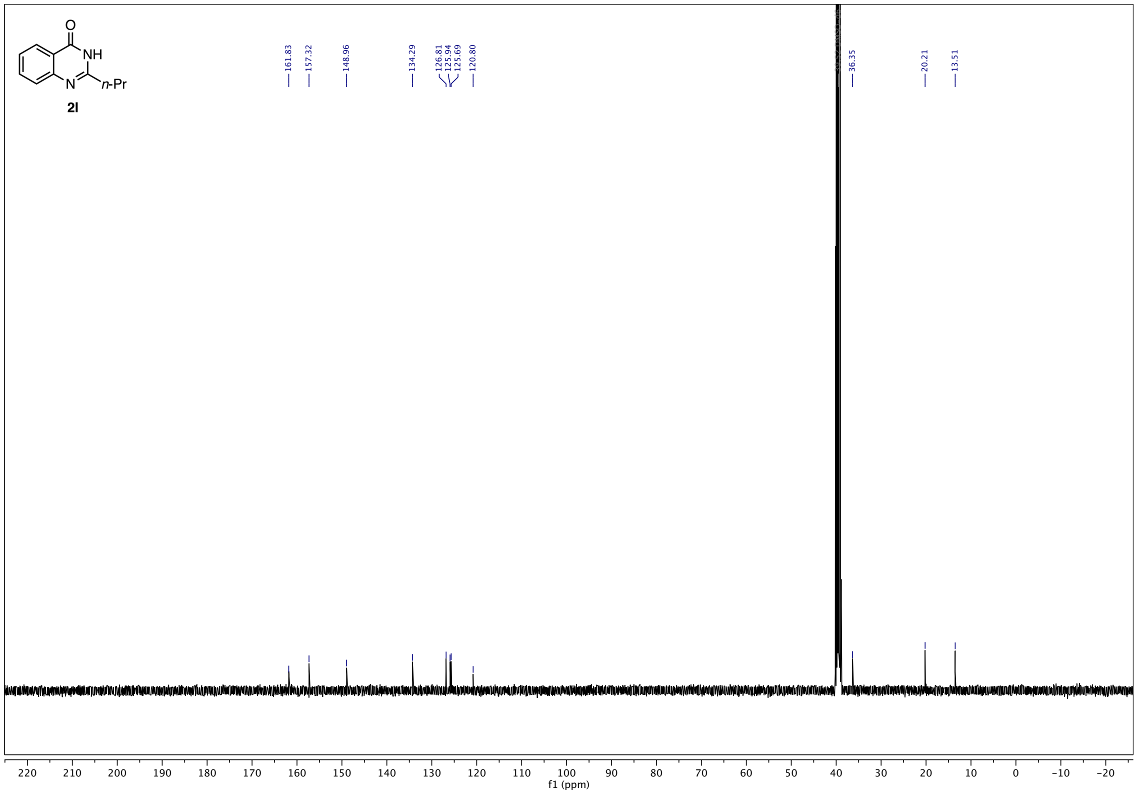

^{1}H NMR (400 MHz, $CDCl_3$) of 2,3-dihydropyrrolo[2,1-*b*]quinazolin-9(1*H*)-one (**2m**)

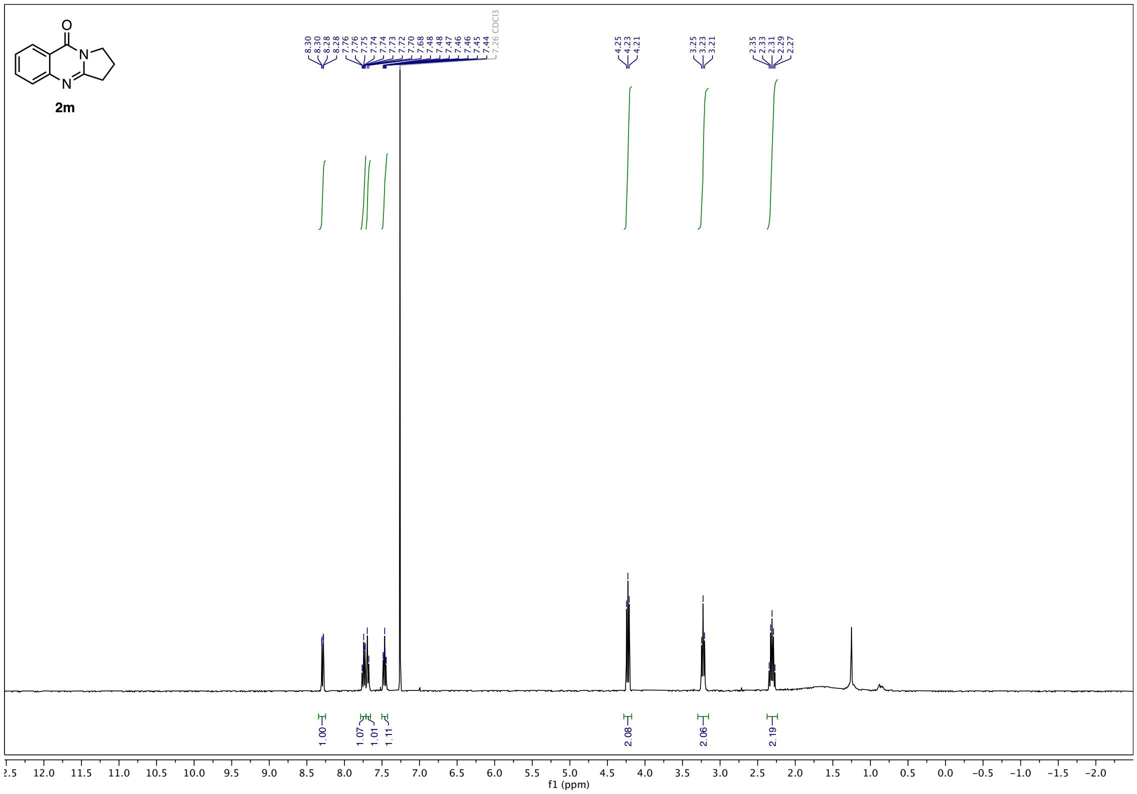

^{13}C{^{1}H} NMR (101 MHz, $CDCl_3$) of 2,3-dihydropyrrolo[2,1-*b*]quinazolin-9(1*H*)-one (**2m**)

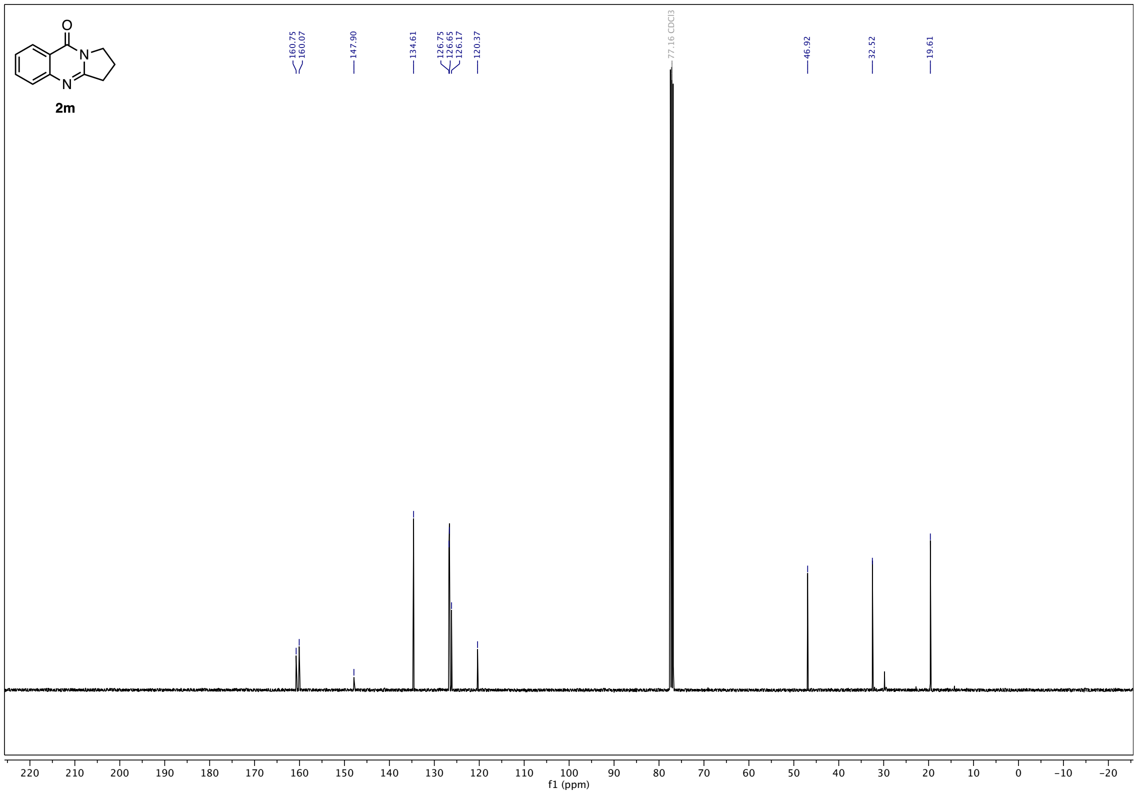

VI. References

(1) Bräse, S.; Gil, C.; Knepper, K.; Zimmermann, V. Organic Azides: An Exploding Diversity of a Unique Class of Compounds. *Angew. Chem. Int. Ed.* **2005**, *44* (33), 5188–5240. DOI: 10.1002/anie.200400657.

(2) University of California Santa Barbara. Laboratory Safety Fact Sheet #26: Synthesizing, Purifying, and Handling Organic Azides. http://www.ehs.ucsb.edu/files/docs/ls/factsheets/Azides_FS26.pdf.

(3) Yang, T.; Wang, W.; Wei, D.; Zhang, T.; Han, B.; Yu, W. Synthesis of quinazolinones *via* radical cyclization of α-azidyl benzamides. *Org. Chem. Front.* **2017**, *4* (3), 421–426. DOI: 10.1039/c6qo00656f.

(4) Chau, J.; Zhang, J.; Ciufolini, M. A. A Peterson avenue to 5-alkenyloxazoles. *Tetrahedron Lett.* **2009**, *50* (45), 6163–6165. DOI: 10.1016/j.tetlet.2009.08.076.

(5) Hafez, A. M.; Taggi, A. E.; Dudding, T.; Lectka, T. Asymmetric Catalysis on Sequentially-Linked Columns. *J. Am. Chem. Soc.* **2001**, *123* (44), 10853–10859. DOI: 10.1021/ja016556j.

(6) Pedersen, C. M.; Marinescu, L. G.; Bols, M. Radical substitution with azide: $TMSN_3$–$PhI(OAc)_2$ as a substitute of IN_3. *Org. Biomol. Chem.* **2005**, *3* (5), 816–822. DOI: 10.1039/B500037H.

(7) Zhang, Y.; Dai, Y.; Li, G.; Cheng, X. The Catalytic Synthesis of Carboniolamide: The Role of *sp*^{3} Hybridized Oxygen. *Synlett* **2014**, *25* (18), 2644–2648. DOI: 10.1055/s-0034-1379101

(8) Garg, P.; Singh, T.; Singh, A. Visible light-mediated ring-ablative functionalization of oxazoles: oxidative azidation and demethylative amination. *Chem. Commun.* **2023**, *59* (61), 9360–9363. DOI: 10.1039/d3cc01871g.

(9) Koch, C.; Kahnes, M.; Schulz, M.; Görls, H.; Westerhausen, M. Lithium and Zinc Complexes of *C*- and *N*-Functionalized (2-Pyridylmethyl)amines. *Eur. J. Inorg. Chem.* **2008**, *2008* (7), 1067–1077. DOI: 10.1002/ejic.200701168.

(10) Bielinski, M.; Henderson, L. R.; Yosaatmadja, Y.; Swift, L. P.; Baddock, H. T.; Bowen, M. J.; Brem, J.; Jones, P. S.; McElroy, S. P.; Morrison, A.; Speake, M.; van Boeckel, S.; van Doornmalen, E.; van Groningen, J.; van den Hurk, H.; Gileadi, O.; Newman, J. A.; McHugh, P. J.; Schofield, C. J. Cell-active small molecule inhibitors validate the SNM1A DNA repair nuclease as a cancer target. *Chem. Sci.* **2024**, *15* (21), 8227–8241. DOI: 10.1039/d4sc00367e.

(11) Li, Q.; Huang, Y.; Chen, T.; Zhou, Y.; Xu, Q.; Yin, S.-F.; Han, L.-B. Copper-Catalyzed Aerobic Oxidative Amination of sp^{3}C–H Bonds: Efficient Synthesis of 2-Hetarylquinazolin-4(3*H*)-ones. *Org. Lett.* **2014**, *16* (14), 3672–3675. DOI: 10.1021/ol501454j.

(12) Chen, X.; Xu, Y.; Zhao, W.; Wang, X.; Shi, Q.; Zhang, S.; Li, F. 2D Aza-COF-Supported Ruthenium Complex: A Recyclable Auto-Tandem Catalyst for the Preparation of Quinazolinones from Allylic Alcohols and *o*-Aminobenzamides. *Org. Lett.* **2025**, *27* (15), 3858–3864. DOI: 10.1021/acs.orglett.5c00529.

(13) Murai, K.; Komatsu, H.; Nagao, R.; Fujioka, H. Oxidative Rearrangement of Spiro Cyclobutane Cyclic Aminals: Efficient Construction of Bicyclic Amidines. *Org. Lett.* **2012**, *14* (3), 772–775. DOI: 10.1021/ol203313n.
